# LAPTM4B is associated with poor prognosis in NSCLC and promotes the NRF2-mediated stress response pathway in lung cancer cells

**DOI:** 10.1038/srep13846

**Published:** 2015-09-07

**Authors:** Yuho Maki, Junya Fujimoto, Wenhua Lang, Li Xu, Carmen Behrens, Ignacio I. Wistuba, Humam Kadara

**Affiliations:** 1 Department of Translational Molecular Pathology, The University of Texas MD Anderson Cancer Center, Houston, TX, U.S.A; 2 Department of Thoracic/Head and Neck Medical Oncology, The University of Texas MD Anderson Cancer Center, Houston, TX, U.S.A; 3 The University of Texas Graduate School of Biomedical Sciences, Houston, TX, U.S.A

## Abstract

We recently demonstrated that lysosomal protein transmembrane 4 beta (*LAPTM4B*) is elevated in non-small cell lung cancers (NSCLCs) and in the surrounding premalignant airway field of cancerization. In the present study, we sought to begin to understand the relevance of *LAPTM4B* expression and signaling to NSCLC pathogenesis. *In situ* hybridization analysis of *LAPTM4B* transcript in tissue microarrays comprised of 368 NSCLCs demonstrated that *LAPTM4B* expression was significantly increased in smoker compared to non-smoker lung adenocarcinoma tumors (*P* < 0.001) and was significantly associated with poor overall survival (*P* < 0.05) in adenocarcinoma patients. Knockdown of *LAPTM4B* expression inhibited cell growth, induced cellular apoptosis and decreased cellular autophagy in serum starved lung cancer cells. Expression profiling coupled with pathways analysis revealed decreased activation of the nuclear factor erythroid 2-like 2 (*NRF2*) stress response pathway following *LAPTM4B* knockdown. Further analysis demonstrated that *LAPTM4B* augmented the expression and nuclear translocation of the *NRF2* transcription factor following serum deprivation as well as increased the expression of *NRF2* target genes such as heme oxygenase 1/*HMOX1*). Our study points to the relevance of *LAPTM4B* expression to NSCLC pathogenesis as well as to the probable role of *LAPTM4B*/*NRF2* signaling in promoting lung cancer cell survival.

Lung cancer is the leading cause of cancer deaths in the United States and worldwide^1^,^2^. Non-small cell lung cancer (NSCLC) represents the majority (~85%) of all lung tumors, with lung adenocarcinomas (LUADs) and squamous cell carcinomas (SCCs) the most frequently diagnosed histological subtypes^3^. The high mortality of NSCLC is, in part, due to late diagnosis after regional or distant spread of the disease^4^,^5^. Even for early stage (stage-I) NSCLC, five-year survival rates reach only ~50% warranting the unmet need for better clinical management of NSCLC^4^,^5^. Despite this urgency, our understanding of NSCLC pathogenesis, which in turn is crucial for identification of new targets for prevention and treatment of this malignancy, is still lacking.

Previous work has suggested that lung carcinogenesis, to a large part, is a multistep process involving smoking-induced damage throughout the airway, a phenomenon termed airway field cancerization^6^,^7^. Genetic changes that are characteristic of lung tumors are present in adjacent visually normal-appearing airway epithelium^6^,^7^,^8^,^9^,^10^,^11^,^12^,^13^,^14^. These airway field cancerization effects provide powerful means to understand early molecular aberrations in lung cancer development^6^,^7^. A recent study by our group pinpointed genes in airway field cancerization that gradually increase or decrease with shorter distance of the airway from the nearby tumor and that are recapitulated in the NSCLCs^11^. Notably, our recent study demonstrated that lysosomal protein transmembrane 4 beta (*LAPTM4B*) is largely elevated in airways closest to tumors and in NSCLCs compared to normal lung tissues as well as promotes anchorage-independent growth of lung cancer cells^11^.

*LAPTM4B* is tetratransmembrane lysosomal protein^15^ that is over-expressed and associated with poor prognosis in various malignancies including ovarian, hepatocellular and prostate cancer^16^,^17^,^18^. Polymorphisms in *LAPTM4B* have been shown to be associated with susceptibility to various malignancies including breast and lung carcinomas^19^,^20^. Moreover, genomic amplification of *LAPTM4B* was demonstrated to be significantly associated with resistance to adjuvant chemotherapy in human primary breast cancer^21^. *LAPTM4B* was found to mediate breast cancer resistance to anthracycline therapy, in part, by decreasing trafficking of the drug to breast cancer cell nuclei^21^. In addition, *LAPTM4B* was shown in breast tumor cells to mediate formation of autolysosomes from fusion of lysosomes with autophagosomes, an essential step in activation of autophagy^22^, in response to metabolic and genotoxic stress^23^. More recently, *LAPTM4B* was found to facilitate the role of inactive epidermal growth factor receptor (*EGFR*) in autophagy initiation^24^.

The expression pattern of *LAPTM4B* in human NSCLC and the role of this putative oncogene in NSCLC pathogenesis and cell signaling remain elusive. In this study, we analyzed *LAPTM4B* expression in NSCLC histological tissue specimens in association with various clinicopathological variables and studied the impact of *LAPTM4B* expression on the malignant phenotype *in vitro*. We report that high *LAPTM4B* expression is indicative of poor survival in LUAD and that *LAPTM4B* protects cells from starvation-induced stress, promotes cellular autophagy and activates *NRF2*-mediated cell stress response and pathway suggesting that *LAPTM4B* may be a viable target for NSCLC therapy.

## Results

### *LAPTM4B* is up-regulated in smoker LUADs and associated with poor prognosis

**Figure 1:**
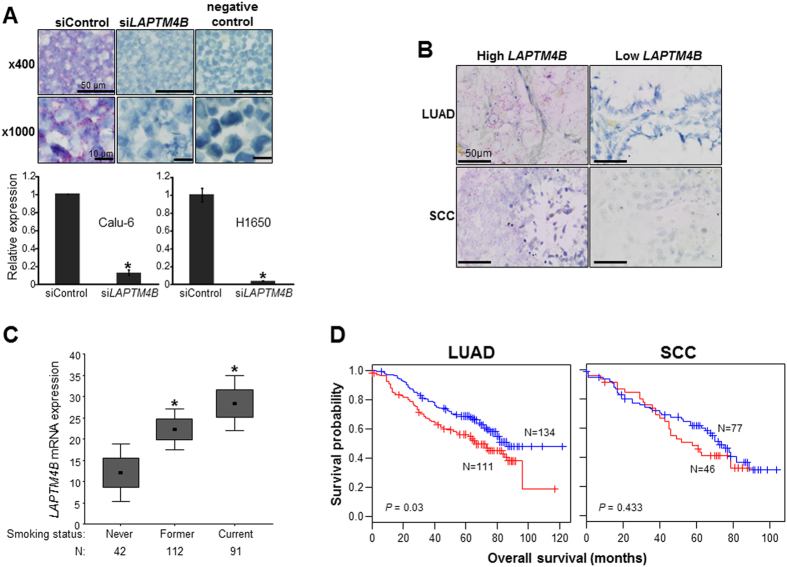
Increased *LAPTM4B* expression is associated with smoking and poor prognosis in lung adenocarcinoma. *LAPTM4B* mRNA expression was analyzed by ISH as described in the Materials and Methods section. (**A**) The ISH assay (upper panels) was first examined in pellets from Calu-6 cells transfected with control (left) and *LAPTM4B*-specific (middle) siRNAs. qRT-PCR analysis (lower panels and bar graphs) was used to confirm the efficacy of *LAPTM4B* siRNA-mediated knockdown. qRT-PCR analysis was performed in triplicates for all samples. *indicate *P*-values < 0.05 assessed by the Student’s t-test. (**B**) Representative photomicrographs depicting LUADs (upper) and SCCs (lower) exhibiting relatively high (left) and low (right) *LAPTM4B* mRNA expression following analysis of a NSCLC TMA comprised of 245 LUADs and 123 SCCs. (**C**) Box plots depicting mean *LAPTM4B* mRNA expression in non-smoker (never) and smoker (former and current) LUADs. *indicate *P*-values < 0.01 assessed by the Student’s t-test comparing each smoker group to the never smoker group. Boxes represent mean +/− standard errors. (**D**) Significant differences in overall survival between LUAD (left) and SCC (right) patients stratified by median *LAPTM4B* mRNA expression (red, higher; blue, lower) were statistically assessed by the log-rank test and Kaplan-Meier survival probability method. LUAD, lung adenocarcinoma; SCC, lung squamous cell carcinoma.

We recently found that *LAPTM4B* is an airway field cancerization marker that is largely elevated in NSCLCs and the surrounding airway epithelial field^11^ indicating that *LAPTM4B* may play important roles in NSCLC pathogenesis. The expression pattern of *LAPTM4B* in NSCLC specimens is unknown. Therefore, we sought to characterize *LAPTM4B* expression in a large series of NSCLC specimens in the context of various clinicopathological variables including patient outcome. We analyzed *LAPTM4B* expression by *in situ* hybridization (ISH) in a NSCLC (n = 368) tissue microarray (TMA, 245 LUADs and 123 SCCs) derived from patients (Supplementary Table S1) who did not receive neoadjuvant treatment. Detection of *LAPTM4B* by ISH was confirmed using fixed sections of Calu-6 cells transfected with control and *LAPTM4*B-siRNA and cells examined similarly except for omission of the *LAPTM4B* probe served as a negative control for the ISH assay (Fig. 1A, upper panels). *LAPTM4B* expression by ISH was markedly reduced in cells transfected with *LAPTM4B*-specific siRNA (Fig. 1A, upper panels) which was corroborated by quantitative real-time PCR (qRT-PCR) analysis (Fig. 1A, lower panels) and by western blotting analysis (Supplementary Figure S1). Fig. 1B depicts representative photomicrographs of LUAD (upper panels) and SCC (lower panels) specimens with relatively high (left panels) and low (right panels) *LAPTM4B* mRNA which was found to be confined to epithelial tumor cells and absent in the stroma. LUADs and SCCs exhibited similar average *LAPTM4B* expression scores by ISH and which were not significantly different among the two histologies (Supplementary Table S2). Notably, *LAPTM4B* was significantly higher in smokers (former or current smokers) compared to non-smoker LUADs (*P* < 0.01, Fig. 1C). Additionally, one-way ANOVA demonstrated a significant trend in elevated *LAPTM4B* expression with highest expression in current smokers and lowest in non-smokers (*P* < 0.01). We then examined association of *LAPTM4B* with survival in the 245 LUADs and 123 SCCs we had analyzed by ISH. LUAD patients with relatively higher (greater than the median) *LAPTM4B* mRNA expression exhibited significantly worse overall survival (*P* < 0.05 of the log-rank test) in comparison to patients with relatively lower *LAPTM4B* mRNA (Fig. 1D). These findings suggest that *LAPTM4B* field cancerization marker is associated with poor clinical outcome in NSCLC.

### *LAPTM4B* protects cells from serum starvation induced growth inhibition and promotes autophagy

**Figure 2:**
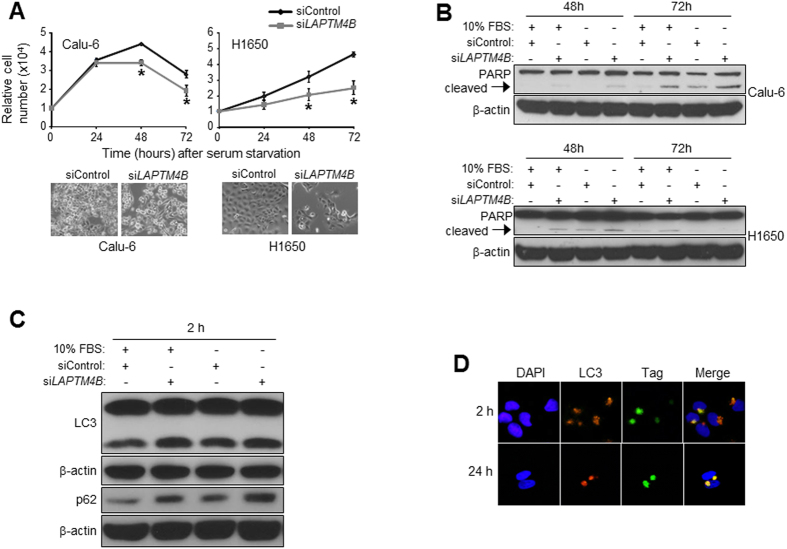
*LAPTM4B* knockdown suppresses cell growth and autophagy in serum-starved lung cancer cells. (**A**) Calu-6 and H1650 cells (5 × 10^4^ cells) were transfected with control or *LAPTM4B*-specific siRNAs and 24 h following transfection, cells were washed twice with 1x PBS and incubated in cell culture medium with 0% FBS for the indicated time points. Cell growth was analyzed by the Trypan blue exclusion method as described in the Materials and Methods section and plotted (upper panels). Lower panels depict representative bright field photomicrographs, obtained with a phase-contrast microscope at 10x, of Calu-6 (left) and H1650 (right) cells with and without *LAPTM4B* knockdown and at 72 h following serum deprivation. (**B**) Calu-6 (upper panel) and H1650 (lower panel) cells were transfected similarly as in (**A**) and 24 h following transfection were incubated in cell culture medium with or without 10% FBS for the indicated time points. For western blotting, 20 µg of total protein from samples were analyzed by SDS-PAGE as detailed in the Methods section. Membranes were stained with antibody against PARP and with an antibody against *β*-Actin monoclonal antibody to ensure equal protein loading. (**C**) Calu-6 cells were transfected as in (**A**) and then cultured with medium containing pepstatin A and EST in presence or absence of 10% FBS for 2 hours after which western blotting of total LC3 and p62 proteins was performed. (**D**) Calu-6 lung cancer cells were transfected with vectors coding for FLAG-tagged LAPTM4B as described in the Materials and Methods section. One day following transfection, cells were incubated in cell culture medium with 0% FBS after which they were analyzed by ICC for LC3 and LAPTM4B protein localization at 2 h and 24 h following serum starvation. All assays are representative of three independent experiments. * indicate *P*-values < 0.05 assessed by the Student’s t-test.

Earlier reports have demonstrated that *LAPTM4B* mediates breast cancer cell survival and promotes autophagy, through fusion of autophagosomes and lysosomes, following metabolic stress (e.g. nutrient deprivation)^21^,^23^. We were prompted to examine the impact of *LAPTM4B* expression on growth inhibitory effects of serum starvation in lung cancer cells. RNA interference-mediated knockdown of *LAPTM4B* significantly (*P* < 0.05) augmented cell growth inhibition induced by serum starvation (Fig. 2A) (upper panels, cell growth plots; lower panels, bright field images of cells at 72 h following serum deprivation and knockdown) irrespective of starting cell numbers (data not shown). It is important to note that we observed that Calu-6 cells, which typically exhibit mutations in the *KRAS* oncogene, were more sensitive to growth inhibitory effects of serum deprivation compared to *KRAS* wild type H1650 cells (Fig. 2A). In addition, western blotting analysis demonstrated that knockdown of *LAPTM4B* induced cleavage of poly (ADP) ribose polymerase 1 (PARP1) in cells cultured with serum as well as increased serum starvation-induced PARP cleavage (Fig. 2B) indicative of augmented apoptosis induction^25^,^26^. Additionally, PARP cleavage was more pronounced in *KRAS* mutant Calu-6 cells compared to *KRAS* wild type H1650 cells. The findings on PARP1 cleavage demonstrate that the cell growth inhibitory effects of *LAPTM4B* knockdown in serum-deprived cells may be, in part, attributed to cell death and apoptosis induction. We then sought to examine the potential implication of *LAPTM4B* in serum starvation-induced autophagy. Calu-6 lung cancer cells transfected with scrambled or *LAPTM4B*-targeting siRNA were cultured with medium containing pepstatin A and EST in the presence or absence of serum in order to study potential accumulation of LC3-II and p62/SQSTM1 autophagy markers^27^,^28^. Western blotting analysis demonstrated that both LC3-II and p62 protein levels were increased following knockdown of *LAPTM4B* (Fig. 2C) suggestive of accumulation of autophagosomes and inhibition of autolysosome formation. We then performed immunocytochemical (ICC) analysis in serum-starved cells over-expressing FLAG-tagged LAPTM4B protein. This analysis demonstrated that LAPTM4B protein co-localized with LC3 following serum starvation (Fig. 2D) suggestive of fusion of autophagosomes with lysosomes for continued autophagy flux. These findings corroborate earlier reports^23^ and demonstrate a cytoprotective role for *LAPTM4B* in promoting autophagy in lung cancer cells following cellular stressors such as nutrient deprivation.

### *LAPTM4B* suppression inhibits the *NRF2*-mediated stress response and pathway

**Figure 3:**
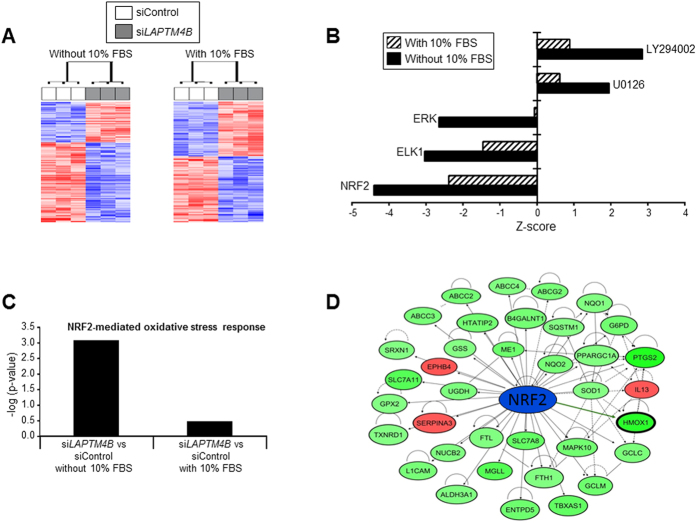
*LAPTM4B* knockdown suppresses the *NRF2* stress response and pathway as revealed by gene expression profiling and pathways analysis. Calu-6 cells were transfected with control or *LAPTM4B*-specific siRNAs. One day following transfection, cells were washed twice with 1x PBS and then incubated in cell culture medium with or without 10% FBS for 48 hours after which total RNA was isolated from the cells. All conditions were performed in triplicates (total n = 12 samples). Gene expression profiling was performed using the Affymetrix Human Gene 1.0 ST platform as detailed in the Materials and Methods section. (**A**) Heat maps depicting 1,252 (left, without 10% FBS) and 1,669 (right, with 10% FBS) gene features that were significantly differentially expressed, based on a *P* < 0.01, between cells transfected with scrambled (white) and *LAPTM4B*-specific (grey) siRNAs. Rows and columns represent gene features and samples, respectively. Up-regulated and down-regulated gene expression is indicated by red and blue colors, respectively. (**B**) Functional pathways analysis of the differentially expressed genes was performed using the IPA commercially available software. Significant (*P* < 0.001) modulation of upstream predicted regulators of the differentially expressed genes is indicated by the Z-score (negative and positive, inhibited and activated following *LAPTM4B* knockdown, respectively). (**C**) Statistically significant modulation (indicated by the inverse log of p-value) of the NRF2-mediated stress response and pathway as predicted by IPA is depicted. (**D**) Gene-interaction network mediated by *NRF2* that was topologically organized by IPA and predicted to be down-regulated following knockdown of *LAPTM4B* in serum deprived conditions. Color indicates differential gene expression relative to cells transfected with control siRNA: red, up-regulated; green; down-regulated.

To gain additional insights into mechanisms of *LAPTM4B* oncogenic function and cell signaling, we sought to compare and contrast the transcriptome of cells transfected with scrambled siRNA and *LAPTM4B*-specific siRNA and cultured in the presence or absence of serum. We chose the Calu-6 cell line for the microarray experiment due to our observation noted above (Fig. 2) that this cell line displayed more pronounced cell growth inhibition following serum deprivation and apoptosis following *LAPTM4B* knockdown compared to the *KRAS* wild type H1650 cell line. Gene expression profiling, using the Affymetrix Human Gene 1.0 ST platform, identified 1,252 gene features in serum-starved Calu-6 cells and 1,669 gene features in cells cultured with 10% FBS that were significantly differentially expressed, based on a *P* < 0.01 threshold, by *LAPTM4B* knockdown (Fig. 3A, Supplementary Tables S3 and S4, respectively). To gain insights into functional gene expression programs downstream of *LAPTM4B*, we interrogated, using Ingenuity Pathways analysis (IPA), topological gene interaction networks that are differentially modulated following *LAPTM4B* knockdown in serum-starved cells compared to cells cultured in serum-containing medium. This analysis demonstrated that knockdown of *LAPTM4B* in serum starved cells molecularly mimicked effects of several anti-cancer agents including small molecular weight inhibitors of PI3K and MEK (LY294002 and U0126, respectively) (Fig. 3B). Moreover, the comparative pathways analysis predicted inhibition (indicated by a negative Z-score) by *LAPTM4B* knockdown of several regulators of gene expression (e.g. transcription factors) which was augmented in serum starved cells compared to cells cultured in serum (Fig. 3B). Notably, the analysis revealed that the *NRF2* transcription factor exhibited the lowest Z score and activated state following *LAPTM4B* knockdown in serum starved lung cancer cells (Fig. 3B). Further analysis also demonstrated that the *NRF2*-mediated stress response and pathway was the most down-regulated canonical pathway by *LAPTM4B* knockdown (P < 0.001) (Fig. 3C). In addition, a topologically organized gene network mediated by *NRF2* was found to be enriched with genes that were preferentially down-regulated by *LAPTM4B* knockdown in serum starved lung cancer cells including various anti-oxidant genes (e.g. *HMOX1*) (Fig. 3D). Our microarray transcriptome profiling and pathways analyses provide additional insights into the impact of *LAPTM4B* expression on oncogenic cellular signaling in lung cancer cells.

### *LAPTM4B* promotes expression of *NRF2* and downstream NRF2-target genes

**Figure 4:**
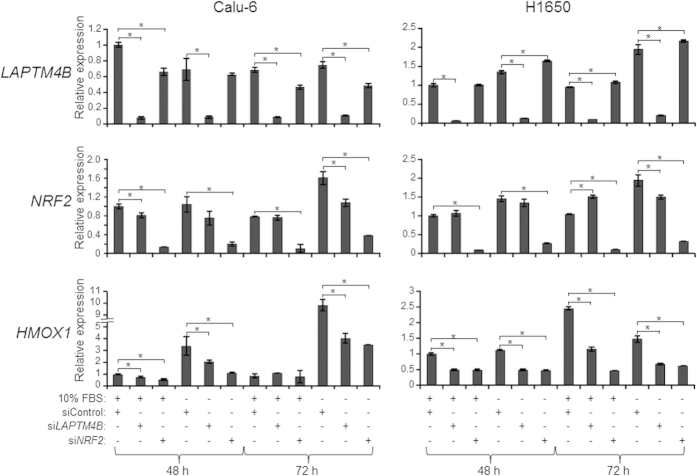
*LAPTM4B* up-regulates *NRF2* and *HMOX1* expression in serum-starved lung cancer cells. Calu-6 (left) and H1650 (right) lung cancer cells were transfected with control, *LAPTM4B*-specific or *NRF2*-targeting siRNAs. One day following transfection, cells were washed twice with 1x PBS and then incubated in cell culture medium containing 0% or 10% FBS for the indicated time points. Total RNA was isolated from all samples and analyzed for *LAPTM4B* (upper), *NRF2* (middle) and *HMOX1* (lower) expression levels by qRT-PCR at 48 h and 72 h following serum deprivation as detailed in the Materials and Methods section. Expression changes are depicted relative to the first sample (cells transfected with control siRNA and grown in medium containing 10% FBS for 48 h). qRT-PCR analysis was performed in triplicates for all samples. *indicate *P*-values < 0.05 assessed by the Student’s t-test.

We then determined to examine, by qRT-PCR, expression levels of *NRF2* following perturbation of *LAPTM4B* expression. We also sought to assess expression levels of *HMOX1*, an anti-oxidant heme oxygenase that is up-regulated by *NRF2* following cellular stress^29^. Our gene profiling and pathways analysis revealed that *HMOX1* was one of the most down-regulated genes in the *NRF2*-mediated gene interaction network following *LAPTM4B* knockdown (Fig. 3D). Calu-6 (left panel) and H1650 (right panel) cells that were transfected with *LAPTM4B*-specific siRNA exhibited significantly (*P* < 0.05) suppressed *NRF2* and *HMOX1* expression levels by 72 hours following serum starvation compared to cells transfected with control siRNA (Fig. 4). Conversely, cells transfected with *LAPTM4B* over-expression vector exhibited significantly (*P* < 0.05) increased *NRF2* and *HMOX1* levels by 72 hours following serum starvation relative to cells transfected with control vector (Supplementary Figure S2). *NRF2* levels were either unchanged, increased (Fig. 4) or even decreased (Supplementary Figure S2) in cells transfected with *LAPTM4B*-specific siRNA or *LAPTM4B* over-expression vectors, respectively, and cultured in medium containing 10% FBS. It is noteworthy that we also observed significant reduction in *HMOX1* levels following *LAPTM4B* knockdown in the absence of significantly reduced *NRF2* expression (Fig. 4, middle right and lower right panels) suggesting that *HMOX1* may be regulated by *LAPTM4B* in an *NRF2*-dependent and -independent manner. We also confirmed *HMOX1* regulation by *NRF2* evidenced by significant reduction in expression of the heme oxygenase following *NRF2* knockdown (Fig. 4, lower panels). To further probe the impact of *LAPTM4B* expression on the *NRF2* pathway, we examined in *KRAS* mutant Calu-6 cells and following *LAPTM4B* knockdown, the expression of additional downstream targets that were found in our functional pathways analysis of the microarray data (Fig. 3D). We observed by qRT-PCR analysis significantly suppressed expression of the *NRF2* target genes^30^,^31^ NAD(P)H dehydrogenase quinone 1 (*NQO1*), malic enzyme 1 (*ME1*) and solute carrier family 7 member 11 (*SLC7A11*) by 72 hours following *LAPTM4B* knockdown (Supplementary Figure S3). Additionally, we confirmed the suppression of these genes by RNA interference-mediated knockdown of *NRF2* (Supplementary Figure S3). Our findings suggest that *LAPTM4B* positively regulates the expression of *NRF2* and its anti-oxidant target gene *HMOX1*.

### *LAPTM4B* promotes nuclear localization of *NRF2*

**Figure 5:**
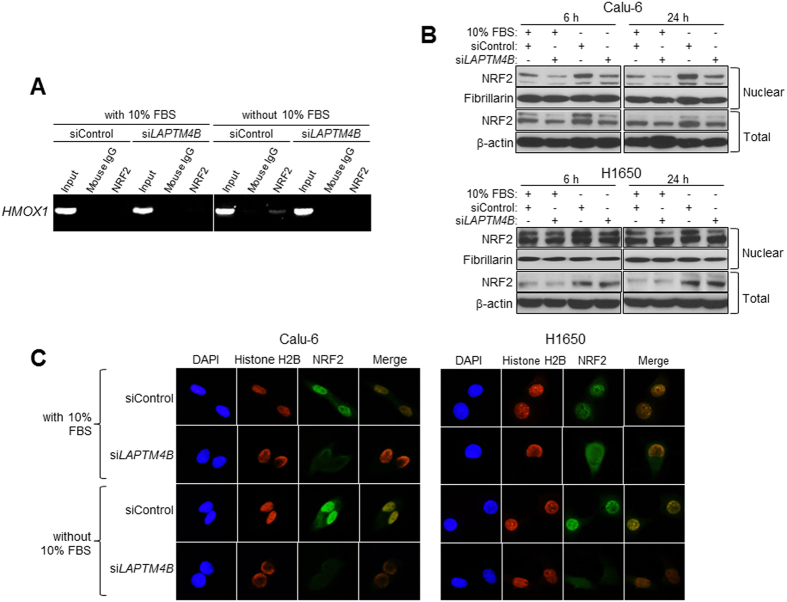
*LAPTM4B* promotes nuclear localization of the *NRF2* transcription factor. (**A**) Calu-6 lung cancer cells were transfected with control or *LAPTM4B*-specific siRNAs for 24 h after which cells were washed twice with 1x PBS and incubated in cell culture medium containing 0% or 10% FBS for 48 h. Samples were then fixed with 1% formaldehyde and subjected to ChIP analysis of *HMOX1* promoter bound to *NRF2* protein as detailed in the Materials and Methods section. Samples were analyzed on a 1% agarose gel. (**B**) Calu-6 (upper panel) and H1650 (lower panel) cells were transfected as in (**A**). One day following transfection, cells were incubated in culture medium containing 0% or 10% FBS for the indicated time points after which total and nuclear protein lysates were isolated and subjected to SDS-PAGE as detailed in the Materials and Methods section. Western blotting analysis was then performed for total and nuclear NRF2 protein levels. Membranes were stained with antibodies against Fibrillarin and *β*-Actin to ensure equal loading of nuclear and total proteins, respectively. (**C**) Calu-6 (left) and H1650 (right) lung cancer cells were transfected as in (**A**) and then incubated in medium containing 0% or 10% FBS for 6 hours. Cells were then fixed with 4% paraformaldehyde and analyzed by ICC for NRF2 and Histone H2B as detailed in the Materials and Methods section. All assays are representative of three independent experiments.

*NRF2* is transcription factor that upon activation (e.g. due to cellular stressors) localizes to the nucleus and transactivates the expression of various anti-oxidant genes including *HMOX1*^29^,^32^,^33^. Therefore, we sought to determine whether *LAPTM4B* promotes *NRF2*-mediated transactivation of *HMOX1* and intracellular localization of *NRF2* to the nucleus. Chromatin immunoprecipitation (ChIP) analysis demonstrated that Calu-6 cells transfected with control siRNA exhibited increased, albeit modest, NRF2 protein binding to *HMOX1* promoter following serum starvation relative to similarly transfected cells but cultured in medium containing 10% FBS (Fig. 5A). Serum starvation-induced binding of NRF2 protein to the *HMOX1* promoter was attenuated in cells transfected with *LAPTM4B*-specific siRNA compared to cells transfected with control siRNA (Fig. 5A). Moreover, western blotting analysis of nuclear and total protein lysates revealed that in control Calu-6 (upper panel) and H1650 (lower panel) lung cancer cells, serum starvation increased nuclear levels of NRF2 protein (Fig. 5B). This effect was attenuated in cells transfected with *LAPTM4B*-specific siRNA compared to control cells (Fig. 5B). Moreover and conversely, over-expression of *LAPTM4B* increased nuclear NRF2 protein levels (Supplementary Figure S4). In addition, immunocytochemical (ICC) analysis concordantly demonstrated that *LAPTM4B* knockdown in both *KRAS* mutant Calu-6 cells and *KRAS* wild type H1650 cells reduced nuclear localization of NRF2 protein evidenced by decreased co-localization with the nuclear marker Histone H2B. These findings suggest that, under cellular stress conditions such as serum starvation, *LAPTM4B* promotes nuclear localization and activation of *NRF2*.

## Discussion

Our recent efforts to understand early events in NSCLC pathogenesis, through interrogating the transcriptome of the field cancerization in the airway and lung, demonstrated that *LAPTM4B* is largely up-regulated in the airway “field” closest to NSCLCs compared to more distant epithelial fields^11^. This observation led us to hypothesize that *LAPTM4B* expression plays important roles in lung cancer cell growth. In the present study, we sought to characterize *LAPTM4B* expression in NSCLC specimens and its impact on lung cancer cell malignant phenotype and cell signaling *in vitro*. *LAPTM4B* expression, when analyzed by *in situ* hybridization in NSCLC tissues, was increased in smoker relative to non-smoker LUADs and was predictive of poor survival in LUADs and not in SCCs. We also found that *LAPTM4B* expression was important for survival and promotion of autophagy in lung cancer cells under serum starvation. Moreover, our gene expression profiling coupled with functional pathways analyses revealed that *LAPTM4B* promoted the *NRF2* stress response and pathway in lung cancer cells grown under serum deprived conditions. We further demonstrated that *LAPTM4B* was a positive regulator of various *NRF2* targets (*HMOX1*, *NQO1*, *ME1*, *SLC7A11*) and of *NRF2* itself as well as promoted the activation and nuclear localization of the *NRF2* transcription factor. Our study provides new insights into the oncogenic signaling function of *LAPTM4B* in lung cancer cells and its cross talk with other canonical pathways such as *NRF2/HMOX1*.

Earlier work has demonstrated that *LAPTM4B* is over-expressed in various malignancies including those of the liver, ovary, breast and prostate^16^,^17^,^18^,^34^. Moreover, Li *et al.* demonstrated that *LAPTM4B*, in the 8q22 locus, is amplified in breast tumors^21^. In the same study by Li *et al.* *LAPTM4B* over-expression by amplification was found to be associated with breast cancer recurrence^21^. It is worthwhile to mention that while our study was being completed, a recent report by Tang *et al.* demonstrated that *LAPTM4B* is associated with poor prognosis in NSCLC patients^35^. In our present study, we interrogated the expression of *LAPTM4B* mRNA in histological NSCLC specimens and noted that *LAPTM4B* was associated with survival in LUADs but not in SCCs and, thus, suggesting that this oncogene may serve as a prognostic marker in a specific histological subgroup and not in all NSCLC patients. It cannot be neglected that, in contrast to the study by Tang *et al.* we analyzed *LAPTM4B* mRNA by *in situ* hybridization which we found in our hands to yield more specific epithelial reactivity compared to analysis of LAPTM4B protein reactivity by immunohistochemical (IHC) methods. It is plausible to surmise that analysis of a relatively larger series of NSCLC specimens (n = 368) in our study allowed us to shed light on more specific associations of *LAPTM4B* expression with lung cancer prognosis. In addition, it is reasonable to suggest that our analysis of a relatively larger group of LUAD patients including non-smokers enabled us to identify significant correlations between *LAPTM4B* expression and smoking status. In addition and notably, our current observation on elevated *LAPTM4B* expression in tumors derived from smokers corroborates our notion and recent report that this putative oncogene is elevated in the smoking exposed airway field of cancerization^11^.

Our study corroborates earlier work on the role of *LAPTM4B* in promoting survival and autophagy during cellular stress^23^. It has been suggested that fusion of autophagosomes with lysosomes for subsequent formation of autolysosomes is crucial for proper functioning of autophagy and lysosome-dependent degradation that is necessary to maintain intracellular homeostasis^22^,^27^,^28^. We found that in lung cancer cells grown under serum deprived conditions, suppression of *LAPTM4B* expression caused an increase in LC3-II and p62 protein levels suggesting decreased autophagosome turnover and fusion with lysosomes as well as abnormal autophagic flux^27^,^28^. We also demonstrated that in serum deprived lung cancer cells, LAPTM4B protein indeed co-localized with LC3 suggestive of the implication of *LAPTM4B* in autophagic flux and autolysosome formation. Our findings insinuate that *LAPTM4B* is an important mediator of autophagy for survival of lung cancer cells during serum starvation. It is also plausible to propose that this role for *LAPTM4B* may extend to other cell stressors such as chemotherapy and that targeting the lysosomal degradation pathway may help augment the anti-cancer effects of chemotherapeutic regimens.

The transcription factor *NRF2* is a master regulator of intracellular homeostasis and oxidative stress^29^,^32^. Upon activation and translocation to the nucleus, *NRF2* was shown to transactivate the expression of genes, such as *HMOX1*, with antioxidant response elements (AREs) that can reduce oxidative and genotoxic stress induced by agents such as cigarette smoke or by proinflammatory cells such as macrophages and neutrophils^29^,^32^,^36^. These genes are thought to elicit important pro-survival effects in cancer cells as studies have shown that NRF2 promotes lung cancer cell proliferation and resistance to cisplatinum-based therapy^37^,^38^. It is worthwhile to mention that NSCLC next-generation sequencing (NGS) studies demonstrated that the *NRF2* pathway is aberrant in a significant fraction of NSCLCs, either by activating mutations in *NRF2* or by inactivating mutations or deletions in *NRF2* inhibitor and tumor suppressor Kelch-like ECH-associated protein 1 (*KEAP1*)^39^,^40^. Our gene expression profiling coupled with functional pathways analysis revealed that *LAPTM4B* expression promoted the *NRF2* stress response and pathway in lung cancer cells deprived of serum. Further analysis demonstrated that *LAPTM4B* also mediated NRF2 nuclear localization. It is plausible to surmise that *LAPTM4B*-mediated signaling may represent a novel mechanism of *NRF2* activation aside from genomic alterations in the *NRF2* axis itself. Future studies to further delineate the role of this signaling axis in lung cancer cell growth, for example by employing three-dimensional culture assays or using *in vivo* settings, are warranted. It is noteworthy that reports by Komatsu *et al.*^41^ and Ichimura *et al.*^42^ demonstrated that autophagy, through the p62 protein, can activate the NRF2 transcription factor. It is conceivable to speculate that autophagy may be implicated in activation of *NRF2* pathway by *LAPTM4B* since we observed autophagy induction as early as 2 hours following serum starvation.

It is worthwhile to mention that we found that *LAPTM4B* knockdown resulted in more pronounced apoptosis in Calu-6 cells which harbor *KRAS* mutation compared to H1650 cells which are *KRAS* wild type. We also noted that down-regulation of NRF2 and its cytoplasmic retention following *LAPTM4B* knockdown was more pronounced in *KRAS* mutant Calu-6 cells compared to *KRAS* wild type H1650 cells Additionally, we found that knockdown of *NRF2* either down-regulated (in *KRAS* mutant Calu-6 cells) or up-regulated (in *KRAS* wild type H1650 cells) *LAPTM4B* levels. It is plausible that *LAPTM4B-NRF2* signaling is modulated differentially in different cell lines with distinct mutational, genetic and epigenetic profiles; being more activated in *KRAS* mutant compared to wild type lung cancer cells. Our current findings along with previous published reports on aberrant *NRF2* activation suggest that *LAPTM4B/NRF2* signaling axis may represent a novel target for treatment of NSCLCs and particularly those with mutations in the *KRAS* oncogene.

In conclusion, we have shown in this study that *LAPTM4B* was up-regulated in smoker compared to non-smoker LUADs and was associated with poor prognosis in non-squamous NSCLCs. We also demonstrated that *LAPTM4B* promoted survival and autophagy as well as activated the *NRF2* stress response pathway in lung cancer cells, particularly those with mutations in the *KRAS* oncogene, under serum deprived conditions. Our study points to new mechanisms of *NRF2* activation as well as to targets, e.g. *LAPTM4B/NRF2* signaling axis, for development of new strategies for lung cancer therapy.

## Materials and Methods

The methods were carried out in accordance with the approved guidelines.

### NSCLC tissue microarrays

All human tissues were obtained from the MD Anderson Cancer Center Lung Cancer Specialized Program of Research Excellence (SPORE) tissue bank (Houston, TX) and had been classified using the 2004 World Health Organization classification system as described before^43^,^44^. Specimens were obtained under a protocol that was approved by the MD Anderson Cancer Center Institutional Review Board (IRB). Informed consent was obtained from all subjects. Detailed clinical and pathologic information was available for most of these cases and included patients’ demographic data, smoking history (never smokers or ever smokers, patients who had smoked at least 100 cigarettes in their lifetime), and pathologic tumor–node–metastasis (TNM) staging. The tissue microarrays (TMAs) analyzed in this study comprised 368 NSCLC tumor specimens (245 LUADs and 123 SCCs) (Supplementary Table S1). After histologic examination of NSCLC specimens, the NSCLC TMAs were constructed by obtaining three 1 mm diameter cores from each tumor at 3 different sites (periphery, intermediate, and central tumor sites). The TMAs were prepared with a manual tissue arrayer (Advanced Tissue Arrayer ATA100, Chemicon International) as described previously^43^,^44^.

### *LAPTM4B in situ* hybridization

*In situ* analysis of *LAPTM4B* messenger RNA (mRNA) in histological formalin fixed paraffin embedded (FFPE) specimens was performed using the QuantiGene 2.0 kit and QuantiGene View (QGV) RNA *in situ* hybridization (ISH) tissue assay from Affymetrix according to the manufacturer’s instructions. The assay comprised a singleplex probe for *LAPTM4B* based on the oncogene’s transcript reference sequence from the NCBI (NM_018407). FFPE pellets from lung cancer cells (Calu-6) with high basal expression of *LAPTM4B* and transfected with control and *LAPTM4B*-specific siRNA were used as positive and negative controls, respectively, for optimization of the assay. Samples and specimens processed similarly, except for the omission of the *LAPTM4B* probe were used as additional negative controls. *LAPTM4B* mRNA reactivity was examined by an experienced pathologist (J. Fujimoto) using a light microscope under a × 20 magnification objective. *LAPTM4B* mRNA expression was quantified using a 4-value intensity score (0, none; 1, weak; 2, moderate; and 3, strong) and the percentage (0%–100%) of the extent of reactivity. A final expression score (H-score) was obtained by multiplying the intensity and reactivity values (range, 0–300)^43^,^44^.

### Cell culture and reagents

The Calu-6 cell line was purchased from the American Type Culture collection (ATCC) and the H1650 cell line was obtained from Dr. Adi F. Gazdar (The University of Texas Southwestern Medical Center, Dallas, TX). The lung cancer cell lines were cultured in a 1:1 medium mix of Dulbecco’s Modified Eagle Medium (DMEM) and Ham’s F12 supplemented with 10% fetal bovine serum (FBS). Cells were maintained in humidified 5% CO_2_ incubator. Cell lines used in the study were authenticated by short tandem repeat DNA fingerprinting using the PowerPlex 16 HS System (Promega) and the STR profiles were verified with MD Anderson fingerprint database. For experiments requiring serum starvation, serum containing cell culture media were removed, cells were then gently washed twice with 1x phosphate buffered saline (PBS) and then incubated in serum-free cell culture medium overnight. For analysis of autophagic flux, cells were cultured in serum-free medium containing Pepstatin A and EST (EMD Millipore).

### Total RNA isolation

Total RNA was isolated from cells using the RNeasy kit from Qiagen according to the manufacturer’s instructions. RNA was quantified using the Nanodrop 1000 spectrophotometer (Thermo Scientific). RNA quality was assessed based on RNA integrity numbers generated by the Agilent Bioanalyzer 2000 (Agilent) according to the manufacturer’s instructions.

### Microarray processing and analysis

RNA was also isolated from Calu-6 cells transfected with scrambled siRNA and *LAPTM4B*-specific siRNA and that were cultured in medium with and without 10% FBS (3 replicates each, *n* = 12). RNA samples were analyzed by microarray expression profiling using the Affymetrix Human Gene 1.0 ST platform (Affymetrix) according to the manufacturer’s instructions and as described previously^43^. Microarray data were submitted to the Gene Expression Omnibus under series GSE66606 (samples GSM1626103 to GSM1626114) and were MIAME compliant. Raw data were normalized using Robust Multichip Array and log2 transformed using BRB-ArrayTools v 4.3.0 developed by Richard Simon and the BRB-ArrayTools Development Team (Biometric Research Branch)^45^. Genes significantly differentially expressed between Calu-6 lung cancer cells transfected with *LAPTM4B*-specific siRNA compared with cells transfected with scrambled siRNA were selected on the basis of a *P* < 0.01^46^. Two analyses were performed side by side to determine effect of *LAPTM4B* on gene expression in cells with and without serum starvation (*n* = 1,252 and 1,669 transcripts, respectively; Supplementary Tables S3 and S4). Functional pathway analysis was conducted using the commercially available software Ingenuity Pathways Analysis (IPA) according to the manufacturer’s instructions.

### Quantitative real-time PCR (qRT-PCR)

A total of 500 ng to 1µg of RNA was reverse-transcribed using the High Capacity RNA-to-cDNA kit (Life Technologies) according to the manufacturer’s instructions and diluted in nuclease-free water. qRT-PCR was conducted using predesigned TaqMan expression assays for *LAPTM4B* (Hs00363282_m1), *NRF2/NFE2L2* (Hs00975961_g1), *HMOX1* (Hs01110250_m1), *NQO1* (Hs00168547_m1), *SLC7A11* (Hs00921938_m1), *ME1* (Hs00159110_m1) and *β-ACTIN* (Hs99999903_m1) (all Life Technologies) on an ABI 7900HT Fast Real-Time PCR System (Applied Biosystems) according to the manufacturer’s instructions. Reactions were initially carried out at 50 °C for 2 minutes and 95 °C for 10 minutes, followed by 40 cycles at 95 °C for 15 seconds and 60 °C for 1 minute. All samples were run in triplicates and normalized using *β-ACTIN* expression values. Quantification of relative expression was calculated using the comparative threshold cycle (CT) and 2^−ΔΔCT^ relative quantification method.

### Transfection of siRNA and expression vectors

Small interfering RNAs (siRNAs) against *LAPTM4B* and *NRF2* as well as scrambled (control) siRNA were synthesized by a proprietary design as SMARTpool siRNA (Dharmacon, Thermoscientific). Knockdown of *LAPTM4B* or *NRF2* expression was conducted using Lipofectamine RNAiMAX (Life Technologies) according to the manufacturer’s instructions. *LAPTM4B* overexpression was achieved using a C-terminal FLAG-tagged *LAPTM4B* cDNA clone inserted into a cytomegalovirus plasmid pCMV6 vector (Origene). Cells were transfected with the expression vectors using Effectene Transfection Reagent (Qiagen) according to the manufacturer’s instructions. For experiments requiring serum starvation, transfections were conducted one day before overnight incubation with serum-free cell culture medium.

### Trypan blue exclusion and cell count

Cells were seeded in triplicate in 12-well plates and then transfected the following day with siRNAs or overexpression vectors. One day following transfection, cells were incubated overnight in medium with or without 10% FBS and after 24, 48 and 72 hours after the medium change cells were washed twice with 1x PBS, trypsinized, mixed with 0.4% Trypan blue solution (sigma Aldrich) and then counted using the Reichert Bright-Line Hemacytometer (Hausser Scientific) by the Trypan blue exclusion principle.

### Western blot analysis

For extraction of total protein, cell monolayers were washed twice with PBS, harvested, and lysed with ice-cold radioimmunoprecipitation assay (RIPA) buffer (Sigma-Aldrich). Nuclear proteins were extracted using NE-PER nuclear and cytoplasmic extraction reagents (Thermo scientific) according to the manufacturer’s instructions. Protein lysates (20 µg) were then subjected to SDS- PAGE and Western blotting. Primary antibodies used for immunoblotting include those raised against NRF2, p62/SQSTM1, LC3A/B and PARP (all from Cell Signaling Technology) as well against LAPTM4B (Santa Cruz Technology). Antibodies against Fibrillarin (Cell Signaling Technology) and β-actin (Sigma Aldrich) were used as loading controls. The primary antibodies were diluted in 5% BSA (1:10,000 for β-actin and 1:1,000 for all other antibodies). Antibody binding was detected by enhanced chemiluminescence using the SuperSignal West Pico chemiluminescent substrate (Thermo Scientific).

### Immunocytochemistry

Cells were seeded at a density of 5 × 10^5^ cells per well in six well plates containing cover glasses/slips in each well. Following indicated transfections, serum starvation and time points, cells were fixed in 4% paraformaldehyde (Electron Microscopy Sciences) and then permeabilized in pre-chilled 100% methanol for 10 minutes at −20 °C. Cells were then washed with 1x PBS for 5 minutes, blocked and incubated overnight at 4 °C with antibodies raised against LC3A/B antibody (Cell Signaling Technology), FLAG tag (Cell Signaling Technology), NRF2 (Novus Biologicals) and Histone H2B (Abcam). Conjugated secondary antibodies used include goat anti-mouse and anti-rabbit IgG (H + L) Fluorescein (FITC) (Jackson ImmunoResearch Labs) as well as anti-rabbit and anti-mouse IgG Alexa Fluor® 555 conjugates (Cell Signaling Technology). Fixed cells were incubated with diluted secondary antibodies for 1 hour at room temperature after which they were washed three times with 1x PBS and mounted with ProLong Gold Antifade Reagent (Cell Signaling Technology). Analysis was carried out in an epifluorescence microscope using single interference filters set for green (fluorescein isothiocyanate, FITC), red (Texas Red), and blue (DAPI) as well as dual (red/green) band pass filters.

### Chromatin immunoprecipitation (ChIP)

Cells were initially plated at a seeding density of 1 × 10^6^ in 10 cm dishes. Following indicated transfections and time points, ChIP was conducted using the EZ-ChIP™ - Chromatin Immunoprecipitation Kit (Millipore) according to the manufacturer’s instructions and using a primary antibody raised against NRF2 (1:100 dilution, Cell signaling Technology). PCR amplification of the bound *HMOX1* promoter was performed using specific primers (forward: TTCCTTCTTGCTAATGATTTACTGTCTT, reverse: ACCAACCGACAAAAGTCAGGTT). Amplification reactions were carried using 34 cycles at 94 °C for 15 seconds followed by 58 °C for 30 seconds and 72 °C for 30 seconds.

### Statistical analysis

ANOVA and Student’s t-test were utilized to test for statistical significance among different groups in the *in vitro* experiments. Statistical analysis of the ISH data was first summarized using standard descriptive statistics and frequency tabulations. Association of *LAPTM4B* mRNA with patient outcome was estimated using the Kaplan-Meier method and compared among groups by log-rank statistical tests. All computations were carried out in STATISTICA (StatSoft) and in the R language environment (www.R-project.org).

## Additional Information

**How to cite this article**: Maki, Y. *et al.* LAPTM4B is associated with poor prognosis in NSCLC and promotes the NRF2-mediated stress response pathway in lung cancer cells. *Sci. Rep.* **5**, 13846; doi: 10.1038/srep13846 (2015).

## Supplementary Material

Supplementary Information
