# Supplementary material for: LAPTM4B is associated with poor prognosis in NSCLC and promotes the NRF2-mediated stress response pathway in lung cancer cells
Source: Sci Rep. 2015 Sep 7;5:13846. doi: 10.1038/srep13846 (PMC4561374; doi:10.1038/srep13846)
Supplement: Supplementary Information [file srep13846-s1.pdf]

LAPTM4B is associated with poor prognosis in NSCLC and promotes the NRF2-mediated stress response pathway in lung cancer cells

Yuho Maki, Junya Fujimoto, Wenhua Lang, Li Xu, Carmen Behrens, Ignacio I. Wistuba, Humam Kadara**.**

Supplementary figure legends

Supplementary Figure 1. Western blotting of LAPTM4B protein following RNA interference-mediated knockdown. Calu-6 lung cancer cells were transfected with control or *LAPTM4B*-specific siRNAs and protein lysates were isolated and subjected to SDS-PAGE as detailed in the Materials and Methods section. Western blotting analysis was then performed for LAPTM4B protein. Membranes were stained with antibody against β-Actin to ensure equal loading of proteins.

Supplementary Figure 2. *LAPTM4B* over-expression increases *NRF2* and *HMOX1* mRNA levels. Calu-6 lung cancer cells were transfected with control or *LAPTM4B* over-expression vectors. One day following transfection, cells were washed twice with 1x PBS and then incubated in cell culture medium containing 0% or 10% FBS for the indicated time points. Total RNA was isolated from all samples and analyzed for *LAPTM4B* (upper), *NRF2* (middle) and *HMOX1* (lower) expression levels by qRT-PCR as detailed in the Materials and Methods section. Expression changes are depicted relative to the first sample (cells transfected with control vector and grown in medium containing 10% FBS for 48 h). qRT-PCR analysis was performed in triplicates for all samples. * indicate *P*-values < 0.05 assessed by the Student's t-test.

Supplementary Figure 3. *LAPTM4B* knockdown suppresses expression of *NRF2* target genes. Calu-6 lung cancer cells were transfected with control, *LAPTM4B*-specific or *NRF2*-targeting siRNAs. One day following transfection, cells were washed twice with 1x PBS and then incubated in cell culture medium containing 0% FBS for the indicated time points. Total RNA was isolated from all samples and analyzed for *SLC7A11* (upper), *ME1* (middle) and *NQO1* (lower) expression levels by qRT-PCR as detailed in the Materials and Methods section. Expression changes are depicted relative to the first sample (cells transfected with control siRNA. qRT-PCR analysis was performed in triplicates for all samples. * indicate P-values < 0.05 assessed by the Student's t-test.

Supplementary Figure 4. *LAPTM4B* over-expression increases NRF2 nuclear localization. Calu-6 lung cancer cells were transfected with control or *LAPTM4B* over-expressing vectors. One day following transfection, cells were incubated in culture medium containing 0% or 10% FBS for the indicated time points after which total and nuclear protein lysates were isolated and subjected to SDS-PAGE as detailed in the Materials and Methods section. Western blotting analysis was then performed for total and nuclear NRF2 protein levels. Membranes were stained with antibodies against Fibrillarin and β-Actin to ensure equal loading of nuclear and total proteins, respectively.

Supplementary figure 1

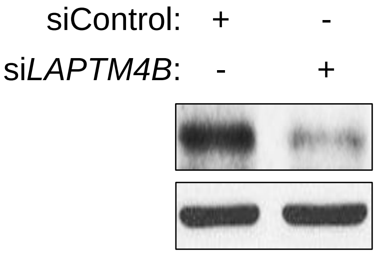

Supplementary figure 2

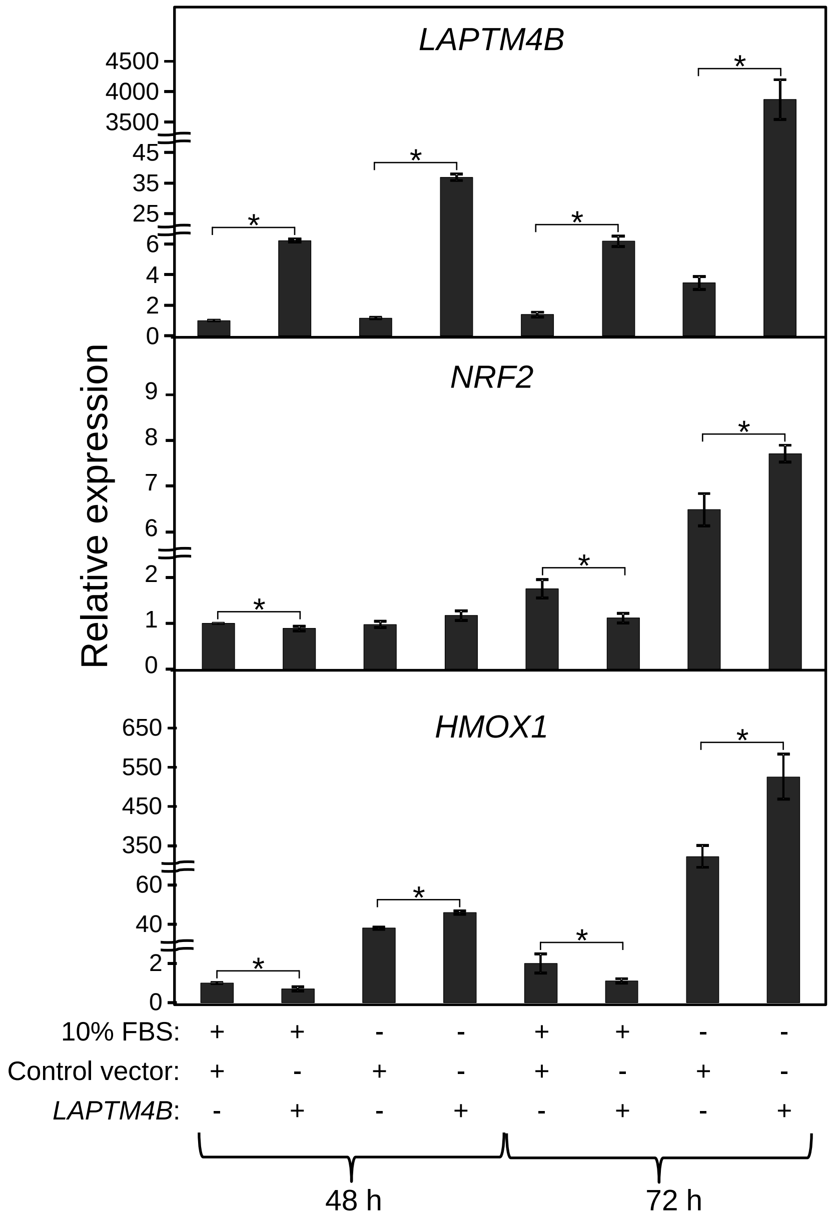

Supplementary figure 3

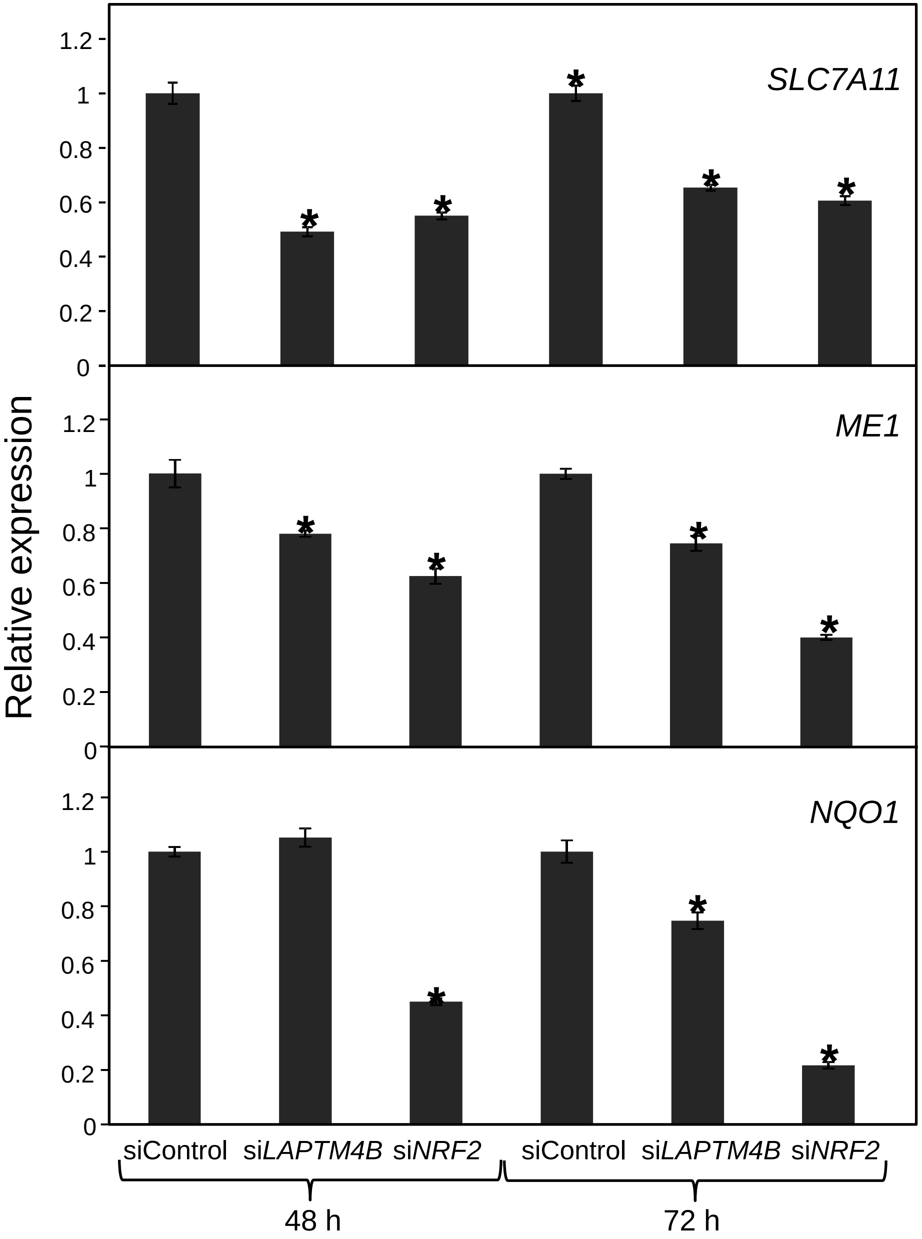

Supplementary figure 4

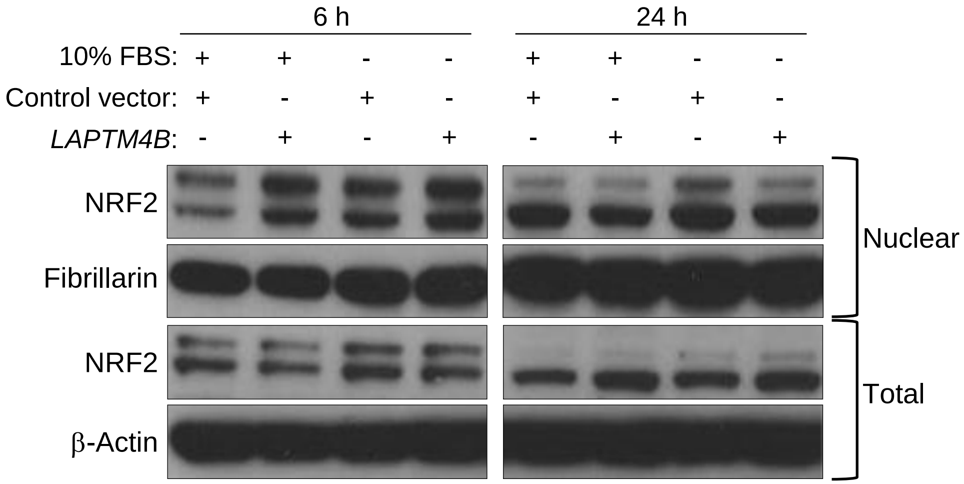

Supplementary Table 1. Clinicopathological information of the NSCLC tissue microarray specimens analyzed by *in situ* hybridization

Covariate	Covariate categories	Frequency (percentage)
Race	African American	17 (4.6%)
	Asian	5 (1.4%)
	Hispanic	10 (2.7%)
	Caucasian	336 (91.3%)
Gender	Male	174 (47.3%)
	Female	194 (52.7%)
Tobacco history	No	43 (11.7%)
	Yes	325 (88.3%)
Smoking status	Never	43 (11.7%)
	Former	173 (47.0%)
	Current	152 (41.3%)
Histology	Adenocarcinoma	245 (66.6%)
	Squamous cell carcinoma	123 (33.4%)
Grade	Well	45 (12.2%)
	Moderate	200 (54.4%)
	Poor	123 (33.4%)
Stage	I	224 (60.9%)
	II	63 (17.1%)
	III	66 (17.9%)
	IV	15 (4.1%)

Supplementary Table 2. Mean *LAPTM4B in situ* hybridization score in lung adenocarcinomas and squamous cell carcinomas

**Histology**	**Mean ISH score**
LUAD	22.8
SCC	18.4

ISH, *in situ* hybridization; LUAD, lung adenocarcinoma; SCC, squamous cell carcinoma

Supplementary Table 3. Gene features significantly differentially expressed in serum starved lung cancer cells transfected with *LAPTM4B* -specific siRNA compared to cells transfected with control siRNA

Up-regulated gene features following siRNA-mediated *LAPTM4B* knockdown

Probeset	Accession	Symbol	p-value
7938608	NM_006108	SPON1	0.0000073
8115490	NM_033274	ADAM19	0.000014
8063942	NM_002531	NTSR1	0.0000155
7988260	NM_032892	FRMD5	0.0000175
8128991	NM_001105206	LAMA4	0.0000208
8116980	NM_001165032	RNF182	0.0000212
7896460			0.0000223
8129458	NM_033515	ARHGAP18	0.0000253
7961166	NM_013431	KLRC4	0.0000271
7959856	NM_004764	PIWIL1	0.0000302
7916112	NM_002867	RAB3B	0.0000444
8051949	NM_016932	SIX2	0.0000543
8163637	NM_002160	TNC	0.0000574
7991762	NM_000517	HBA2	0.0000643
7991766	NM_000558	HBA1	0.0000643
7947147	NM_148893	SVIP	0.0000684
7893668			0.0000693
7919326	NM_016361	ACP6	0.0000745
7975238	NM_020715	PLEKHH1	0.0000795
7928429	NM_002658	PLAU	0.0000826
8088264	NM_017563	IL17RD	0.0000865
8029489	NM_005581	BCAM	0.000118
8084165	NM_003106	SOX2	0.0001349
7908041	NM_002293	LAMC1	0.0001349
8057990	NM_153697	ANKRD44	0.0001398
7932453	NM_006393	NEBL	0.0001434
7944023	NM_182495	FAM55B	0.0001616
7970954	NM_004734	DCLK1	0.0001622
7895181			0.0001684
8130176	NM_024518	ULBP3	0.0001833
8156116	NM_001001551	C9orf103	0.0001859
7946569	NM_016422	RNF141	0.0001883
8059361	NM_020830	WDFY1	0.0001976
8065071	NM_198391	FLRT3	0.0002156
7892881			0.0002193
8140650	NM_012431	SEMA3E	0.0002374
8093053	NM_003234	TFRC	0.0002862
7945377	NM_001135053	SIGIRR	0.0003123
8007100	NM_001552	IGFBP4	0.0003198
7973974	NM_006194	PAX9	0.0003251
8147777	NM_138455	CTHRC1	0.0003306
8105411	NM_139017	IL31RA	0.0003344
8002975	NM_152342	CDYL2	0.0003495
7981538	NM_002226	JAG2	0.0003568
7909561	NR_026761	LINC00467	0.0003784
7951614	NM_002716	PPP2R1B	0.0003963
8174322	NM_024657	MORC4	0.0004148
7938834	NM_182964	NAV2	0.00044

8051197	NM_024584	CCDC121	0.0004513
7894652			0.000475
7905220	NM_004425	ECM1	0.0004801
8009502	NM_000891	KCNJ2	0.000507
8116780	NM_004415	DSP	0.0005238
7992877	NM_153028	ZNF75A	0.0005243
7904158	NM_020190	OLFML3	0.0005301
8083092	NM_001080412	ZBTB38	0.0005324
7939215	NM_012194	C11orf41	0.0005505
7977409	NM_001311	CRIP1	0.0005538
7894246			0.0005976
7907462	ENST00000363804		0.0006296
8109712	NM_001142556	HMMR	0.0006303
7913450	NM_005529	HSPG2	0.0006513
8107706	NM_005573	LMNB1	0.0006541
8080714	NM_001164317	FLNB	0.0006562
7896618			0.0006785
8067409	NM_005560	LAMA5	0.000684
8122202	NM_001130173	MYB	0.0007154
8111772	NM_001343	DAB2	0.0007448
7954065	NM_003979	GPRC5A	0.0007482
7952914	NM_032358	CCDC77	0.000769
8114920	NM_001387	DPYSL3	0.0007844
7893645			0.0007857
8005245	ENST00000411335		0.0008268
7922474	NM_014656	KIAA0040	0.0008297
8122637	NM_015278	SASH1	0.0008307
7961175	NM_002261	KLRC3	0.0008701
7974576	NM_001011713	NAA30	0.0008796
8174313	NM_024657	MORC4	0.0008819
8093826	NM_000683	ADRA2C	0.000886
7979241	NM_001202	BMP4	0.0009148
8089329	NM_014981	MYH15	0.0009393
8038029	NM_014959	CARD8	0.0009521
7928291	NM_004273	CHST3	0.00096
7992219	NM_003933	BAIAP3	0.0010335
8150138	NM_031271	TEX15	0.0010344
7917037	NM_001130042	CRYZ	0.001047
7911941	NM_015557	CHD5	0.001074
7939197	NM_005734	HIPK3	0.0011367
8157800	NR_029611	MIR181A2	0.001138
7895366			0.0012253
8124455	ENST00000362419		0.0012444
8083090	NM_001080412	ZBTB38	0.0012813
8054479	NM_005434	MALL	0.0013194
8040113	NM_003887	ASAP2	0.0013533
8125687	NM_004761	RGL2	0.0013628
7998297	NM_153350	FBXL16	0.0013668
8152053	NM_198401	ANKRD46	0.0013763
7974900	AK125380	FLJ43390	0.0013932
7961187	NM_213658	KLRC1	0.0014095
8130211	NM_182961	SYNE1	0.0014413
8066822	NM_018837	SULF2	0.0014519
8149551	NM_015310	PSD3	0.0014666
8099388	NM_005114	HS3ST1	0.0014872

8081941	ENST00000411139		0.0014965
8168461	ENST00000364577		0.001532
8176284	ENST00000364577		0.001532
8110237	NM_133369	UNC5A	0.0015396
7904761	NM_003637	ITGA10	0.0015874
8046380	NM_000210	ITGA6	0.0015908
7955317	NM_020039	ACCN2	0.0017097
8115410	NM_015465	GEMIN5	0.0017322
7961151	NM_007360	KLRK1	0.0017461
8178955	NM_004761	RGL2	0.0017523
8180144	NM_004761	RGL2	0.0017523
8008627	NM_005450	NOG	0.0017672
8151890	NM_033285	TP53INP1	0.0018042
8078805	NM_001106	ACVR2B	0.0018248
8148501	NM_032611	PTP4A3	0.0018791
8168674	ENST00000435214		0.001883
8133018	NM_001159279	ZNF716	0.0018899
8035939	ENST00000410539		0.0018956
7960865	NM_006931	SLC2A3	0.0019013
7931346	ENST00000365617		0.0019122
7905028	NM_002339	LSP1	0.0019122
8016473	NM_024016	HOXB8	0.0019433
8038347	NM_003598	TEAD2	0.0019549
7899703	NM_175852	TXLNA	0.0019672
7939237	NM_012194	C11orf41	0.0019674
7894470			0.0020673
8029098	NM_002483	CEACAM6	0.0020909
8085946	NM_005442	EOMES	0.0020909
8066260	NR_003017	SNORA71C	0.0021645
8131379	ENST00000384488		0.0021968
8039362	NM_178837	C19orf51	0.0023122
7945420	NM_002939	RNH1	0.0023437
7896343			0.0023561
8096004	NM_198892	BMP2K	0.0023692
8071927	NM_005265	GGT1	0.0023718
8133442	NM_032464	LAT2	0.0023726
8084818	NM_178335	CCDC50	0.0024237
8074168	ENST00000400192		0.0024254
8127051	NM_012288	TRAM2	0.0024257
7927874	ENST00000384707		0.0024276
8000974	NM_024706	ZNF668	0.0024276
7986092	NM_002569	FURIN	0.0024339
8141625	NM_004444	EPHB4	0.0024429
7923991	NM_025179	PLXNA2	0.0024549
8068633	NM_033171	B3GALT5	0.002465
8039672	BC032569		0.002466
8078397	NM_178868	CMTM8	0.0024672
7984014	ENST00000391323		0.0024882
8155946	NM_033305	VPS13A	0.0024921
7977452	ENST00000400192		0.0025532
8173549	ENST00000410878		0.0025644
7935361	NM_003061	SLIT1	0.0026141
8082846	NM_004441	EPHB1	0.0026722
7971590	NM_030925	CAB39L	0.0026782
7935403	NM_032900	ARHGAP19	0.0026899

7993588	NM_024847	TMC7	0.0027052
7950473	NM_004041	ARRB1	0.002724
8153002	NM_001135242	NDRG1	0.0027318
8033813	NM_017703	FBXL12	0.0027463
8024323	NM_138393	REEP6	0.0027638
8176253	AK098235		0.0027772
8104461			0.0027821
8171883	NR_002784	SMEK3P	0.0027961
7895296			0.0028074
8142194	NM_002291	LAMB1	0.0028284
8018343	NM_020679	MIF4GD	0.0028778
7893074			0.0028825
7976055			0.0029738
8052139			0.0029968
8149825	NM_003155	STC1	0.0030225
7987892	NM_022473	ZFP106	0.0030466
8035980	NM_033103	RHPN2	0.0030768
8072817	NM_152243	CDC42EP1	0.0031592
8043500			0.0033227
8152764	NM_014751	MTSS1	0.0033406
7956867	NM_003483	HMGA2	0.0033422
7893683			0.0033451
7979378	NM_018168	C14orf105	0.0033679
8162825			0.0033752
7897089	NM_014638	PLCH2	0.0034027
8010770	NM_001042422	SLC16A3	0.0034156
8073088	NM_021822	APOBEC3G	0.003446
7895449			0.0034671
8055171			0.0035125
8107970	NM_002188	IL13	0.0035341
7894240			0.0035389
7953040	NM_199460	CACNA1C	0.0035419
8080226	NM_015512	DNAH1	0.0035672
8161642			0.0035744
8130660	ENST00000410267		0.003587
7972748	ENST00000410652		0.0036397
7965800	NM_001177949	SYCP3	0.0036465
8035884	ENST00000410916		0.0036573
8103834	NM_000027	AGA	0.0036601
7895048			0.0036975
7911085	NM_198076	FAM36A	0.0036995
7907010	ENST00000434461		0.0037027
8062041	NM_018677	ACSS2	0.0037705
7963567	NM_002273	KRT8	0.0038361
7972650	NM_175929	FGF14	0.0038543
8168984	NM_001142524	BHLHB9	0.0038562
7909400	NM_002389	CD46	0.003864
7949172	NM_004579	MAP4K2	0.0038655
7993257	ENST00000362798		0.003881
8148615	NM_030895	ZNF696	0.0039298
8010499	NM_173627	ENDOV	0.0039385
8097813	NM_001025595	ARFIP1	0.0039413
7894676			0.0039666
8034334	NM_021143	ZNF20	0.0039763
8105229	NM_015946	PELO	0.003982

8110090	NM_022754	SFXN1	0.0039827
8170280	ENST00000364130		0.004005
8146579	NM_017780	CHD7	0.0040194
7892851			0.0040351
8024676	NM_133261	GIPC3	0.0040673
7893900			0.0040782
7928069	NM_001130103	COL13A1	0.0041568
8089082	NM_080927	DCBLD2	0.0041687
8113790	NM_178450	3-Mar	0.0041717
7946142	NM_145040	PRKCDBP	0.0041983
7927876	NM_030625	TET1	0.0042034
7932495	NM_001010911	C10orf114	0.0042413
8085370	ENST00000434751		0.0042417
7943263	NM_130847	AMOTL1	0.004246
7971996	ENST00000362511		0.0042705
8000957	NR_024348	FBXL19-AS1	0.0042789
7947528	NM_014186	COMMD9	0.0043605
7937483	NR_002585	SNORA52	0.0043606
8161418	NM_015234	GPR116	0.0044253
7894940			0.0044442
8106660	NM_006909	RASGRF2	0.0045887
8106771	NR_003719	NBPF22P	0.0046046
7934145	NM_207119	LRRC20	0.0046434
7915347	NM_133467	CITED4	0.0046438
7912706	NM_004431	EPHA2	0.0046539
8101260	NM_058172	ANTXR2	0.0046798
7894438			0.0047158
8151496	NM_001033723	ZNF704	0.0047304
7901788	NM_001134673	NFIA	0.0047922
8128327	ENST00000410130		0.0047941
7895834			0.0048238
8113130	NM_024717	MCTP1	0.0048338
8164810	NM_006266	RALGDS	0.0048542
8029688	NM_012099	CD3EAP	0.004871
7914270	NM_006762	LAPTM5	0.0048771
7892678			0.0049091
7914592	NM_033504	TMEM54	0.0049138
7959827	NM_001136103	TMEM132C	0.0049158
7928171	NM_003901	SGPL1	0.0049345
8112202	NM_006622	PLK2	0.0049457
7912361	NM_006610	MASP2	0.004986
8171148	NM_001669	ARSD	0.0050565
7944970	ENST00000410912		0.0051444
8092000	NR_001566	TERC	0.0051566
8086729	NM_182902	KIF9	0.0051925
8039533	NM_176820	NLRP9	0.00522
8009721	NM_014603	CDR2L	0.0052243
8026300	NM_078481	CD97	0.0052301
7903507	NM_001010883	FAM102B	0.0053337
8143108	ENST00000391001		0.0053473
8174281	ENST00000451146		0.00543
7896596			0.0054372
8058552	NM_005896	IDH1	0.0054384
8052089	ENST00000391278		0.005478
7922268	NM_014970	KIFAP3	0.0054798

8130370			0.0055148
8016891	NM_007146	VEZF1	0.005524
8095080	NM_006206	PDGFRA	0.0055244
8152962	NM_012472	LRRC6	0.005536
7918235	NM_152763	AKNAD1	0.0055458
8044933	NM_005270	GLI2	0.0055714
7976496	NM_001085	SERPINA3	0.0056479
8092177	NM_001146276	NCEH1	0.0056558
8002102	AF090894		0.0056953
8153430	NM_145201	NAPRT1	0.0057036
7951271	NM_002421	MMP1	0.0057338
8178590	NM_013974	DDAH2	0.0057578
8179819	NM_013974	DDAH2	0.0057578
8124057	ENST00000439284		0.0057663
7901229	NM_001441	FAAH	0.0057822
8012841			0.0058029
7924956	NM_012089	ABCB10	0.005812
8074316	NR_003267	GGT3P	0.0058189
7968911	ENST00000410943		0.0058444
8155510	NR_024060	FAM27A	0.0058459
8067680	NM_001037335	PRIC285	0.0058559
7984922	NM_002435	MPI	0.005866
7909503	NM_019605	SERTAD4	0.0059411
8111941	NM_001098272	HMGCS1	0.0059758
8145770			0.0059895
7894113			0.0060067
7983867	NM_032866	CGNL1	0.0060067
7997504	NM_001257	CDH13	0.0060358
8049007	NM_139073	SPATA3	0.0061127
8099982	NM_004307	APBB2	0.0061234
8049487	NM_024101	MLPH	0.0061248
8076757	NM_014246	CELSR1	0.0061634
8013509	NM_152914	C17orf103	0.006168
8114991	NM_024577	SH3TC2	0.0061843
7894038			0.006198
7923850	NM_052934	SLC26A9	0.006242
7910188	ENST00000365394		0.0062524
8000244	NM_020718	USP31	0.0062989
8032312	NM_138813	ATP8B3	0.0063159
7975876	NM_004452	ESRRB	0.0063866
7972487	NM_015296	DOCK9	0.0064765
8155497	NR_027421	FAM27C	0.0064965
8091411	NM_014220	TM4SF1	0.0066112
7894257			0.0066864
8161503	NR_024060	FAM27A	0.0067026
8151457	NM_012258	HEY1	0.006738
8138689	NM_003930	SKAP2	0.0067439
8164808	AK098728		0.0067789
8034202	NM_004283	RAB3D	0.0068285
8034097	NR_024333	LOC147727	0.0068664
7932214	NM_001039844	ACBD7	0.0068808
7893066			0.0068929
7985117	ENST00000449095		0.0068942
7977397	NM_001312	CRIP2	0.0068983
8003087	ENST00000362480		0.0069892

8107356	NM_152624	DCP2	0.0070207
7923753	NM_030952	NUAK2	0.0070685
7964499	NM_001122772	AGAP2	0.0071142
7994152	NM_006539	CACNG3	0.0071209
7892893			0.0071615
7896822	NM_198576	AGRN	0.007213
7900340	NM_181809	BMP8A	0.0072196
8024111	NM_004368	CNN2	0.0072344
7900009	NM_017629	EIF2C4	0.0072885
7963366	NM_002283	KRT85	0.0072909
7896110			0.0072948
8145225			0.0072958
8157610	NM_032552	DAB2IP	0.0073215
8130151	NM_139165	RAET1E	0.0073228
7895135			0.0073329
8094226	NM_001145191	FAM200B	0.0073473
8085060	ENST00000411108		0.0073695
7960874	NM_004054	C3AR1	0.0074148
8078971	NM_001248	ENTPD3	0.0075375
8049610	NM_198582	KLHL30	0.0075898
8060949	NM_022096	ANKRD5	0.0075948
7915949	AK090467	LOC388630	0.0076162
7896720			0.0076283
8172043	NM_006307	SRPX	0.0076387
8029147	NM_199002	ARHGEF1	0.0076543
8122840	NM_001122741	ESR1	0.0077104
8021418	NM_006785	MALT1	0.0077265
8070789	NM_198698	KRTAP12-4	0.0077354
8113504	NM_004772	C5orf13	0.0077417
7912672	NR_024279	FLJ37453	0.0077768
8166509	NM_001136233	FAM48B2	0.007827
8171844	NM_001136233	FAM48B2	0.007827
8158380	NM_013355	PKN3	0.0078612
8112342	NM_197941	ADAMTS6	0.0078685
8113403	NM_017676	GIN1	0.0078871
8126119	ENST00000410172		0.0078984
7950086	NM_006185	NUMA1	0.0079141
8007363	NM_032387	WNK4	0.0079861
8163678	NM_198186	ASTN2	0.0079862
8092594			0.0080311
7893165			0.0081237
8140424	NM_175064	SPDYE1	0.0081527
7997332	NM_001105663	NUDT7	0.008155
8125048	NM_013974	DDAH2	0.0081565
8146427	NM_052937	PCMTD1	0.0082002
7904693	ENST00000448309		0.0082673
8124484	NM_021058	HIST1H2BJ	0.0082688
8055711	NM_001164507	NEB	0.0083145
8035813	NM_003423	ZNF43	0.0083601
7952601	NM_001143820	ETS1	0.0084818
7893822			0.008514
8063923	NM_016354	SLCO4A1	0.008551
8166661	ENST00000327781		0.0085688
8073062	NM_004900	APOBEC3B	0.0085863
8062461	NM_004139	LBP	0.0086323

7984152	NM_203373	FBXL22	0.0086364
7896077			0.0086911
8010804	NM_001038618	NARF	0.0086941
7979615	NM_001024858	SPTB	0.008709
8151238	ENST00000410974		0.0087164
7927799	NM_001001330	REEP3	0.0087174
7992147	NM_021098	CACNA1H	0.0087317
7894706			0.0088182
7896523			0.0088393
8053364			0.0088404
8003875	NM_001124758	SPNS2	0.0089115
7895011			0.0089179
7922752	ENST00000362967		0.0089587
8074716	NR_003267	GGT3P	0.0089618
8102751	NM_144643	SCLT1	0.0089673
8066258	NR_002911	SNORA71A	0.0089856
7951796	NM_001077639	FAM55D	0.0090103
8044236	NM_181453	GCC2	0.0090273
8043040	NR_003663	FUNDC2P2	0.0091445
7966202	NM_031954	KCTD10	0.0091598
7909898	NM_198551	MIA3	0.0091676
7959148	NM_012240	SIRT4	0.0092146
7995096	NM_001145808	ITGAM	0.0092479
7906223	NM_015997	RRNAD1	0.0092767
8095697	NM_001511	CXCL1	0.0093376
7982854	NM_019074	DLL4	0.0095102
8139656	NM_001001555	GRB10	0.0095124
7961182	NM_002260	KLRC2	0.0095743
7906163	NM_020407	RHBG	0.0096305
8043639	NM_001077400		0.0096335
7894568			0.0096376
8144880	NM_022071	SH2D4A	0.0097191
8144874	AY176665	NSAP11	0.0097193
8114185	NM_080656	CDKN2AIPNL	0.0097385
8025129	NM_144614	MBD3L2	0.0097491
8134122	NM_005751	AKAP9	0.0097558
8028254	NM_001013659	ZNF793	0.0097997
7950933	NM_016931	NOX4	0.0098796
8139796	AK128010	LOC441233	0.0099051
7993110	AF204269		0.0099164
8156577	ENST00000423437		0.0099174
8010614	NM_012418	FSCN2	0.0099219
8068460	NM_015358	MORC3	0.0099634

Down-regulated gene features following siRNA-mediated *LAPTM4B* knockdown

Probeset	Accession	Symbol	p-value
8147503	NM_018407	LAPTM4B	0
8104930	NM_004172	SLC1A3	0.0000003
8096301	NM_001040058	SPP1	0.0000003
7902452	NM_174858	AK5	0.0000004
8104901	NM_002185	IL7R	0.0000006
7904843	NM_002614	PDZK1	0.0000009

7962559	NM_018018	SLC38A4	0.000001
8090433	NM_007283	MGLL	0.0000017
8099721	NM_015187	SEL1L3	0.0000034
8140534	NM_006379	SEMA3C	0.000004
7922976	NM_000963	PTGS2	0.0000044
8102800	NM_014331	SLC7A11	0.0000052
8126658	NM_178148	SLC35B2	0.0000055
8155930	NM_001490	GCNT1	0.0000066
7977933	NM_012244	SLC7A8	0.0000101
7918487	NM_024901	DENND2D	0.0000103
8042788	NM_001615	ACTG2	0.000011
7973433	NM_182908	DHRS2	0.0000115
8156199	NM_004938	DAPK1	0.0000133
8072626	NM_000362	TIMP3	0.0000156
7933772	NM_020987	ANK3	0.0000167
8136557	NM_001130966	TBXAS1	0.0000176
8119088	NM_078467	CDKN1A	0.0000178
8008598	NM_001102402	PCTP	0.0000193
8051583	NM_000104	CYP1B1	0.0000202
8168749	NM_014467	SRPX2	0.0000204
8090070	NM_183357	ADCY5	0.0000209
7953021	NM_024551	ADIPOR2	0.0000211
8046895	NM_177454	FAM171B	0.0000219
8146921	NM_172037	RDH10	0.0000285
7983630	NM_002009	FGF7	0.0000293
8180376	NM_001353	AKR1C1	0.0000295
8108370	NM_001964	EGR1	0.0000298
7906919	NM_001102445	RGS4	0.0000299
8180411	NM_130442	ELMO1	0.000031
7896688			0.000031
7938702	NR_026750		0.0000319
8112731	NM_004101	F2RL2	0.0000321
8045664	NM_177964	LYPD6B	0.0000364
8138258	NM_001135924	VWDE	0.0000397
8064100	NM_024299	PPDPF	0.0000398
7908459	NM_000186	CFH	0.00004
8133818	NM_001127358	PHTF2	0.000041
7923608	NM_000537	REN	0.000043
8151423	NM_020647	JPH1	0.0000448
7939546	NM_002231	CD82	0.0000479
8126784	NM_001168357	PLA2G7	0.0000479
7917530	AL832451		0.0000489
8114249	NM_004887	CXCL14	0.0000524
8072678	NM_002133	HMOX1	0.0000524
8155673	NM_003558	PIP5K1B	0.0000529
8112107	NM_003711	PPAP2A	0.0000531
7963187	NM_001113546	LIMA1	0.000056
8127854	NM_002395	ME1	0.0000576
8094911	NM_020453	ATP10D	0.0000585
8162283	NM_004560	ROR2	0.0000585
8011193	NR_028504	MIR22HG	0.0000586
8023727	NM_032160	DSEL	0.0000603
7931353	NM_006504	PTPRE	0.0000614
7955613	NM_005556	KRT7	0.0000646
8151816	NM_005261	GEM	0.0000651

7969665	NM_153456	HS6ST3	0.000066
7924388	NM_006085	BPNT1	0.0000678
8099760	NM_015230	ARAP2	0.0000724
7972239	NM_032229	SLITRK6	0.000075
8027862	NM_005306	FFAR2	0.0000766
8056877	NM_001039523	CHRNA1	0.0000777
7896665			0.0000843
7914342	NM_004102	FABP3	0.0000851
8021081	NM_001128588	SLC14A1	0.0000864
8166184	NM_007220	CA5B	0.0000866
8157487	NM_002581	PAPPA	0.0000866
8128087	NM_002042	GABRR1	0.000089
7908351	NM_024420	PLA2G4A	0.0000897
8095585	NM_001098484	SLC4A4	0.0000907
7896265			0.0000913
7948995	ENST00000398868		0.0000952
8080964	NM_001080393	GXYLT2	0.0000989
8098204	NM_001873	CPE	0.0001011
8176133	NM_000402	G6PD	0.0001029
8150698	NM_003068	SNAI2	0.0001061
8045336	NM_001508	GPR39	0.0001175
7921806	NM_003779	B4GALT3	0.0001229
7896340			0.0001256
8143188	NM_194071	CREB3L2	0.0001308
8114787	NM_005471	GNPDA1	0.0001331
7986293	NM_018349	MCTP2	0.0001374
8120043	NM_001024630	RUNX2	0.0001467
8005134	NM_000676	ADORA2B	0.0001469
7895213			0.0001479
8091780	NM_001038628	B3GALNT1	0.0001523
8116610	NM_000904	NQO2	0.0001549
8122396	NM_016108	AIG1	0.0001569
8041048	NM_005253	FOSL2	0.0001677
8113709	NM_002317	LOX	0.0001683
8047487	NM_003507	FZD7	0.0001761
8099850	NM_024943	TMEM156	0.0001771
8082869	NM_002718	PPP2R3A	0.00018
8148040	NM_052886	MAL2	0.0001826
7894754			0.0001878
7948332	NM_004811	LPXN	0.0001888
8084794	NM_002182	IL1RAP	0.0001991
8097038	NM_004784	NDST3	0.0002046
8016832	NM_012329	MMD	0.0002164
7893378			0.0002217
8101762	NM_000345	SNCA	0.0002275
8081564	NM_198196	CD96	0.0002324
8070579	NM_003225	TFF1	0.0002341
8136388	NM_018295	TMEM140	0.0002341
7950731	NM_199418	PRCP	0.0002342
7984932	NM_138967	SCAMP5	0.0002366
8129677	NM_001143676	SGK1	0.000238
7938880	NM_001098520	HTATIP2	0.0002443
7996761	NM_012320	PLA2G15	0.0002479
8098441	NM_001080477	ODZ3	0.0002487
8013341	NM_002404	MFAP4	0.0002535

7966035	NM_006825	CKAP4	0.0002549
8138504	NM_012294	RAPGEF5	0.0002562
8126820	NM_153840	GPR110	0.0002566
7973377	NM_004050	BCL2L2	0.0002604
8094789	NM_014988	LIMCH1	0.0002604
8103769	NM_000860	HPGD	0.0002629
8056303	NM_033272	KCNH7	0.0002632
8083839	NM_014373	GPR160	0.0002647
7917516	NM_002053	GBP1	0.0002703
8083749	NM_139245	PPM1L	0.0002736
8173503	NM_001024455	RGAG4	0.0002837
7906764	NM_002155	HSPA6	0.0002844
8032829	NM_001080400	PLIN4	0.0002909
7910022	NM_152495	CNIH3	0.0002994
8175393	NM_004840	ARHGEF6	0.0003004
7982597	NM_003246	THBS1	0.0003012
7975779	NM_005252	FOS	0.0003025
8012953	NM_006470	TRIM16	0.000305
8046333	NM_024843	CYBRD1	0.0003066
7917532	NM_004120	GBP2	0.000308
7936734	NM_000141	FGFR2	0.0003109
8004867	NM_001025579	NDEL1	0.000312
7965964	NM_032148	SLC41A2	0.0003148
8044212	NM_001056	SULT1C2	0.000316
8145669	NM_001008711	RBPMS	0.00032
8085360	NM_003256	TIMP4	0.0003245
7924977	NM_024554	PGBD5	0.0003351
8115099	NM_002609	PDGFRB	0.0003365
7947512	NM_015430	PAMR1	0.0003423
8004266	NM_201566	SLC16A13	0.000344
8112139	NM_002184	IL6ST	0.0003466
8108697	NM_015669	PCDHB5	0.0003578
8006531	NM_144975	SLFN5	0.0003623
8027002	NM_004864	GDF15	0.0003638
7919055	NM_005518	HMGCS2	0.0003642
7988467	NM_000138	FBN1	0.0003685
8056005	NM_001105	ACVR1	0.0003789
7999909	NM_016235	GPRC5B	0.0003809
8138489	NM_018719	CDCA7L	0.0003878
8149500	NM_001001924	MTUS1	0.0003999
7929779	NM_000392	ABCC2	0.0004129
7961371	NM_030640	DUSP16	0.0004134
8171435	NM_003662	PIR	0.0004139
8128565	NM_022361	POPDC3	0.0004184
8068022	NR_030784	MIR155	0.0004218
8121601	NM_153711	FAM26E	0.0004506
8081375	NM_145037	FAM55C	0.000457
8046824	AK092099	FSIP2	0.0004611
8005475	NM_001037330	TRIM16L	0.0004617
8086419	NR_027753	HHATL	0.0004623
8013833	NM_178860	SEZ6	0.0004834
7986394	NM_144598	LRRC28	0.0005021
7946292	NM_016229	CYB5R2	0.0005041
8156848	NM_006981	NR4A3	0.000509
8157038	NM_080546	SLC44A1	0.0005097

7964484	NM_001478	B4GALNT1	0.0005128
8114050	NM_015146	8-Sep	0.0005202
7953569	NM_080549	PTPN6	0.0005254
8140478	NM_017439	PION	0.0005365
8170390	NM_000202	IDS	0.0005406
7948997	NM_015459	ATL3	0.0005442
8019964	NM_001010000	ARHGAP28	0.0005475
7974341	NM_053064	GNG2	0.0005481
7950042	NM_012309	SHANK2	0.0005526
8142143	NM_006348	COG5	0.0005645
8008454	NM_003786	ABCC3	0.0005684
8122689	AY358952	C6orf72	0.0005686
7955943	NM_000924	PDE1B	0.0005775
8012304	NM_021210	TRAPPC1	0.0005848
8162940	NM_005502	ABCA1	0.0005885
8097586	NM_207123	GAB1	0.0005961
8081386	NM_031419	NFKBIZ	0.0005969
8090715	NM_032169	ACAD11	0.0006074
7893163			0.0006108
8175666	NM_004961	GABRE	0.0006149
7950641	NM_023930	KCTD14	0.0006213
7935521	NM_021732	AVPI1	0.0006246
8027402	NM_001238	CCNE1	0.0006353
7980080	NM_001249	ENTPD5	0.0006402
7957417	NM_152588	TMTC2	0.0006444
7895281			0.0006625
8101701	NM_152542	PPM1K	0.0006847
8127534	NM_138441	MB21D1	0.000685
7917779	NM_002061	GCLM	0.0006894
7994609	NM_014298	QPRT	0.0006971
8070574	NM_005423	TFF2	0.0007027
8139057	NM_014800	ELMO1	0.0007112
7943387	BC006128	C11orf70	0.0007417
7913824	NM_020317	C1orf63	0.0007644
7966046	NM_001033050	MTERFD3	0.000769
7981020	NM_016150	ASB2	0.0007755
8180310	NM_005494	DNAJB6	0.000784
7893134			0.0007866
8068810	NM_018964	SLC37A1	0.0007883
7965403	NM_002345	LUM	0.0007898
8119974	NM_001078175	SLC29A1	0.0008021
8169811	NM_000276	OCRL	0.0008034
7909332	NM_001114752	CD55	0.0008034
8175593	NM_000202	IDS	0.0008116
7896730			0.0008137
8133876	NM_001001548	CD36	0.0008148
7945663	NM_001170820	IFITM10	0.0008151
7930413	NM_004419	DUSP5	0.000817
8059186	NM_002846	PTPRN	0.0008228
8112967	NM_174909	TMEM167A	0.000834
7922717	NM_002928	RGS16	0.0008376
8041820	NM_014011	SOCS5	0.0008411
8158684	ENST00000458976		0.0008454
7920552	NM_002249	KCNN3	0.0008467
7968417	NM_023037	FRY	0.0008775

8051187	NM_022823	FNDC4	0.0008822
8107632	NM_014035	SNX24	0.0008947
7975066	NM_004857	AKAP5	0.0008955
7970716	NM_153371	LNX2	0.000904
8131881	NM_199136	C7orf46	0.0009054
8094778	NM_004181	UCHL1	0.0009173
8042503	NM_002357	MXD1	0.0009176
8075310	NM_002309	LIF	0.0009235
7959500	NM_003959	HIP1R	0.0009359
8090690	NM_130808	CPNE4	0.0009713
7974366	NM_000956	PTGER2	0.0009742
8175871	NM_000425	L1CAM	0.0009757
8072710	NM_030641	APOL6	0.0009946
8142585	NM_017954	CADPS2	0.0009985
8103853	NR_027107	MGC45800	0.0010037
8138277	NM_001135924	VWDE	0.0010188
8009432	NM_016627	AMZ2	0.0010226
8077441	NM_003670	BHLHE40	0.0010388
7929388	NM_016341	PLCE1	0.0010452
7904812	NM_006099	PIAS3	0.0010453
8122660	NM_005715	UST	0.0010472
8135341	NM_152750	CDHR3	0.0010665
7920278	NM_002960	S100A3	0.0010704
7922598	NM_004673	ANGPTL1	0.001073
8008885	NR_029493	MIR21	0.0010747
8142096	NM_020725	ATXN7L1	0.0010825
7958950	BC022092	C12orf52	0.0010939
8096176	NM_080683	PTPN13	0.0011
8046373	NM_178120	DLX1	0.0011012
7973306	NM_022060	ABHD4	0.0011124
8166230	NM_018360	TXLNG	0.0011141
8166408	NM_000444	PHEX	0.0011263
8102468	NM_003619	PRSS12	0.0011768
8149399	NM_152271	LONRF1	0.0011789
8046086	NM_203463	CERS6	0.0012044
8172268	NR_029636	MIR222	0.0012098
8145977	NM_021623	PLEKHA2	0.0012307
7950743	NM_014488	RAB30	0.0012326
7927964	NM_002727	SRGN	0.0012541
7912374	NM_003132	SRM	0.001255
8060850	NM_001200	BMP2	0.0012628
7926545	NM_032812	PLXDC2	0.0012641
8123744	NM_000129	F13A1	0.0012658
8113666	NM_020796	SEMA6A	0.0012922
8137526	NM_005542	INSIG1	0.0012943
7926105	NM_001002295	GATA3	0.0012975
7973900	NR_002937	C14orf19	0.0013009
8119161	NM_002648	PIM1	0.0013042
7915659	NM_024602	HECTD3	0.001309
8037205	NM_001712	CEACAM1	0.0013112
7977736	NM_032846	RAB2B	0.0013338
8054364	NM_004257	TGFBRAP1	0.0013354
8095751	NM_015393	PARM1	0.0013648
8166127	NM_002063	GLRA2	0.0013674
8050190	NM_003183	ADAM17	0.0013727

8085556	NM_004844	SH3BP5	0.0013764
7955063	NM_001143842	TMEM106C	0.0013885
7922095	NR_026550	BRP44	0.0013906
8075637	NM_004737	LARGE	0.0014102
7909954	NM_032890	DISP1	0.0014279
8176219	NM_001018025	MTCP1	0.0014324
7927606	NM_001098512	PRKG1	0.0014447
8136095	NM_015328	AHCYL2	0.0014717
7971461	NM_002298	LCP1	0.001475
7975076	NM_021979	HSPA2	0.0014836
8123463	NM_018288	PHF10	0.0014842
7927936	NM_004728	DDX21	0.0014876
8058182	NM_173822	FAM126B	0.0014907
7984112	NM_016530	RAB8B	0.0014911
8070567	NM_003226	TFF3	0.0014919
7970831	NM_007106	UBL3	0.0014922
7980381	NM_213601	TMED8	0.0014953
7976322	NM_001275	CHGA	0.0015021
8056784	NM_004405	DLX2	0.0015181
8022803	NM_022751	FAM59A	0.0015307
7896131			0.0015425
8151512	NM_018440	PAG1	0.0015463
7956658	NM_004731	SLC16A7	0.0015653
8040742	NM_012326	MAPRE3	0.0015686
7953765	NM_020734	RIMKLB	0.001586
7985147	NM_018602	DNAJA4	0.0015931
8152333			0.0016007
7893877			0.0016251
8047078	NM_017694	MFSD6	0.0016459
7990165	NM_020214	PARP6	0.0016746
8043413	NM_144563	RPIA	0.0016855
7969204	NM_052950	WDFY2	0.0016943
8161192	NM_194328	RNF38	0.0017
7894136			0.0017203
7951686	NM_001562	IL18	0.001731
7938390	NM_001124	ADM	0.0017364
7896317			0.0017375
7942135	NM_018043	ANO1	0.0017435
8058498	NM_003468	FZD5	0.0017481
8163149	NM_002829	PTPN3	0.0017528
8122986	NM_016224	SNX9	0.0017669
7910915	NM_000740	CHRM3	0.0017703
7973084	NM_001145	ANG	0.0017751
8002303	NM_000903	NQO1	0.0017775
8016018	NM_001143780	SLC25A39	0.0017799
7903586	NM_020141	TMEM167B	0.0018041
8039905	NM_020141	TMEM167B	0.0018041
8085797	NM_001128176	THRB	0.0018068
7992905	NM_001083601	NAA60	0.0018113
7893525			0.0018139
8126153	NM_003740	KCNK5	0.0018588
8113433	NM_001962	EFNA5	0.0018589
8115875	NM_000794	DRD1	0.0018599
8083166	NM_003304	TRPC1	0.0018998
8115327	NM_003118	SPARC	0.0019301

7960283	NM_172364	CACNA2D4	0.0019385
8011884	NM_033004	NLRP1	0.0019551
8085914	NM_003615	SLC4A7	0.0019689
7908125	NM_015149	RGL1	0.0019852
8129985	NM_006718	PLAGL1	0.0019926
7995681	NM_004530	MMP2	0.0019958
8072015	NM_005160	ADRBK2	0.0020002
8081055	NM_014043	CHMP2B	0.0020281
7894383			0.0020406
7903162	NM_152487	TMEM56	0.0020443
7917304	NM_018298	MCOLN3	0.0020485
7895678			0.0020784
7962058	NM_175861	TMTC1	0.002092
8038785	NM_001163922	VSIG10L	0.0021068
7962579	NM_001143668	AMIGO2	0.0021138
7910001	NM_003676	DEGS1	0.0021174
8112841	NM_004272	HOMER1	0.0021263
7892537			0.0021316
7894507			0.0021461
7899173	NM_024887	DHDDS	0.0021732
8067839	NR_003674	KGFLP1	0.002186
8121838	NM_001003395	TPD52L1	0.0021932
8101304	NM_152545	RASGEF1B	0.0022049
8136849	NM_001143679	GSTK1	0.0022077
8099897	NM_003359	UGDH	0.00221
7917792			0.0022109
8001932	NM_033309	B3GNT9	0.0022263
7949067	NM_004322	BAD	0.0022289
7942674	NM_015516	TSKU	0.0022309
8140468	NM_017439	PION	0.0022505
7989037	NM_004748	CCPG1	0.0022547
7925929	NM_003739	AKR1C3	0.0022618
7975506	NM_015604	DCAF4	0.0022645
7914021	NM_003047	SLC9A1	0.0022823
8132055	NM_152793	C7orf41	0.0022852
7902874	NM_032270	LRRC8C	0.0022921
7892548			0.0023137
8114814	NM_000176	NR3C1	0.0023148
8145293	NM_014265	ADAM28	0.0023274
8101587	NM_138982	MAPK10	0.0023432
8160431	NR_027054	MIR31HG	0.0023618
7893823			0.0023742
8067185	NM_001719	BMP7	0.0023861
7895254			0.0023933
8175269	NM_001166599	FAM122B	0.0024271
7983239	NM_001015001	CKMT1A	0.0024381
7983256	NM_001015001	CKMT1A	0.0024381
8013157	NM_001033551	TOM1L2	0.002446
7950683	NM_024678	NARS2	0.0024513
8081959	NM_014980	STXBP5L	0.0024655
8177851	NM_001161376	C6orf136	0.0024861
8179167	NM_001161376	C6orf136	0.0024861
8111234	NM_004061	CDH12	0.0024983
8017850	NM_017983	WIPI1	0.0025318
7917199	NM_024686	TTLL7	0.0025523

8061564	NM_181353	ID1	0.0025596
8110022	NM_003945	ATP6V0E1	0.0025619
8122045	NM_016377	AKAP7	0.0025627
8001800	NM_001797	CDH11	0.0025763
8158406	NM_018201	TBC1D13	0.0025834
8166925	NM_000240	MAOA	0.0026344
8105153	NM_012343	NNT	0.0026347
7917912	NM_000110	DPYD	0.0026442
8020825	ENST00000459168		0.0026632
7893183			0.0026892
8076384	AK096917		0.0027006
8049187	NM_025202	EFHD1	0.0027009
7967463	NM_178314	RILPL1	0.0027025
7928119	NM_004096	EIF4EBP2	0.0027319
7964733	NR_026825	RPSAP52	0.0027418
7971150	NM_005780	LHFP	0.0027517
8142524	NM_012338	TSPAN12	0.0027574
7938100	NM_000543	SMPD1	0.0027581
7955469	NM_001039960	SLC4A8	0.0027584
8103466	NM_020116	FSTL5	0.0027735
7942596	NM_001235	SERPINH1	0.0027812
7966052	NM_004075	CRY1	0.002799
7927120	NM_020975	RET	0.0028164
8046833	AK126104	FSIP2	0.002831
8093906	NM_006005	WFS1	0.0028467
8133670	NM_000941	POR	0.0028595
7895371			0.0028843
8056343	NM_014900	COBLL1	0.0029305
8025402	NM_139314	ANGPTL4	0.0029479
7975932	NM_020431	TMEM63C	0.0029891
7900365	NM_001136493	MFSD2A	0.0030173
8180359	NM_001002811	PDE4DIP	0.0030246
7951372	NM_033306	CASP4	0.0030264
8045289	NM_012249	RHOQ	0.0030271
7943892	NM_181351	NCAM1	0.003029
7957140	NM_003667	LGR5	0.0030467
7918504	NM_002557	OVGP1	0.0030481
7896575			0.0030482
7930148	NM_178858	SFXN2	0.0030599
8144121	NM_002847	PTPRN2	0.0030636
8062427	NM_080607	VSTM2L	0.0030864
7897044	NM_002744	PRKCZ	0.0031055
8134318	NM_022900	CASD1	0.0031076
8047467	NM_139158	CDK15	0.0031131
8054064	NM_025190	ANKRD36B	0.0031329
7948910	NR_002565	SNORD25	0.003147
8111136	NM_001034850	FAM134B	0.0031636
8093950	NM_005980	S100P	0.0031972
7970509	ENST00000419579		0.003269
7894069			0.0032875
8022356	NM_001128626	SPIRE1	0.0033046
8066953	NM_006038	SPATA2	0.0033408
8070297	NM_001136154	ERG	0.0033486
8083594	NM_002852	PTX3	0.0033499
7916229	NM_018281	ECHDC2	0.0033768

8140730	NM_024315	C7orf23	0.0033857
8102006	NM_005908	MANBA	0.0033898
8018922	NM_004762	CYTH1	0.0034095
7955589	NM_002135	NR4A1	0.0034322
8013384	NM_000691	ALDH3A1	0.0034407
8088820	NM_012234	RYBP	0.0034782
8100541	NM_001553	IGFBP7	0.0035115
7894410			0.003529
7925457	NM_002924	RGS7	0.003533
8014454	NM_001163735	MYO19	0.0035386
8033097	NM_000635	RFX2	0.0035431
8024299	NM_001018	RPS15	0.0035515
7902382	NM_004582	RABGGTB	0.0035517
8105487	NM_138453	RAB3C	0.003553
7896182			0.0035647
7979085	NM_002863	PYGL	0.0035715
8058221	NM_015049	TRAK2	0.0035739
7972297	NM_005845	ABCC4	0.0035761
8144758	NM_016353	ZDHHC2	0.003577
7925589	NM_001167740	SMYD3	0.0035851
8035304	NM_004335	BST2	0.0036206
8102792	NM_019035	PCDH18	0.0036265
7942889	NM_032273	TMEM126A	0.0036403
7933665	NM_000242	MBL2	0.0036479
7997289	NM_033401	CNTNAP4	0.0036684
8148448	NM_006558	KHDRBS3	0.0036807
7892737			0.0036931
8151401	NM_001001481	UBE2W	0.0036932
8120833	NM_031469	SH3BGRL2	0.0037049
7902565	NM_012302	LPHN2	0.0037224
8175647	NM_031462	CD99L2	0.0037253
8110932	NM_003966	SEMA5A	0.0037277
7952339	NR_001453	SNORD14C	0.0037321
8089011	NM_000313	PROS1	0.0037662
7948741	NM_012200	B3GAT3	0.0037729
7979204	NM_006832	FERMT2	0.0037805
8086330	NM_033027	CSRNP1	0.0038065
7958174	NM_003330	TXNRD1	0.0038746
7925939	NM_001818	AKR1C4	0.0038795
7926189	NM_018144	SEC61A2	0.0038833
8100085	NM_138335	GNPDA2	0.0038845
8149071	NM_001147	ANGPT2	0.0038869
8114593	NM_133174	APBB3	0.0038877
8103025	NM_178835	ZNF827	0.0038987
7977868	NM_021944	C14orf93	0.0039032
7933872	NM_000399	EGR2	0.0039186
7989193			0.0039272
8155883	NM_012383	OSTF1	0.0039352
8162039	NM_032307	C9orf64	0.0039452
7893459			0.0039469
8084217			0.0039542
7908488	NM_002113	CFHR1	0.0039712
7993680	BC050464	C16orf62	0.0039897
8028652	NM_003407	ZFP36	0.0040084
7932285	NM_024948	FAM188A	0.0040507

8107920	NM_003060	SLC22A5	0.0040636
7896365			0.0040652
8105862	NM_001799	CDK7	0.0040881
8177462	NM_001799	CDK7	0.0040881
7999889	NM_016641	GDE1	0.0041034
8088642	NM_015541	LRIG1	0.004118
8036207	NM_139239	NFKBID	0.0041386
7997491	NM_002153	HSD17B2	0.0041902
7988208	NR_002318	CATSPER2P1	0.0042266
7906900	AY423733	DDR2	0.0042277
8013965	NM_033389	SSH2	0.0042318
8075635	NM_000362	TIMP3	0.0042405
8155169	NM_021111	RECK	0.0042521
8120585	NM_001044305	SMAP1	0.0042572
8070239	NM_000411	HLCS	0.0042586
8050427	NM_030797	FAM49A	0.0042788
8171537	ENST00000447790		0.0042857
7950307	NM_003355	UCP2	0.0043322
8021496	NM_020854	KIAA1468	0.0043348
7893527			0.0043781
8017582	NM_018469	TEX2	0.0043923
7918275	NM_001142550	WDR47	0.0044375
7892561			0.0044525
8005661	NM_001033553	SPECC1	0.004508
8152606	NM_021021	SNTB1	0.0045271
8124134	NM_000367	TPMT	0.0045532
7895534			0.0045583
8147132	NM_000067	CA2	0.0045691
7949503	NM_016938	EFEMP2	0.0045753
7999387	NM_001424	EMP2	0.0045823
8081686	NM_033254	BOC	0.0045825
8022488	NM_138340	ABHD3	0.0045863
7896565			0.0046018
8175457	NM_001171876	MCF2	0.0046167
7895114			0.0046255
8032608	NM_174983	C19orf28	0.0046556
8008646	NM_021626	SCPEP1	0.004666
8053315	NM_024993	LRRTM4	0.0046962
7967117	NM_003733	OASL	0.0046996
7985268	NM_000137	FAH	0.0047424
7929373	NM_005097	LGI1	0.0047451
8036291	NM_001042474	ZNF565	0.0047687
8030171	NM_000146	FTL	0.0047845
8074856	NM_206953	PRAME	0.0047904
8050537	NM_002381	MATN3	0.0048066
7907370	NM_015569	DNM3	0.0048207
8144267	NM_018941	CLN8	0.0048246
8095744	NM_001657	AREG	0.0048527
8064375	NM_080725	SRXN1	0.0048567
8115831	NM_004417	DUSP1	0.0048787
8090507	GENSCAN00000057606		0.0048884
8127346	NM_016277	RAB23	0.0048911
7936777	NM_017615	NSMCE4A	0.0048995
7948088	NM_032315	SLC25A33	0.0049006
8101429	NM_016619	PLAC8	0.00491

7974461	NR_003225	LGALS3	0.004921
8091954	NM_014498	GOLIM4	0.0049252
8044499	NM_005415	SLC20A1	0.0049506
8152703	NM_058229	FBXO32	0.0049605
8128698	NM_014454	SESN1	0.0049698
8144279	NM_018941	CLN8	0.0049999
8059580	NM_139072	DNER	0.0050047
7926715	NM_020752	GPR158	0.0050075
7909866	NM_017898	MOSC2	0.0050296
8014144	NM_183377	ACCN1	0.0050332
8096808	NM_017918	CCDC109B	0.0050452
7894020			0.0050587
8130939	NM_005618	DLL1	0.0050707
7893635			0.0050758
8017711	NM_006572	GNA13	0.00509
7895389			0.0051242
8154846			0.0051265
8173869	NM_024921	POF1B	0.0051875
7985317	NM_018689	KIAA1199	0.0051968
8132290	ENST00000457187		0.0052182
7906128	NM_014655	SLC25A44	0.0052184
7895993			0.0052265
7951703	NM_000795	DRD2	0.0052746
8129273	NM_152730	C6orf170	0.0052786
8056763	ENST00000433675		0.0052926
8002778	NM_152649	MLKL	0.0053163
8099541	NM_000320	QDPR	0.0053183
7917649	NM_003243	TGFBR3	0.0053244
8155487	NR_003674	KGFLP1	0.0054357
8161423	NR_003674	KGFLP1	0.0054357
8161455	NR_003674	KGFLP1	0.0054357
8006123	NM_001304	CPD	0.0054359
8102482	NM_014822	SEC24D	0.0055526
8068238	NM_207585	IFNAR2	0.0055822
7895255			0.0056353
7909789	NM_001135599	TGFB2	0.0056407
7997680	NM_014615	KIAA0182	0.0056555
8128939	NR_028338	TRAF3IP2	0.0056833
8117987	NM_001161376	C6orf136	0.0056869
8127193	NM_021073	BMP5	0.0057066
7893851			0.005744
8112865	NM_178276	SERINC5	0.0057452
8140463	NM_006682	FGL2	0.0058145
8044793	NM_182915	STEAP3	0.005822
7942232	NM_018161	NADSYN1	0.0058252
7985809	NM_007011	ABHD2	0.0058486
8048195	NM_000998	RPL37A	0.0058702
7892702			0.0058746
8007154	NM_021939	FKBP10	0.0059097
8138718	NM_006735	HOXA2	0.0059124
8040458	NM_002252	KCNS3	0.0059327
8132031	NM_175887	PRR15	0.0059358
7896067			0.0059582
7982938	NM_006293	TYRO3	0.0059759
8137448	NM_022087	GALNT11	0.0059805

8145603	NM_006228	PNOC	0.0060299
8157761	NM_001145001	NEK6	0.0060375
7914202	AY277594	SNHG12	0.0060375
7947110	NM_024680	E2F8	0.0060436
7939751	NM_005693	NR1H3	0.0060482
8063386	NM_005194	CEBPB	0.0060951
7918379	NM_000849	GSTM3	0.0061322
7928208	NM_018344	SLC29A3	0.0061324
8146122	NM_016099	GOLGA7	0.0061408
7954527	NM_020183	ARNTL2	0.0061435
7906061	NM_152280	SYT11	0.006147
8066407	NM_001080472	FITM2	0.0061516
8078386	NM_015141	GPD1L	0.0061591
8051427	NM_005102	FEZ2	0.0062376
8110569	NM_003900	SQSTM1	0.0062593
7896437			0.0062695
8017964	NM_080284	ABCA6	0.0062716
8068168	NM_000454	SOD1	0.0062806
7895677			0.0062835
7898192	NM_015291	DNAJC16	0.0062837
8127158	NM_001498	GCLC	0.0062927
8150889	NM_138969	SDR16C5	0.006333
7921713	NM_016946	F11R	0.0063333
8163257	NM_057159	LPAR1	0.0063559
7893898			0.0063898
8107814	NM_016048	ISOC1	0.0064344
7896177			0.0064373
8101971	NM_000944	PPP3CA	0.0064571
8096116	NM_032717	AGPAT9	0.006468
7979455	NM_021136	RTN1	0.0064733
8084630	BX640843		0.0064758
8110114	NM_006650	CPLX2	0.0064876
7969171	NR_002605	DLEU1	0.0065078
7893618			0.0065133
8121613	NM_016104	RWDD1	0.00653
8162610	NM_033331	CDC14B	0.0065617
8006229	NM_032322	RNF135	0.0065982
8133459	NM_003388	CLIP2	0.00662
7924582	NM_025160	WDR26	0.0066206
8057933	NM_012086	GTF3C3	0.0066708
8167103	NM_006201	CDK16	0.0066739
8136115	NM_020704	FAM40B	0.0066885
8121588	NM_013352	DSE	0.0067245
8089835	NM_007085	FSTL1	0.0067292
8053562	NM_016079	VPS24	0.0067887
8128956	NM_002037	FYN	0.0068138
7892931			0.0068611
7893705			0.0068706
8077160	NM_000487	ARSA	0.0068725
8125139	NM_000434	NEU1	0.0068737
8179851	NM_000434	NEU1	0.0068737
8122982	NM_024630	ZDHHC14	0.0068827
8068363	NR_027267	LINC00310	0.0069215
7895291			0.0069275
7978748	NM_203301	FBXO33	0.006949

8075052	NM_182492	LRP5L	0.0069527
8081880	NM_001125	ADPRH	0.0069866
8154381	NM_203403	C9orf150	0.0069889
8093258	NM_032263	IQCG	0.0070038
7957023	NM_000239	LYZ	0.0071007
8017704	NR_026903	AMZ2P1	0.0071264
8080762	NM_020676	ABHD6	0.0071409
8130422	NM_173515	CNKSR3	0.0071839
7918857	NM_005725	TSPAN2	0.0071998
7895998			0.0072006
8150103	NM_002095	GTF2E2	0.0072013
7993713	NM_153208	IQCK	0.0072042
8084016	NM_006218	PIK3CA	0.0072061
7906863	NM_003115	UAP1	0.0072099
8172158	NM_003688	CASK	0.0072375
8012464	NR_024447	LOC100128288	0.0072461
7908204	NM_031935	HMCN1	0.0072487
8101675	NM_004827	ABCG2	0.0073387
7968254	NM_152705	POLR1D	0.0073496
7895931			0.0073568
7977841	NM_001166269	HAUS4	0.0073638
8034837	NM_006145	DNAJB1	0.0073777
7895257			0.0073858
7962375	NM_153026	PRICKLE1	0.0073882
7895309			0.007403
7929711	NM_001010917	GOLGA7B	0.0074588
8061706	NM_015352	POFUT1	0.0074802
8148715	NM_003801	GPAA1	0.0075013
8150962	NM_014729	TOX	0.0075505
8056151	NM_007366	PLA2R1	0.0075541
8044094	NM_144632	TMEM182	0.0075654
8151952	NM_024759	NIPAL2	0.0076007
8092707	NM_018192	LEPREL1	0.007603
8147469	NM_016134	PGCP	0.0076054
7971692	NM_012141	INTS6	0.0076154
7990309	NM_022369	STRA6	0.0076191
8178676	NM_000434	NEU1	0.0076227
8050160	NM_138799	MBOAT2	0.0076404
8041582	NM_138370	PKDCC	0.0076583
7956242	NM_144576	COQ10A	0.0076628
8175177	NM_018388	MBNL3	0.0076999
7973067	NM_000270	PNP	0.0077019
8029969	NM_003009	SEPW1	0.0077063
7946446	NM_020645	NRIP3	0.0077092
8094625	NM_015990	KLHL5	0.0077176
7892558			0.0077249
7954985	NM_032256	TMEM117	0.0077553
7976556	NR_023938	C14orf132	0.007783
8110147	NM_198567	C5orf25	0.0078029
7948656	NM_002032	FTH1	0.0078149
8131600	NM_014399	TSPAN13	0.0078287
8063211	NM_181659	NCOA3	0.0078338
7967473	NM_001414	EIF2B1	0.0078589
7893282			0.007869
8041179	NM_024692	CLIP4	0.0078719

8020527	NM_013326	C18orf8	0.0078798
8169389	NM_002578	PAK3	0.0078962
8152355	NM_001099750	SYBU	0.0078976
7965573	NM_021229	NTN4	0.0079206
7895598			0.0079355
7940153	NM_022074	FAM111A	0.0079362
8143341	NM_030647	JHDM1D	0.0079543
7896401			0.008009
7896759	AK096570	LOC643837	0.0080108
7992996	AY358225		0.0080137
7907160	NM_001677	ATP1B1	0.0080194
8138862	AK097240	LOC401320	0.0080232
7951309	NM_002427	MMP13	0.0080362
8131496	NM_138426	GLCCI1	0.0080627
7906671	NM_001014443	USP21	0.0080947
7948906	NR_002563	SNORD27	0.0081105
7979721	NM_182526	TMEM229B	0.0081665
7902553	NM_006417	IFI44	0.0082126
7957613	NM_017599	VEZT	0.0082425
8122933	NM_012454	TIAM2	0.0082505
8163948	NM_033117	RBM18	0.0082554
8111932	NM_148672	CCL28	0.0082705
7902127	NM_032291	SGIP1	0.0083019
8129097	NM_003309	TSPYL1	0.0083072
7941714	NM_024650	C11orf80	0.0083082
8022572	NM_080597	OSBPL1A	0.0083095
7892508			0.0083774
7906602	NM_020335	VANGL2	0.0084008
8130032	NM_032145	FBXO30	0.0084103
8112409	NM_019072	SGTB	0.0084373
7895741			0.0084531
7930181	NM_020682	AS3MT	0.0085016
8148265	NM_007218	RNF139	0.0085348
7933237	NM_174890	ANUBL1	0.0085857
8075820	NM_006078	CACNG2	0.0086055
8028744	NM_207646		0.0086696
7916808	NM_015139	SLC35D1	0.0087232
7959131	NM_207311	CCDC64	0.0087268
7895032			0.0087443
7959574	NM_020382	SETD8	0.0087628
8112857	NM_001010891	MTX3	0.0087681
7914178	ENST00000410400		0.0087818
8020806	NM_017831	RNF125	0.0088188
7961546	NM_004447	EPS8	0.0088452
8102065	NM_020139	BDH2	0.00885
8113641	NM_001801	CDO1	0.0088636
8150928	NM_003580	NSMAF	0.0088669
7938816	NM_019028	ZDHHC13	0.0088899
7961365	NM_018050	MANSC1	0.008894
7915504	NM_022821	ELOVL1	0.0089076
8089743	NM_212543	B4GALT4	0.0089103
7947815	NM_001610	ACP2	0.0089399
8105663	NM_020726	NLN	0.0089436
7917503	NM_018284	GBP3	0.0089448
7985285	NM_014862	ARNT2	0.0089964

7915363	NM_001031694	SCMH1	0.0090023
7975136	NM_178155	FUT8	0.0090078
8122365	NM_020455	GPR126	0.009021
7894753			0.0090217
7972428	NM_080818	OXGR1	0.0090429
8174361	NM_198057	TSC22D3	0.0090443
8091537	NM_178822	IGSF10	0.0090557
8148059	NM_022783	DEPTOR	0.0090664
8007828	NM_016835	MAPT	0.0090667
7892657			0.0090932
8065817	NM_000178	GSS	0.0091128
7895040			0.009121
7939839	NM_002843	PTPRJ	0.0091368
7894664			0.0091586
7979658	NM_002083	GPX2	0.0092118
7946061	NM_005330	HBE1	0.0092978
8080781	NM_017771	PXK	0.0093003
7981460	NM_015316	PPP1R13B	0.0093114
7894993			0.0093187
8094476	NM_018317	TBC1D19	0.0093342
7938687	NM_005013	NUCB2	0.0093634
7950671	NM_080491	GAB2	0.0094303
8047161	NM_001031716	OBFC2A	0.0094384
8052598	NM_015910	WDPCP	0.0094582
8110055	NM_030627	CPEB4	0.0094805
8130013	NM_005670	EPM2A	0.0094841
7892656			0.0095156
7894577			0.009538
8057677	NM_014585	SLC40A1	0.0095664
7918223	NM_144584	HENMT1	0.0095723
8123006	NM_003898	SYNJ2	0.0095757
7906852	NM_175866	UHMK1	0.009586
8078898	NM_017875	SLC25A38	0.0095863
7938750	NR_026541	LOC494141	0.0096163
7894409			0.0096379
8174005	NM_024917	TRMT2B	0.00964
7958884	NM_016816	OAS1	0.0096653
8167601	NM_001145073	USP27X	0.0097271
7894983			0.0097277
8154563	NM_001010887	ACER2	0.009732
7896286			0.0097719
7956613	NM_005981	TSPAN31	0.0097722
8096425	NM_001145065	FAM190A	0.0097812
8059111	NM_005689	ABCB6	0.0097841
8146285	NM_152419	HGSNAT	0.009792
7917954	NM_001013660	FRRS1	0.0098241
7894841			0.0098383
7896463			0.0098435
7984124	NM_031301	APH1B	0.0098527
8095728	NM_001432	EREG	0.0098709
8083223	NM_173552	C3orf58	0.0098744
8099633	NM_013261	PPARGC1A	0.0098949
8157092	NM_018112	TMEM38B	0.0099378
7938286	NM_000990	RPL27A	0.0099715
7894109			0.0099897

Supplementary Table 4. Gene features significantly differentially expressed in lung cancer cells cultured in medium containing 10% FBS and transfected with *LAPTM4B* -specific siRNA compared to cells transfected with control siRNA

Up-regulated gene features following siRNA-mediated *LAPTM4B* knockdown

Probeset	Accession	Symbol	p-value
7907222	NM_006902	PRRX1	0.0000009
8128991	NM_001105206	LAMA4	0.000002
8088264	NM_017563	IL17RD	0.0000028
8115490	NM_033274	ADAM19	0.0000037
8057990	NM_153697	ANKRD44	0.0000039
8152617	NM_005328	HAS2	0.000005
8116980	NM_001165032	RNF182	0.0000055
8153002	NM_001135242	NDRG1	0.0000058
7970954	NM_004734	DCLK1	0.0000085
8129458	NM_033515	ARHGAP18	0.0000127
8138231	NM_015204	THSD7A	0.0000134
8063942	NM_002531	NTSR1	0.0000187
8095680	NM_000584	IL8	0.000019
8059361	NM_020830	WDFY1	0.0000192
7988260	NM_032892	FRMD5	0.0000199
7922474	NM_014656	KIAA0040	0.0000239
7961166	NM_013431	KLRC4	0.0000258
8059376	NM_001136529		0.0000276
7952036	NM_198275	MPZL3	0.0000311
7938608	NM_006108	SPON1	0.0000318
8140650	NM_012431	SEMA3E	0.0000323
7919326	NM_016361	ACP6	0.0000327
7909561	NR_026761	LINC00467	0.0000352
8174322	NM_024657	MORC4	0.0000355
8091411	NM_014220	TM4SF1	0.0000368
7947147	NM_148893	SVIP	0.0000401
8067233	NM_020182	PMEPA1	0.0000404
8105302	NM_006350	FST	0.0000515
7983867	NM_032866	CGNL1	0.0000527
7903507	NM_001010883	FAM102B	0.0000557
8172043	NM_006307	SRPX	0.0000578
7961187	NM_213658	KLRC1	0.000058
7961175	NM_002261	KLRC3	0.0000642
8139207	NM_002192	INHBA	0.0000682
8082100	NM_017554	PARP14	0.0000764
7930498	NM_016234	ACSL5	0.0000819
7951271	NM_002421	MMP1	0.0000836
7895980			0.0000846
8107706	NM_005573	LMNB1	0.0000847
7988563	NM_203349	SHC4	0.0000857
7904761	NM_003637	ITGA10	0.0000878
8096160	NM_001025616	ARHGAP24	0.0000881
8152962	NM_012472	LRRC6	0.0000902
7965510	NM_020698	TMCC3	0.0000956
8119016	NM_002754	MAPK13	0.0000968
7924096	NM_002497	NEK2	0.0001029
7993588	NM_024847	TMC7	0.0001059
8174313	NM_024657	MORC4	0.0001073

7997332	NM_001105663	NUDT7	0.0001095
7991762	NM_000517	HBA2	0.000115
7991766	NM_000558	HBA1	0.000115
7908597	NM_205860	NR5A2	0.0001249
8041853	NM_002354	EPCAM	0.0001273
7951614	NM_002716	PPP2R1B	0.000128
8018761	NM_006456	ST6GALNAC2	0.0001295
8180372	NM_006456	ST6GALNAC2	0.0001295
8054479	NM_005434	MALL	0.0001314
8101260	NM_058172	ANTXR2	0.0001319
8066822	NM_018837	SULF2	0.0001335
7935337	NM_152309	PIK3AP1	0.0001376
8156116	NM_001001551	C9orf103	0.0001417
7979710	NM_016445	PLEK2	0.0001424
7914270	NM_006762	LAPTM5	0.0001445
7996081	NM_201524	GPR56	0.0001461
8007446	NM_005533	IFI35	0.000147
8026300	NM_078481	CD97	0.0001544
7973974	NM_006194	PAX9	0.0001556
8142110	NM_006754	SYPL1	0.0001573
8142763	AF194537		0.0001671
7948444	NM_001062	TCN1	0.0001707
7986385	NM_145728	SYNM	0.0001812
8082846	NM_004441	EPHB1	0.0001825
8150920	NM_000780	CYP7A1	0.0001849
7939237	NM_012194	C11orf41	0.0001967
7939932	NM_001004704	OR4C6	0.0001974
8106448	NM_003719	PDE8B	0.000203
8103601	NM_001012967	DDX60L	0.0002174
7939197	NM_005734	HIPK3	0.0002194
8154635	NM_002451	MTAP	0.0002279
8089464	ENST00000383686		0.0002368
7952914	NM_032358	CCDC77	0.0002435
7981514	NM_138420	AHNAK2	0.0002506
7893049			0.0002512
8171837	NM_030624	KLHL15	0.0002547
8122202	NM_001130173	MYB	0.0002608
8122013	NM_032438	L3MBTL3	0.0002672
8092750	NM_021032	FGF12	0.0002687
7893579			0.0002691
7893957			0.0002706
8114991	NM_024577	SH3TC2	0.0002716
7972579	NM_032813	TMTC4	0.0002817
7989243	ENST00000458913		0.0003261
7908041	NM_002293	LAMC1	0.0003301
8079237	NM_020242	KIF15	0.000332
7936463	NM_002313	ABLIM1	0.0003344
7979615	NM_001024858	SPTB	0.0003349
8046078	NM_020981	B3GALT1	0.0003368
7894891			0.0003402
7899703	NM_175852	TXLNA	0.0003414
8033813	NM_017703	FBXL12	0.000345
8026490	NR_015379	UCA1	0.0003578
7979328	NM_014924	ATG14	0.0003588
8175155	NM_194277	FRMD7	0.0003613

8089112	NM_182909	FILIP1L	0.0003637
7927799	NM_001001330	REEP3	0.0003669
8038029	NM_014959	CARD8	0.0003685
8107133	NM_000919	PAM	0.0003689
7930276	NM_014720	SLK	0.0003773
7895386			0.0003967
8088664	NM_003848	SUCLG2	0.0004213
7935403	NM_032900	ARHGAP19	0.00043
8180309	NM_152996	ST6GALNAC3	0.0004452
7961900	NM_002223	ITPR2	0.0004476
7939215	NM_012194	C11orf41	0.0004486
8077786	NM_001570	IRAK2	0.0004488
8040113	NM_003887	ASAP2	0.0004501
8089329	NM_014981	MYH15	0.0004679
7915882	NM_014774	KIAA0494	0.0004828
8046726	NM_001130445	SSFA2	0.0004911
8031744	NM_006959	ZNF17	0.0004943
8154981	NM_006377	UNC13B	0.0005059
8146000	NM_003816	ADAM9	0.0005061
8127051	NM_012288	TRAM2	0.0005123
7896379			0.0005132
8080911	NM_032505	KBTBD8	0.000528
7966878	NM_007174	CIT	0.0005313
8152053	NM_198401	ANKRD46	0.0005468
7896409			0.0005485
8049317	NM_152879	DGKD	0.000551
7896041			0.0005545
8094938	NM_207330	NIPAL1	0.0005592
8161499	BC032035	FAM27E3	0.0005619
8124492	NM_080593	HIST1H2BK	0.0005629
8134091	NM_012129	CLDN12	0.0005656
7912145	NM_001561	TNFRSF9	0.0005731
8161446	BC032035	FAM27E3	0.0005807
7943413	NM_001165	BIRC3	0.0005902
8155514	BC032035	FAM27E3	0.0006
8009380	NR_003706	SNORA38B	0.0006012
8076547	NR_027779	TTLL1	0.0006019
8073062	NM_004900	APOBEC3B	0.000608
7947189	NM_030771	CCDC34	0.0006169
7990391	NM_000499	CYP1A1	0.0006282
8142345	NM_014705	DOCK4	0.0006332
8168622	NM_019117	KLHL4	0.0006376
8012270	NM_001143990	WRAP53	0.0006436
8123407	NM_001040001		0.0006474
8057689	NM_022353	OSGEPL1	0.0006562
7947425	NM_203330	CD59	0.0006567
7972217	NM_005842	SPRY2	0.0006672
8007100	NM_001552	IGFBP4	0.0006793
8163383	NM_022486	SUSD1	0.0006942
8088491	NM_003716	CADPS	0.0007
8125919	NM_001145775	FKBP5	0.0007024
7930682	NM_020940	FAM160B1	0.0007072
7927786	NM_001001330	REEP3	0.000709
7975238	NM_020715	PLEKHH1	0.0007288
8083656	NM_022736	MFSD1	0.0007297

8032623	NM_001060	TBXA2R	0.0007301
8116504	NM_203293	TRIM7	0.0007391
7943218	NM_015368	PANX1	0.0007434
8051413	NM_015475	FAM98A	0.0007485
8155707	NM_004817	TJP2	0.0007562
7893172			0.0007574
8149555	NM_015310	PSD3	0.000764
8135568	NM_199072	MDFIC	0.000764
7934615	NM_004747	DLG5	0.0007666
7936322	NM_020918	GPAM	0.0007692
7897172	NM_182752	TPRG1L	0.0007833
8045514	NM_001001664	SPOPL	0.0007878
8138202	NM_004968	ICA1	0.0007905
7907445	NM_014458	KLHL20	0.0007949
7944869	NM_017425	SPA17	0.0008196
8169441	ENST00000403700		0.0008312
8093332	NR_027481	ZNF876P	0.0008353
8034097	NR_024333	LOC147727	0.0008366
8137670	NM_002607	PDGFA	0.0008412
8172035	NM_006520	DYNLT3	0.0008419
8044965	NM_004622	TSN	0.0008572
8010832	NM_024619	FN3KRP	0.0008726
8019857	NM_006101	NDC80	0.0008864
8115410	NM_015465	GEMIN5	0.000899
8127109	NM_016513	ICK	0.0009018
8126184	NM_145027	KIF6	0.0009061
7894356			0.0009225
7931097	NM_002775	HTRA1	0.0009235
8138977	NM_015283	DPY19L1	0.0009258
7975292	NM_002877	RAD51B	0.0009291
7903908	NM_006090	CEPT1	0.0009371
7909400	NM_002389	CD46	0.0009391
7901969	NM_005012	ROR1	0.0009405
7982663	NM_001211	BUB1B	0.0009507
7961151	NM_007360	KLRK1	0.0009527
8073088	NM_021822	APOBEC3G	0.0009549
8146685	NM_015169	RRS1	0.0009603
8064216	ENST00000362935		0.0009632
7967072	NM_032314	COQ5	0.0009829
8089867	NM_173825	RABL3	0.0009894
8121225	NM_175768	GRIK2	0.0010229
8130151	NM_139165	RAET1E	0.0010447
7905339	NM_144618	GABPB2	0.0010502
8106660	NM_006909	RASGRF2	0.0010868
8046062	NM_152381	XIRP2	0.0010895
7980233	NM_002632	PGF	0.0010945
7940191	NM_004177	STX3	0.001102
7901788	NM_001134673	NFIA	0.0011026
8149733	NM_003842	TNFRSF10B	0.0011094
8114920	NM_001387	DPYSL3	0.0011317
8001133	NM_024745	SHCBP1	0.0011735
8135211	NR_026879	FAM185A	0.001174
8026272	NM_004843	IL27RA	0.0011806
7990345	NM_003612	SEMA7A	0.0011972
8023871	NM_175907	ZADH2	0.0012143

8180375	NM_001007246	BRWD1	0.0012318
8008627	NM_005450	NOG	0.0012354
8065071	NM_198391	FLRT3	0.0012655
8129497	NM_001431	EPB41L2	0.0012733
7938348	NM_003390	WEE1	0.0012781
7942453	NM_021200	PLEKHB1	0.0012878
7943293	NM_015036	ENDOD1	0.0012921
8103834	NM_000027	AGA	0.0013195
7933821	NR_024556		0.0013253
7912706	NM_004431	EPHA2	0.001367
7943263	NM_130847	AMOTL1	0.0013679
7912292	NM_032368	LZIC	0.0013804
7934026	NM_001080449	DNA2	0.0013953
8110090	NM_022754	SFXN1	0.0013982
7923119	NM_194314	ZBTB41	0.0014322
7947199	NM_018490	LGR4	0.0014441
8014189	NM_057178		0.0014463
7939383	NM_024841	PRR5L	0.0014859
8130176	NM_024518	ULBP3	0.0014926
7900009	NM_017629	EIF2C4	0.001497
8147262	NM_016023	OTUD6B	0.001507
8042283	NM_014181	LGALSL	0.0015334
7925492	NM_014322	OPN3	0.0015356
8072454	NR_024210	RNF185	0.0015418
7916112	NM_002867	RAB3B	0.001556
8142194	NM_002291	LAMB1	0.0015719
8044346	NR_027244	LOC151009	0.0015737
7933659	NM_015235	CSTF2T	0.0015778
7911017	NM_006642	SDCCAG8	0.0015819
8149551	NM_015310	PSD3	0.0015925
8148501	NM_032611	PTP4A3	0.001605
8095187	NM_025009	CEP135	0.0016151
8060949	NM_022096	ANKRD5	0.0016352
7895036			0.0016452
7923991	NM_025179	PLXNA2	0.0016488
8111772	NM_001343	DAB2	0.0016613
8061247	NM_018993	RIN2	0.0016672
8174496	NM_015365	AMMECR1	0.0016705
7965357	NM_003774	GALNT4	0.0016713
8048995	NM_030926	ITM2C	0.0016795
7910387	NM_021205	RHOU	0.0016817
8114425	NM_001790	CDC25C	0.0016889
8113250	NM_001040458	ERAP1	0.0016911
8096556	NM_015143	METAP1	0.0016967
7957298	NM_014903	NAV3	0.0017136
7963092	NM_175736	FMNL3	0.0017218
8168794	NM_006733	CENPI	0.0017383
7967325	NM_032554	HCAR1	0.0017518
7895838			0.0017588
8063923	NM_016354	SLCO4A1	0.0017615
7906838	NM_014697	NOS1AP	0.0017665
8080645	NM_012096	APPL1	0.0017741
7959856	NM_004764	PIWIL1	0.0017772
8089082	NM_080927	DCBLD2	0.0017859
7932453	NM_006393	NEBL	0.0017868

8163839	NM_001735	C5	0.0018056
8103289	NM_002669	PLRG1	0.0018149
8095736	NM_001657	AREG	0.001826
7919747	ENST00000410192		0.0018345
8124610	NM_006510	TRIM27	0.0018425
8179575	NM_006510	TRIM27	0.0018425
7894831			0.0018491
8026163	NM_004907	IER2	0.0018645
8011817	NM_014519	ZNF232	0.0018664
7987454	NM_001003940	BMF	0.0018676
7984276	NM_004727	SLC24A1	0.0018718
8133049	NM_016220	ZNF107	0.0018736
7896659			0.0018776
8093230	NM_001145642	KIAA0226	0.0018779
7956867	NM_003483	HMGA2	0.0018862
8029340	NM_003445	ZNF155	0.0019077
8059838	NM_018410	HJURP	0.0019195
7953835	NM_005810	KLRG1	0.0019281
7957560	NM_003805	CRADD	0.0019572
7899005	NM_014313	TMEM50A	0.0019654
8109712	NM_001142556	HMMR	0.0019772
8097282	NM_005841	SPRY1	0.00201
8112388	NM_001656	TRIM23	0.0020297
8105121	NM_000163	GHR	0.0020462
7979565	NM_080666	WDR89	0.0020512
8095744	NM_001657	AREG	0.0020816
8138454	NM_001002926	TWISTNB	0.002082
8156706	NM_003275	TMOD1	0.0020909
8013567	NM_001076680	C17orf108	0.0020958
8180264	NM_001076680	C17orf108	0.0020958
8118890	NM_152753	SCUBE3	0.0020996
7897527	NM_001105562	UBE4B	0.002113
8138566	NM_006547	IGF2BP3	0.0021176
8095697	NM_001511	CXCL1	0.002128
7929065	NM_001548	IFIT1	0.0021337
8055711	NM_001164507	NEB	0.002136
8110499	NM_025158	RUFY1	0.0021441
7956930	NM_006482	DYRK2	0.0021638
8019939	NM_170695	TGIF1	0.002172
8081710	NM_017699	SIDT1	0.0021749
8157524	NR_024168	TLR4	0.0021749
7981745	ENST00000420380		0.002185
8048864	NM_004591	CCL20	0.0022005
7952408	NM_170601	SIAE	0.0022626
8092095	NM_015028	TNIK	0.0022707
8019954	NR_024101	FLJ35776	0.0022829
7917240	NM_004388	CTBS	0.0022907
7978644	NM_020529	NFKBIA	0.0023145
7971780	NM_002498	NEK3	0.0023234
7894008			0.0023268
8168762	NM_001325	CSTF2	0.0023304
8073081	NM_145298	APOBEC3F	0.0023305
8042119	NM_022894	PAPOLG	0.0023379
8147156	NM_007013	WWP1	0.0023385
8061471	NM_021067	GINS1	0.0023602

8113504	NM_004772	C5orf13	0.0023695
7952601	NM_001143820	ETS1	0.0024545
8174379	NM_002814	PSMD10	0.0024767
8055294	AK123815		0.0024768
8076436	NM_005650	TCF20	0.00248
8150318	NM_023110	FGFR1	0.0024883
7971208	NM_152903	KBTBD6	0.0024883
8098637	NM_207352	CYP4V2	0.0024984
8088745	NM_015123	FRMD4B	0.0025018
7922807	NM_015101	GLT25D2	0.0025086
8062545	NM_024855	ACTR5	0.002514
8173261	NM_001178032	ZC4H2	0.0025645
8067743	NM_025224	ZBTB46	0.0025724
8068786	ENST00000411330		0.0025848
8143387	NM_013446	MKRN1	0.0025915
8081645	NM_024616	C3orf52	0.0025916
8148304	NM_025195	TRIB1	0.0026029
8083457	NM_002886	RAP2B	0.0026084
8072360	NM_000355	TCN2	0.0026368
7958761	NM_001136538	ACAD10	0.0026402
8007363	NM_032387	WNK4	0.0026466
7986068	NM_000057	BLM	0.0026579
8009502	NM_000891	KCNJ2	0.0026886
7924476	NM_005681	TAF1A	0.002706
8100428	NM_004898	CLOCK	0.0027104
7947027	NM_001040697	UEVLD	0.0027376
7892958			0.0027538
8081362	NM_024548	CEP97	0.0027542
7904361	NM_017709	FAM46C	0.0027609
8103166	NM_001009555	SH3D19	0.0027689
8044605	NR_015377	LOC654433	0.0027855
8129666	NM_145176	SLC2A12	0.0027861
7965918	NM_001031701	NT5DC3	0.0027892
7923712	NM_005057	RBBP5	0.0027976
8082075	NM_138287	DTX3L	0.0028082
8116807	NM_152551	SNRNP48	0.0028095
7964677	NM_152440	C12orf66	0.0028146
7948125	NM_001004746	OR5T2	0.0028197
8023401	NM_025214	CCDC68	0.0028202
8001818	NM_004614	TK2	0.0028369
7913883	NM_000437	PAFAH2	0.0028394
8146579	NM_017780	CHD7	0.0028408
7970655	NM_004685	MTMR6	0.0028517
8167603	NM_001127899	CLCN5	0.0028524
7965855	NM_024057	NUP37	0.0028804
8046380	NM_000210	ITGA6	0.0028881
8131666	NM_002214	ITGB8	0.0028979
8099797	NM_001085399	RELL1	0.002904
8160900	NR_024481	FAM205B	0.0029122
7938834	NM_182964	NAV2	0.0029138
7936134	NM_024928	OBFC1	0.0029224
7892580			0.0029281
7893982			0.0029348
8065569	NM_138578	BCL2L1	0.0029388
7945377	NM_001135053	SIGIRR	0.0029554

7911941	NM_015557	CHD5	0.0030216
8106702	NM_032280	ZCCHC9	0.0030433
8163086	NM_032012	C9orf5	0.0030477
8074853	NM_080740	ZNF280A	0.0030723
8010212	NM_152468	TMC8	0.0031094
8155630	NM_015440	MTHFD1L	0.0031525
8161377	NM_015440	MTHFD1L	0.0031525
8052742	NM_001024680	FBXO48	0.0031541
7928944	NM_004670	PAPSS2	0.003161
8120967	NM_002526	NT5E	0.0031817
8124498	NR_002722	ZNF204P	0.003185
8017283	NR_026641	INTS2	0.0031941
7933982	NM_022129	PBLD	0.0031986
8147019	NM_016010	FAM164A	0.0031987
7892920			0.0032237
7986186	NM_033544	RCCD1	0.0032582
8078380	NM_001137674	ZNF860	0.003262
8025058	NM_004240	TRIP10	0.0032786
8098439	NM_002354	EPCAM	0.0032804
8066964	NM_199129	TMEM189	0.0032804
7936817	NM_024942	C10orf88	0.0032817
8163964	NM_005388	PDCL	0.0032883
7974835	NM_006255	PRKCH	0.0033027
7979378	NM_018168	C14orf105	0.0033063
8058552	NM_005896	IDH1	0.0033133
7928171	NM_003901	SGPL1	0.0033214
7987892	NM_022473	ZFP106	0.0033266
7965343	NM_172240	POC1B	0.0033679
8100688	NM_207407	TMPRSS11F	0.0034542
8171222	NM_001135565	HDHD1	0.0034592
7893665			0.0034689
7974689	NM_016651	DACT1	0.0034814
7960150	NM_003440	ZNF140	0.0034846
7892537			0.0034973
8060736	BC008667	PANK2	0.0035138
8116313	NM_001142306	C5orf60	0.0035309
8011275	NM_024086	METTL16	0.0035347
8121277	NM_001624	AIM1	0.0035353
8028227	NM_152604	ZNF383	0.0035422
7970455	NM_015974	CRYL1	0.0035564
8079021	NM_001904	CTNNB1	0.0035694
8106820	NM_006467	POLR3G	0.0035729
7984475	NM_006091	CORO2B	0.0035988
8168399	NM_001039840	CHIC1	0.0035999
8017651	NM_022739	SMURF2	0.0036065
7946142	NM_145040	PRKCDBP	0.0036406
8142912	NM_032842	TMEM209	0.0036909
8102720	NM_020337	ANKRD50	0.0036944
7997808	NR_024399	MGC23284	0.0037031
8095430	NM_000200	HTN3	0.0037127
7929168	NM_025235	TNKS2	0.0037397
7989501	NM_001218	CA12	0.0037649
8054004	NM_017789	SEMA4C	0.0037705
8145586	NM_018091	ELP3	0.0037745
7926661	NM_012228	MSRB2	0.0037809

8171684	NM_031892	SH3KBP1	0.0037813
8163637	NM_002160	TNC	0.0037863
7958895	NM_006187	OAS3	0.0037983
8094372	NM_003102	SOD3	0.0038207
7970509	ENST00000419579		0.0038223
7961182	NM_002260	KLRC2	0.0038409
7967967	ENST00000410794		0.0038462
8117054	NM_006366	CAP2	0.0038463
8109773	NM_001161661	WWC1	0.0038683
7974445			0.0038691
8022747	NM_004775	B4GALT6	0.0038726
8046685	NM_182678	UBE2E3	0.0038923
8035506	NM_004750	CRLF1	0.0038966
7895245			0.0039143
7892802			0.0039606
8103011	NM_199324		0.0039877
8148476	NM_014957	DENND3	0.0039952
8160546	NM_152570	LINGO2	0.0040099
8100109	NM_000807	GABRA2	0.0040127
8144880	NM_022071	SH2D4A	0.0040206
8143040	NM_032826	SLC35B4	0.0040451
7922250	NM_181093	SCYL3	0.0040555
7993756	NM_005622	ACSM3	0.0040816
7982290			0.0041063
8117165	NM_003107	SOX4	0.0041255
8153819	NM_138496	CYHR1	0.0041281
7892710			0.0041823
8162142	NM_030940	ISCA1	0.0042035
7982753	NM_152260	RPUSD2	0.0042181
8084169	ENST00000410241		0.0042239
8078412	NM_015442	CNOT10	0.0042242
7993453	NM_001128423	MPV17L	0.00423
7961173	AF461811	KLRC4-KLRK1	0.0042457
8146225	NM_001135674	C8orf40	0.0042591
7992877	NM_153028	ZNF75A	0.0042617
7893074			0.0042675
8060988	NM_014962	BTBD3	0.0043224
8178955	NM_004761	RGL2	0.0043921
8180144	NM_004761	RGL2	0.0043921
7981538	NM_002226	JAG2	0.004406
7970287	NM_005561	LAMP1	0.0044757
7919751	NM_021960	MCL1	0.0044874
8058477	NM_003709	KLF7	0.0045125
8094870	NM_001080505	SHISA3	0.0045815
8038347	NM_003598	TEAD2	0.0045986
7926821	NM_001172303	MASTL	0.0046016
7940869	NM_178443	FERMT3	0.0046056
7920057	NM_001083965	TDRKH	0.004611
7967624	NM_145648	SLC15A4	0.004619
8152131	ENST00000459507		0.0046398
8167163	ENST00000357412		0.0046517
8002381	NM_015386	COG4	0.004658
7934898	NM_144590	ANKRD22	0.004664
8115562	NM_144726	RNF145	0.0046694
8067955	NM_001338	CXADR	0.004673

7922689	NM_002065	GLUL	0.0046889
8035958	NM_032816	CEP89	0.0046956
7906930	NM_145697	NUF2	0.0047217
8123259	NM_000301	PLG	0.004739
8007931	NM_000212	ITGB3	0.0047542
8113286	NM_018343	RIOK2	0.0047649
7893860			0.0048036
8068593	NM_005239	ETS2	0.0048275
8109830	NM_017785	CCDC99	0.004833
8004532			0.0048427
7917037	NM_001130042	CRYZ	0.0048619
8039635	NM_001012729	DUXA	0.0048716
7987369	NM_080650	ATPBD4	0.0048733
7902425	NM_152996	ST6GALNAC3	0.0049267
7954559	NM_003622	PPFIBP1	0.0049288
8150186	NM_024787	RNF122	0.0049293
7958620	NM_014055	IFT81	0.004981
8162421	NM_022755	IPPK	0.0049859
8069122	NM_030891	LRRC3	0.0049901
8145894	NM_004674	ASH2L	0.0050018
8025478	NM_032497	ZNF559	0.0050115
7895995			0.0050348
7927169	NR_026693	ZNF487P	0.0050476
8157941	NM_001099270	ZBTB34	0.0050482
8095380	NM_014058	TMPRSS11E	0.0050553
7972650	NM_175929	FGF14	0.0051148
7991224	NM_178232	HAPLN3	0.0051238
8021376	NM_001144967	NEDD4L	0.0051307
7967240	NM_022916	VPS33A	0.0051313
8106429	NM_018046	AGGF1	0.0051362
8075776			0.0051587
7960143	NM_003428	ZNF84	0.0052078
8125687	NM_004761	RGL2	0.0052297
8120600	ENST00000384451		0.005235
8149955	NM_018492	PBK	0.0052405
8005839	NM_014573	TMEM97	0.0052802
8008113	NM_005831	CALCOCO2	0.0053012
7997179	NM_001361	DHODH	0.0053235
8122634	NM_001030060	SAMD5	0.0053269
7992293	NM_024600	TMEM204	0.0053342
7916862	NM_024911	WLS	0.005358
7944033	ENST00000459137		0.0053959
7895952			0.0054233
8026503	NR_024335	FLJ25328	0.0054844
7932796	NM_021738	SVIL	0.0054876
8083090	NM_001080412	ZBTB38	0.0055431
8142981	NM_001018111	PODXL	0.0055452
8138689	NM_003930	SKAP2	0.0055803
7905677	NM_130898	CREB3L4	0.0056322
8055350	NM_030923	TMEM163	0.0056689
8046520	ENST00000390865		0.0056702
8083673	NM_001042706	IQCJ	0.0056975
8089606	NM_018338	WDR52	0.0057174
8177628	NM_176816	CCDC125	0.0057209
7896686			0.0057551

8157233	NM_032303	HSDL2	0.0057559
8157947	NM_001099270	ZBTB34	0.0057579
7992396	NM_031208	FAHD1	0.0057581
7893019			0.0057639
7897089	NM_014638	PLCH2	0.0057669
8116494	NM_152283	ZFP62	0.0058384
8058509	NM_001080475	PLEKHM3	0.0058597
7977127	NM_001130107	KLC1	0.0058744
7969626	NM_180989	GPR180	0.0059107
8076757	NM_014246	CELSR1	0.0059648
8060772	NM_003818	CDS2	0.0059748
8128726	NM_173672	PPIL6	0.0059769
8116848	NM_017906	PAK1IP1	0.0059848
7921088	NM_006617	NES	0.0059945
8059650	NM_080424	SP110	0.0060026
8170971	NM_001363	DKC1	0.0060104
7960865	NM_006931	SLC2A3	0.0060491
8115895	NM_020444	KIAA1191	0.0060638
8120698	NM_133645	MTO1	0.0060666
7903308	NM_019083	CCDC76	0.0060671
8117714	NM_001005216	OR2J3	0.0060718
8046428	NM_007023	RAPGEF4	0.0060802
8032312	NM_138813	ATP8B3	0.0060869
8061129	BC016869	C20orf72	0.0061008
8016088	NM_144609	CCDC43	0.00611
8052845	NM_022173	TIA1	0.0061193
7901219	NM_199044	NSUN4	0.0061252
8161224	NM_014872	ZBTB5	0.006149
7974214	NM_172193	KLHDC1	0.0061499
8157582	NM_000177	GSN	0.0061895
8084818	NM_178335	CCDC50	0.0061944
7923086	NM_018136	ASPM	0.0062051
8058373	NM_018256	WDR12	0.0062307
7962918	NM_003394	WNT10B	0.006261
8151747	NM_001008495	TMEM64	0.0062655
7922268	NM_014970	KIFAP3	0.0063106
7964759	NM_021150	GRIP1	0.0063193
8058258	NM_152388	TMEM237	0.0063299
7909708	NM_016343	CENPF	0.0063396
7917359	NM_017953	ZNHIT6	0.0063801
7977046	NM_006291	TNFAIP2	0.0064071
8140859	NM_006980	MTERF	0.0064367
8095364	NM_014058	TMPRSS11E	0.0064465
8170027	NM_182540	DDX26B	0.0064505
7999023	ENST00000411274		0.0064633
8077513	NM_015453	THUMPD3	0.006473
8059279	NM_004438	EPHA4	0.0064826
7896271			0.0065076
8129706	NM_006620	HBS1L	0.0065224
7982564	NM_152594	SPRED1	0.0065365
7894678			0.0065418
8045736	NM_052905	FMNL2	0.0065518
7898939	NM_020448	NIPAL3	0.0065658
7903407	NM_020978	AMY2B	0.0065925
8022338	NM_145290	GPR125	0.0065989

7938370	NM_015055	SWAP70	0.0066096
8045776	NM_052917	GALNT13	0.0066336
8166585	AK057304	FLJ32742	0.0066459
7957890	NM_014503	UTP20	0.0067421
8097335	NM_014278	HSPA4L	0.006772
7919940	NM_005997	VPS72	0.0067847
8173269	NM_031206	LAS1L	0.0069224
7903032	NM_007358	MTF2	0.0069689
8029368	NM_001037813	ZNF284	0.0069806
8112803	NM_005779	LHFPL2	0.0070347
7897824	NM_014874	MFN2	0.0070468
8046861	NM_002210	ITGAV	0.0071225
8114193	NM_001033503	SAR1B	0.0071271
7958379	NM_007062	PWP1	0.0071315
8102751	NM_144643	SCLT1	0.0071916
8139656	NM_001001555	GRB10	0.0072282
8107458	NM_016144	COMMD10	0.007238
8020267	NM_032142	CEP192	0.007328
7892761			0.0073557
8142061	NM_019042	PUS7	0.007365
7894306			0.0074316
7894710			0.0074448
8069620	NM_080794	MRPL39	0.007448
8008922	NM_003620	PPM1D	0.0074599
7993248	NM_152308	RMI2	0.0074871
8036351	BC052603	ZNF850	0.0075119
8122265	NM_006290	TNFAIP3	0.0075232
7895318			0.0075329
8116247	NM_005649	ZNF354A	0.0075402
7917976	NM_194292	SASS6	0.0075617
8072461	NM_016733	LIMK2	0.0075721
8068460	NM_015358	MORC3	0.0075733
8112342	NM_197941	ADAMTS6	0.007585
8148302	ENST00000410881		0.007603
7969768	ENST00000411195		0.0076093
7913869	NM_203401	STMN1	0.0076124
8098291	NM_173872	CLCN3	0.0076361
8167441	ENST00000384209		0.0076401
7931081	NM_021622	PLEKHA1	0.0076525
7895003			0.0076807
8051443	NM_003162	STRN	0.0077077
7909898	NM_198551	MIA3	0.0077366
8100306			0.0077707
7954789	NM_001013620	ALG10B	0.0078224
8040090	NM_014746	RNF144A	0.0078364
7963567	NM_002273	KRT8	0.0078445
8160478	NM_024828	C9orf82	0.0078733
7969881	NM_003291	TPP2	0.0079042
7964271	NM_000946	PRIM1	0.0079271
8153430	NM_145201	NAPRT1	0.0079349
8035236	NM_033417	HAUS8	0.0079454
8109677	NM_198904	GABRG2	0.007949
8178324	AF032109	ZNRD1-AS1	0.0079891
8003850	NM_144611	CYB5D2	0.0079922
8177700	NM_013936	OR12D2	0.0080209

7994235	ENST00000426719		0.0081239
8099982	NM_004307	APBB2	0.008134
7996393	NM_001755	CBFB	0.0081428
8027233	NM_033204	ZNF101	0.0081462
8039928	AB096683	FAM72D	0.0081638
7959408	NM_014708	KNTC1	0.0081639
8167625	NM_033031	CCNB3	0.0081732
7907171	NM_003666	BLZF1	0.0082179
7921970	NM_000696	ALDH9A1	0.0082194
7963157	NM_013277	RACGAP1	0.0082222
7969390	NM_006346	PIBF1	0.008241
8044450	NM_198581	ZC3H6	0.0082416
7894657			0.0082435
8017133	NM_182620	SKA2	0.0083469
8117718	NM_001005216	OR2J3	0.0083532
8051215	NM_022128	RBKS	0.0083598
7895311			0.0083657
8102450	NM_022569	NDST4	0.0083761
8047288	NM_152524	SGOL2	0.0083832
8005102	ENST00000363567		0.0083999
8128716	NM_006016	CD164	0.0084294
8095163	NM_001024924	EXOC1	0.0084316
8166747	NM_138780	SYTL5	0.0084399
8031750	NM_001098491	ZNF419	0.0084503
8124262	NM_016614	TDP2	0.0084695
8145454	NM_004331	BNIP3L	0.0085347
8080994	NM_001124759	FRG2C	0.0085553
8063478	NM_080821	C20orf108	0.0085719
8020930	NM_018255	ELP2	0.0085786
8091402	NM_138786	TM4SF18	0.0085795
8044880	ENST00000459555		0.0085829
8052872	NM_003236	TGFA	0.0086218
8044473	NM_019014	POLR1B	0.0086466
8054517	AK095678	LOC151009	0.0086562
8126860	NM_000255	MUT	0.0086738
7930422	NM_005445	SMC3	0.008678
7894717			0.0087078
8089627	NM_144718	SPICE1	0.0087117
7904452	AB096683	FAM72D	0.0087364
7991562			0.0087445
8121525	NM_153369	KIAA1919	0.0087616
8093336	NM_003441	ZNF141	0.0087832
7896004			0.0087856
7895728			0.0087922
7934145	NM_207119	LRRC20	0.0087945
7985192	NM_001013619	AGPHD1	0.0088075
7978760	NM_017658	KLHL28	0.0088092
8113130	NM_024717	MCTP1	0.0088455
7896025			0.0088646
8087224	NM_000387	SLC25A20	0.0088669
7957850	NM_174942	GAS2L3	0.0088751
8008991	ENST00000363972		0.0088789
8112615	NM_003633	ENC1	0.0088916
7917052	NM_152697	SLC44A5	0.008909
7910381	AK055963	DUSP5P	0.0089433

7894577			0.0089472
8119241	ENST00000363068		0.0089529
7904211	NM_138959	VANGL1	0.0089789
7905220	NM_004425	ECM1	0.0090038
8173349	NM_001002254	AWAT2	0.0090071
8084947	NM_001105573	FBXO45	0.0091411
8031720	NM_213598	ZNF543	0.0091445
8140752	NM_000443	ABCB4	0.0091505
8149275			0.0091539
7892557			0.009158
7892507			0.0091702
8081548	NM_015480	PVRL3	0.0092031
7896319			0.0092476
8076185	NM_175709	CBX7	0.0093062
7913415	NM_032236	USP48	0.0093408
8081256	NM_018309	TBC1D23	0.0093568
8110237	NM_133369	UNC5A	0.0093797
8002882	NM_021615	CHST6	0.0094104
7965322	NM_000899	KITLG	0.0094266
8122279	NM_020340	KIAA1244	0.0094271
8121927	NM_030963	RNF146	0.0094357
8143441	NM_001080392	KIAA1147	0.0094722
8173892	NM_000390	CHM	0.0094743
7895194			0.0095014
8121622	NM_001010892	RSPH4A	0.0095283
8027368	NM_203282	ZNF254	0.0095422
8116722	AY358807	LOC100129033	0.0095854
8163116	NM_019114	EPB41L4B	0.0095896
8013179	NM_145691	ATPAF2	0.0096039
8129482	NM_001017373	SAMD3	0.0096377
7955055	NM_017842	SLC48A1	0.0096568
7902074	NM_002303	LEPR	0.009675
7982712	NM_033286	C15orf23	0.0096815
8180374	NM_001006666	APOBEC3F	0.0097068
8174340	NM_018301	RBM41	0.0097392
8162601	NM_153695	ZNF367	0.0097546
8002904	NM_012091	ADAT1	0.0098054
8147040	NM_001105539	ZBTB10	0.0098154
8002523	NM_018348	FTSJD1	0.0098927
7912537	NM_004753	DHRS3	0.0099024
8144184	NM_020728	ESYT2	0.0099294
8003204	NM_016095	GINS2	0.0099361
8175319	NM_007131	ZNF75D	0.0099512
8163825	NM_005658	TRAF1	0.0099647
7999279	NM_016256	NAGPA	0.0099873
8029688	NM_012099	CD3EAP	0.009995

Down-regulated gene features following siRNA-mediated *LAPTM4B* knockdown

Probeset	Accession	Symbol	p-value
8147503	NM_018407	LAPTM4B	0
8150978	NM_004056	CA8	0.0000005

8111677	NM_002310	LIFR	0.0000005
7919324	ENST00000384480		0.0000007
8045088	ENST00000363562		0.000001
7969665	NM_153456	HS6ST3	0.0000014
8040742	NM_012326	MAPRE3	0.0000017
7922976	NM_000963	PTGS2	0.0000018
8102800	NM_014331	SLC7A11	0.000002
8136159	ENST00000362646		0.0000021
7982597	NM_003246	THBS1	0.0000023
7947512	NM_015430	PAMR1	0.0000024
7944333	ENST00000364665		0.0000028
8021081	NM_001128588	SLC14A1	0.0000029
7902452	NM_174858	AK5	0.000004
8180376	NM_001353	AKR1C1	0.0000043
7914342	NM_004102	FABP3	0.0000052
7938702	NR_026750		0.000006
7977933	NM_012244	SLC7A8	0.0000063
8112731	NM_004101	F2RL2	0.0000071
8058857	NM_000599	IGFBP5	0.0000082
8017843	NM_001174166	SLC16A6	0.0000111
7951372	NM_033306	CASP4	0.0000116
7928429	NM_002658	PLAU	0.0000118
7989985	NM_001004439	ITGA11	0.0000124
7893309			0.0000131
8092169	NM_003810	TNFSF10	0.0000146
8080964	NM_001080393	GXYLT2	0.0000155
8126658	NM_178148	SLC35B2	0.0000168
7943998	NM_006169	NNMT	0.0000173
8101429	NM_016619	PLAC8	0.0000191
7898809	NM_017449	EPHB2	0.0000194
7926545	NM_032812	PLXDC2	0.0000195
8109093	NM_014945	ABLIM3	0.0000224
7950042	NM_012309	SHANK2	0.0000242
8157038	NM_080546	SLC44A1	0.0000251
8008598	NM_001102402	PCTP	0.0000262
8165911	NM_005647	TBL1X	0.0000265
8102728	GENSCAN00000031902		0.000027
7953021	NM_024551	ADIPOR2	0.0000278
8101675	NM_004827	ABCG2	0.0000284
8021540	ENST00000362997		0.0000297
7980485	NM_013989	DIO2	0.0000301
8072626	NM_000362	TIMP3	0.0000304
7966035	NM_006825	CKAP4	0.000031
7906061	NM_152280	SYT11	0.0000323
8020141	NM_153000	APCDD1	0.0000351
7920642	NM_001018016	MUC1	0.0000353
8164580	NM_004878	PTGES	0.0000356
8078260	ENST00000384001		0.000037
8091537	NM_178822	IGSF10	0.000039
8151369	NM_153225	C8orf84	0.0000415
8098204	NM_001873	CPE	0.0000453
8046333	NM_024843	CYBRD1	0.0000479
7922887	ENST00000383913		0.0000508
7920278	NM_002960	S100A3	0.0000556
8150698	NM_003068	SNAI2	0.0000562

7984932	NM_138967	SCAMP5	0.0000569
8168470	NM_001866	COX7B	0.0000585
8024299	NM_001018	RPS15	0.0000597
8119088	NM_078467	CDKN1A	0.0000603
8046824	AK092099	FSIP2	0.0000616
8014063	NM_006495	EVI2B	0.0000622
8065444	NM_032501	ACSS1	0.000064
8115847	ENST00000364407		0.0000729
7925457	NM_002924	RGS7	0.0000735
7893184			0.0000759
7948995	ENST00000398868		0.0000781
8176133	NM_000402	G6PD	0.0000788
7995681	NM_004530	MMP2	0.0000812
7963187	NM_001113546	LIMA1	0.0000862
8068022	NR_030784	MIR155	0.0000882
7902104	NM_002600	PDE4B	0.0000898
8009639	NM_022036	GPRC5C	0.0001025
7892945			0.0001036
8094911	NM_020453	ATP10D	0.0001058
8059413	NM_014689	DOCK10	0.0001074
8155246	ENST00000363632		0.0001078
8075635	NM_000362	TIMP3	0.0001112
8143188	NM_194071	CREB3L2	0.0001235
8099721	NM_015187	SEL1L3	0.0001243
7921806	NM_003779	B4GALT3	0.0001245
7894266			0.0001256
8138735	NM_019102	HOXA5	0.0001304
7976560	NM_000623	BDKRB2	0.0001324
7949067	NM_004322	BAD	0.0001346
8116418	NM_005110	GFPT2	0.0001375
8078350	NM_001024847	TGFBR2	0.0001379
8090214	NM_024628	SLC12A8	0.0001388
8120043	NM_001024630	RUNX2	0.0001431
8136388	NM_018295	TMEM140	0.0001471
7925929	NM_003739	AKR1C3	0.0001472
8112139	NM_002184	IL6ST	0.0001485
8063590	NM_002591	PCK1	0.0001496
7964484	NM_001478	B4GALNT1	0.0001538
8040190	NM_198182	GRHL1	0.000156
8090433	NM_007283	MGLL	0.0001567
7942596	NM_001235	SERPINH1	0.0001592
7895706			0.0001605
8114814	NM_000176	NR3C1	0.0001664
8149500	NM_001001924	MTUS1	0.0001684
7946401	NM_005418	ST5	0.0001687
8051583	NM_000104	CYP1B1	0.0001779
8017927	NM_080283	ABCA9	0.0001795
8054377	NM_201555	FHL2	0.0001805
8128956	NM_002037	FYN	0.0002008
7966026	NM_014840	NUAK1	0.0002015
8150592	NM_005195	CEBPD	0.0002029
7986293	NM_018349	MCTP2	0.0002055
7970676	NM_001007538	SHISA2	0.0002066
7978544	NM_022073	EGLN3	0.0002116
8171577	ENST00000384298		0.0002148

7892576			0.0002154
8112855			0.0002236
7997491	NM_002153	HSD17B2	0.0002252
8088476			0.0002281
7894844			0.000231
8090690	NM_130808	CPNE4	0.0002316
7965403	NM_002345	LUM	0.0002377
7961371	NM_030640	DUSP16	0.0002379
8045336	NM_001508	GPR39	0.0002383
7918379	NM_000849	GSTM3	0.0002388
7958950	BC022092	C12orf52	0.0002394
8012218	ENST00000364619		0.0002437
8054364	NM_004257	TGFBRAP1	0.0002478
8019376	NM_022156	DUS1L	0.0002486
8004266	NM_201566	SLC16A13	0.0002506
7997726	NM_001451	FOXF1	0.0002509
8099850	NM_024943	TMEM156	0.000251
8163257	NM_057159	LPAR1	0.0002512
7897044	NM_002744	PRKCZ	0.0002529
7948741	NM_012200	B3GAT3	0.0002544
8056763	ENST00000433675		0.0002616
8064100	NM_024299	PPDPF	0.0002623
8046646	NM_032523	OSBPL6	0.0002657
8005132	NR_002211	MEIS3P1	0.0002667
7933933	NM_021800	DNAJC12	0.0002714
7949503	NM_016938	EFEMP2	0.0002753
8070574	NM_005423	TFF2	0.0002782
7892680			0.0002804
8180322	NM_181619	KRTAP21-1	0.0002827
8081375	NM_145037	FAM55C	0.0002851
8122365	NM_020455	GPR126	0.0002876
8165682	NC_001807		0.0002884
8090193	NM_020733	HEG1	0.000291
8151927	ENST00000362862		0.0002932
8099037	NM_001146069	MFSD10	0.0002935
7895880			0.0002999
8102482	NM_014822	SEC24D	0.0003017
8116921	NM_001955	EDN1	0.0003048
8069764	ENST00000365114		0.0003064
8161265	NM_001007563	IGFBPL1	0.0003093
8119974	NM_001078175	SLC29A1	0.0003115
7992895	NM_017810	ZNF434	0.0003158
7933772	NM_020987	ANK3	0.0003188
7929779	NM_000392	ABCC2	0.0003232
7926105	NM_001002295	GATA3	0.0003296
8092691	NM_001706	BCL6	0.000346
8011193	NR_028504	MIR22HG	0.0003483
8025402	NM_139314	ANGPTL4	0.0003493
8078066	NM_152536	FGD5	0.0003535
8166127	NM_002063	GLRA2	0.0003542
8135856	NM_001662	ARF5	0.0003688
7931353	NM_006504	PTPRE	0.0003699
7898693	NM_000478	ALPL	0.0003756
8155508			0.0003757
7947358	NM_002901	RCN1	0.0003779

8114938	NM_014790	JAKMIP2	0.0003814
8145669	NM_001008711	RBPMS	0.0003859
8110114	NM_006650	CPLX2	0.0004027
7894563			0.0004106
7917942	AK092728	MIR137HG	0.0004318
7892836			0.000436
8019964	NM_001010000	ARHGAP28	0.000437
8029854	NM_005628	SLC1A5	0.0004425
8163185	NM_003329	TXN	0.0004444
8096301	NM_001040058	SPP1	0.000455
8162283	NM_004560	ROR2	0.0004552
8088180	NM_003392	WNT5A	0.0004556
8148917	NM_138431	MFSD3	0.0004784
7893843			0.000484
8056303	NM_033272	KCNH7	0.000485
8063549			0.0004954
8077441	NM_003670	BHLHE40	0.0004984
8081564	NM_198196	CD96	0.0005015
8059186	NM_002846	PTPRN	0.0005024
8085797	NM_001128176	THRB	0.0005065
8018814	ENST00000410876		0.0005092
8127107			0.0005166
8174664	ENST00000363421		0.0005196
7982938	NM_006293	TYRO3	0.0005257
7996761	NM_012320	PLA2G15	0.0005273
7971369	NM_198404	KCTD4	0.0005276
7923608	NM_000537	REN	0.0005293
7937900	NM_003156	STIM1	0.0005464
8016018	NM_001143780	SLC25A39	0.0005491
7917649	NM_003243	TGFBR3	0.0005521
8143307	NM_022740	HIPK2	0.0005544
8107671			0.0005546
8129985	NM_006718	PLAGL1	0.000555
7997933	NM_033251	RPL13	0.0005618
8035304	NM_004335	BST2	0.000563
8175871	NM_000425	L1CAM	0.0005735
8166948	ENST00000363832		0.0005742
8095585	NM_001098484	SLC4A4	0.0005756
8109528	NM_001037332	CYFIP2	0.0005879
8152812	NM_174911	FAM84B	0.0005879
8015445	NR_033465	NT5C3L	0.0005901
8151587	NM_001099670	C8orf59	0.0006099
7969204	NM_052950	WDFY2	0.000613
8114050	NM_015146	8-Sep	0.0006147
8144802	NM_006207	PDGFRL	0.0006177
8115543	NM_024007	EBF1	0.0006215
7902687	NM_001554	CYR61	0.0006378
7983228	NM_002373	MAP1A	0.0006409
7896013			0.0006432
7895267			0.0006444
7990674	NM_006383	CIB2	0.0006513
7971692	NM_012141	INTS6	0.0006541
8114083	NM_014423	AFF4	0.0006584
7974255	ENST00000364619		0.0006626
8178090	NM_001040437	C6orf48	0.0006719

8179326	NM_001040437	C6orf48	0.0006719
7951108	AF364863		0.0006776
8003298	NM_003486	SLC7A5	0.0006857
8121275	ENST00000363444		0.0006897
8122222	NM_018945	PDE7B	0.0006987
8105067	NM_000958	PTGER4	0.0007011
7922337	NM_005092	TNFSF18	0.0007065
7917530	AL832451		0.000707
8145293	NM_014265	ADAM28	0.0007199
8042788	NM_001615	ACTG2	0.0007246
8145977	NM_021623	PLEKHA2	0.0007285
8032480	NM_016199	LSM7	0.0007297
8101762	NM_000345	SNCA	0.0007305
7976322	NM_001275	CHGA	0.0007314
7911339	NR_031741		0.0007423
8165698	NR_031741		0.0007423
8080781	NM_017771	PXK	0.0007439
7971461	NM_002298	LCP1	0.0007472
8158406	NM_018201	TBC1D13	0.0007682
8040036	NM_001011	RPS7	0.0007713
8152703	NM_058229	FBXO32	0.0007869
8004905	NM_153210	USP43	0.0008004
7999360	NM_000982	RPL21	0.0008051
7893822			0.0008083
7913249	NM_032409	PINK1	0.0008108
8090469	NM_032638	GATA2	0.0008148
8045664	NM_177964	LYPD6B	0.0008246
7965941	NM_031302	GLT8D2	0.0008336
8036143	NM_001040425	U2AF1L4	0.0008364
8031076	NM_031896	CACNG7	0.0008418
8047854	NM_001142300	CCNYL1	0.0008524
7946661	NM_015881	DKK3	0.000854
8023889	NM_001025101	MBP	0.0008725
7952341	NM_024769	CLMP	0.0008784
8069880	NM_003253	TIAM1	0.000891
8140113	NM_004603	STX1A	0.0008914
8127854	NM_002395	ME1	0.0008987
8095299	ENST00000364806		0.0009161
8169389	NM_002578	PAK3	0.0009197
7909603	NM_013349	NENF	0.0009209
7924388	NM_006085	BPNT1	0.0009233
8043114	NM_031283	TCF7L1	0.0009264
8170775	NM_006280	SSR4	0.0009306
7945321	NM_138342	GLB1L2	0.0009396
8027402	NM_001238	CCNE1	0.0009539
8131661	NM_000982	RPL21	0.0009582
8004867	NM_001025579	NDEL1	0.0009829
7963142	NM_012306	FAIM2	0.0009836
7936727	NM_000982	RPL21	0.0009899
8132031	NM_175887	PRR15	0.0010034
7916493	NM_003713	PPAP2B	0.0010043
8091954	NM_014498	GOLIM4	0.0010223
8167103	NM_006201	CDK16	0.0010336
7970831	NM_007106	UBL3	0.0010445
8148040	NM_052886	MAL2	0.0010494

7894061			0.0010499
8093950	NM_005980	S100P	0.0010544
7914021	NM_003047	SLC9A1	0.0010545
8118945	NM_001171818	PPARD	0.0010725
8022295	NM_022068	PIEZO2	0.0010792
8119898	NM_001025366	VEGFA	0.0010861
8054870	ENST00000364714		0.0010973
7968226	NM_000982	RPL21	0.0010984
8159127	NM_002957	RXRA	0.0011033
7914139	NM_014110	PPP1R8	0.0011051
7912374	NM_003132	SRM	0.0011106
8159379	NM_032928	TMEM141	0.0011208
8062206	NM_080748	ROMO1	0.0011271
8133459	NM_003388	CLIP2	0.001135
8075462	NM_080430	SELM	0.0011399
7955469	NM_001039960	SLC4A8	0.0011434
7972239	NM_032229	SLITRK6	0.0011467
7964460	NM_004083	DDIT3	0.0011473
7924144	NR_026911	RPL21P28	0.001149
7993680	BC050464	C16orf62	0.0011528
8164649	NM_013318	PRRC2B	0.0011566
8063536	NM_003222	TFAP2C	0.0011593
7963353	NM_002281	KRT81	0.0011638
8049670	NM_002081	GPC1	0.0011661
8022310	NM_022068	PIEZO2	0.0011816
8173522	ENST00000410980		0.0011818
8124402	NM_005323	HIST1H1T	0.0011947
7928308	NM_019058	DDIT4	0.0011993
8019521	NM_004514	FOXK2	0.0012256
8104663	NM_004932	CDH6	0.0012392
7979085	NM_002863	PYGL	0.0012399
8162610	NM_033331	CDC14B	0.0012474
8126371	NM_001760	CCND3	0.0012513
8007607	NM_006695	RUNDC3A	0.0012743
8172317	NM_006950	SYN1	0.001286
8062108	NM_006404	PROCR	0.0012883
8086899	NM_005793	NME6	0.0012887
8086961	NM_004567	PFKFB4	0.0012924
8044353	NM_001142807	ACOXL	0.0012946
8131205	NM_152744	SDK1	0.0013026
8101210	ENST00000429255		0.0013134
8074842	NM_001130111	FAM108A1	0.0013257
7972297	NM_005845	ABCC4	0.001344
7913252	NM_032409	PINK1	0.0013803
8152297	NM_001146	ANGPT1	0.0013805
7893455			0.0014059
7936028	NM_005736	ACTR1A	0.0014069
8040458	NM_002252	KCNS3	0.0014106
7963134	NM_001037806	NCKAP5L	0.0014112
8102678	NM_001130698	TRPC3	0.0014131
8053325	BC024248		0.0014145
7950307	NM_003355	UCP2	0.0014181
7894906			0.0014307
8122136	NM_001016	RPS12	0.0014459
7953128	ENST00000410561		0.0014539

7895256			0.0014632
7941843	NM_017857	SSH3	0.0014856
8056005	NM_001105	ACVR1	0.0014982
7895229			0.0014993
8164833	NM_021996	GBGT1	0.0015033
8168045	NM_004429	EFNB1	0.0015094
8131965	AK056484	LOC441204	0.00152
7895455			0.0015225
8119161	NM_002648	PIM1	0.0015287
7904959			0.0015304
8138708	NM_005522	HOXA1	0.0015308
7950370	ENST00000363779		0.0015309
7904048	ENST00000459345		0.0015482
8165552	NM_001130969	NELF	0.0015537
8095508	NM_212557	AMTN	0.0015612
7960359	ENST00000447487		0.0015822
8012304	NM_021210	TRAPPC1	0.0015911
7895910			0.0016017
8106532	ENST00000384661		0.0016057
8101716	ENST00000384749		0.0016058
8065817	NM_000178	GSS	0.00161
8138258	NM_001135924	VWDE	0.0016166
8161852	ENST00000363171		0.0016249
8003060	NM_145168	SDR42E1	0.0016308
8111887	NM_001005473	PLCXD3	0.0016628
7945663	NM_001170820	IFITM10	0.0016642
7895640			0.001672
8108822	NM_173828	RELL2	0.0016742
8017964	NM_080284	ABCA6	0.0017145
8041781	NM_001430	EPAS1	0.0017236
8005475	NM_001037330	TRIM16L	0.001725
7923453	NM_006618	KDM5B	0.001735
7938100	NM_000543	SMPD1	0.0017433
8045499	NM_006895	HNMT	0.0017468
7893058			0.0017483
7937335	NM_003641	IFITM1	0.0017795
7904293	NM_020440	PTGFRN	0.0017861
8047248	NM_006226	PLCL1	0.0017977
8071809	NM_000854	GSTT2	0.0017994
8074962	NM_000854	GSTT2	0.0017994
7896369			0.0018232
8097586	NM_207123	GAB1	0.0018334
8001782	NM_002954	RPS27A	0.0018396
7963313	NM_007210	GALNT6	0.0018519
8023822	NM_182511	CBLN2	0.0018526
7931832	NM_001354	AKR1C2	0.0018571
7935521	NM_021732	AVPI1	0.0018715
7914630	NM_018207	TRIM62	0.0018944
8170390	NM_000202	IDS	0.0019064
7953390	NM_001039916	ZNF384	0.0019109
8152750	NM_194291	TMEM65	0.0019277
7938390	NM_001124	ADM	0.0019348
8147721	AK127183	FLJ45248	0.0019393
8140534	NM_006379	SEMA3C	0.0019534
8133670	NM_000941	POR	0.0019595

8125125	NM_021177	LSM2	0.0019932
8178641	NM_021177	LSM2	0.0019932
8179839	NM_021177	LSM2	0.0019932
7920912	NM_020131	UBQLN4	0.0020113
7987145	NM_001103184	FMN1	0.0020114
7892643			0.0020133
8108359	NM_016606	REEP2	0.0020158
7984771	NM_005576	LOXL1	0.0020243
7956741			0.002025
8005661	NM_001033553	SPECC1	0.0020326
7895721			0.0020502
7917199	NM_024686	TTLL7	0.0020622
8096765	NM_033625	RPL34	0.0020742
8138277	NM_001135924	VWDE	0.0020765
7951896	NM_004716	PCSK7	0.0020815
8002057	NM_001082486	ACD	0.0020851
8174239	NM_032621	BEX2	0.0020937
8029773	NM_001002915	IGFL2	0.0020938
8175593	NM_000202	IDS	0.0020971
7939613	NM_005456	MAPK8IP1	0.0021205
7894628			0.0021249
7910111	NM_000120	EPHX1	0.0021284
8104609	ENST00000327611		0.0021323
8083707	NM_001168214	C3orf80	0.0021331
7927803	NM_178011	LRRTM3	0.002146
8028563	NM_021107	MRPS12	0.002162
8086981	NM_033199	UCN2	0.002163
7965964	NM_032148	SLC41A2	0.0021711
8042052	NM_002954	RPS27A	0.0021904
8077612	NM_001025930	TTLL3	0.0021992
7904843	NM_002614	PDZK1	0.0022002
7925062	NM_020808	SIPA1L2	0.0022055
8064557	AK293638	FAM113A	0.0022125
8141560	NM_003227	TFR2	0.0022126
8029969	NM_003009	SEPW1	0.0022172
8102982	BC064848	GAB1	0.0022266
8052581			0.0022571
8067380	NM_144703	LSM14B	0.0022586
7894512			0.0022662
7894974			0.0022785
8010036	NM_180990	ZACN	0.0022889
8158204	NM_001040011	SWI5	0.0022889
7942796	NM_001029859	KCTD21	0.0022929
8065693	NM_031232	NECAB3	0.002294
8036045	NM_175872	ZNF792	0.0023026
7896529			0.0023037
8168357	NR_002309	RPS26P11	0.0023136
7915659	NM_024602	HECTD3	0.0023213
8115099	NM_002609	PDGFRB	0.0023262
8115327	NM_003118	SPARC	0.0023315
8124689	NR_002139	HCG4	0.0023392
8081454	ENST00000363491		0.0023406
7978824	NM_001030001	RPS29	0.0023562
7982514	ENST00000384363		0.002357
8112841	NM_004272	HOMER1	0.002358

7956114	NM_001029	RPS26	0.0023698
8175269	NM_001166599	FAM122B	0.0023714
7893821			0.002404
8168843	NM_021029	RPL36A	0.002428
7895198			0.0024435
8005134	NM_000676	ADORA2B	0.0024446
8040467	ENST00000363893		0.0024809
7957271	ENST00000361500		0.0024813
8140371	NM_031925	TMEM120A	0.002501
8056491	NM_002977	SCN9A	0.0025064
8002303	NM_000903	NQO1	0.0025109
8104731	AF090909		0.0025118
7916491			0.0025152
8084016	NM_006218	PIK3CA	0.0025167
8104727	NM_019059	TOMM7	0.0025178
8013833	NM_178860	SEZ6	0.0025325
7940479	NM_016499	TMEM216	0.0025391
8178676	NM_000434	NEU1	0.0025554
7894504			0.0025879
7936798	NM_022034	CUZD1	0.002611
7896722			0.0026265
8125139	NM_000434	NEU1	0.0026283
8179851	NM_000434	NEU1	0.0026283
8001449	NM_024336	IRX3	0.0026378
7962559	NM_018018	SLC38A4	0.0026554
8038785	NM_001163922	VSIG10L	0.0026628
7894978			0.0026636
7895832			0.0026729
7942674	NM_015516	TSKU	0.0026739
8003840	NM_001014764	TMEM93	0.0026771
7963986	NM_002870	RAB13	0.0026825
7956220	NM_024068	OBFC2B	0.0027028
8008016	NM_014726	TBKBP1	0.0027333
8041206	NM_030915	LBH	0.0027576
8030630	NM_007121	NR1H2	0.0027645
7894073			0.0027788
8077099	NM_005138	SCO2	0.0028129
8084630	BX640843		0.0028435
8046833	AK126104	FSIP2	0.0028437
7917739			0.0028504
8152491	NM_000127	EXT1	0.0028539
8087236	NM_001009996	DALRD3	0.0028555
7895656			0.0028787
7924069	ENST00000364102		0.0028792
8112738	ENST00000410127		0.0028956
7902043	NM_014787	DNAJC6	0.0028961
7985285	NM_014862	ARNT2	0.0029042
8094101	NM_001014448	CPZ	0.0029044
8136095	NM_015328	AHCYL2	0.0029085
7953626	NM_014718	CLSTN3	0.0029225
8063386	NM_005194	CEBPB	0.0029454
7904869	NM_001130111	FAM108A1	0.0029715
7904948	NM_001130111	FAM108A1	0.0029715
8089261	NM_170662	CBLB	0.0029816
8021091	NM_213602	SIGLEC15	0.0029869

7931029	ENST00000365014		0.0029979
7967493	NM_006312	NCOR2	0.0030257
8095110	NM_000222	KIT	0.0030346
7945536	NM_016564	CEND1	0.0030513
7940914	NM_004470	FKBP2	0.0030574
8037272	NM_002781	PSG5	0.003063
8013159	NM_001082968	TOM1L2	0.0030739
7918426	NM_004696	SLC16A4	0.0030966
7946807	NM_021029	RPL36A	0.0031141
7940775	NM_004585	RARRES3	0.0031171
7983527	NM_153618	SEMA6D	0.0031205
8072160	NM_032173	ZNRF3	0.0031213
7927548	NM_006327	TIMM23	0.0031474
8105338	ENST00000384500		0.0031719
8091735	NM_001168214	C3orf80	0.0031735
8021169	NM_006033	LIPG	0.0031887
8032608	NM_174983	C19orf28	0.0032087
7896370			0.0032407
8108631	NR_026705	VTRNA1-3	0.0032616
8122982	NM_024630	ZDHHC14	0.0032717
8135876	NM_014390	SND1	0.0032832
7908407	NM_002954	RPS27A	0.0032934
7896026			0.0033022
7965767	ENST00000384063		0.0033046
8114287	NM_004598	SPOCK1	0.003308
7974533	NM_021255	PELI2	0.0033184
8122176	NM_003206	TCF21	0.0033184
8088550	NM_198859	PRICKLE2	0.0033197
8081810	NM_001130064	GAP43	0.0033349
8097120	ENST00000363683		0.0033357
7898209	ENST00000420030		0.0033849
7892532			0.0033857
8074773	NM_001017964	YDJC	0.0033956
8127563	NM_004370	COL12A1	0.0034021
8156571	NR_029665	MIR27B	0.003432
7999149	NM_020677	NMRAL1	0.0034469
8111913	ENST00000362617		0.0034687
8134257	NM_004126	GNG11	0.0035068
8075820	NM_006078	CACNG2	0.0035374
7981980	NR_003331	SNORD116-16	0.0035438
7962895	NM_016594	FKBP11	0.0035459
7927936	NM_004728	DDX21	0.003552
7937518	NM_001025237	TSPAN4	0.0035815
8096224	NM_001166693	AFF1	0.0035867
8180404	NM_000990	RPL27A	0.0036057
8165406	NM_015392	NPDC1	0.0036179
8053449	NM_001031738	TMEM150A	0.0036225
7910022	NM_152495	CNIH3	0.0036383
7920337	NM_023015	INTS3	0.0036487
8051012	NM_013388	PREB	0.0036543
8102410	NM_001029	RPS26	0.0036586
8023727	NM_032160	DSEL	0.0036661
8157761	NM_001145001	NEK6	0.0036829
8098441	NM_001080477	ODZ3	0.0036974
8134589	NM_003910	BUD31	0.0037193

7896547			0.0037392
8103873			0.0037396
8148737	NM_032272	MAF1	0.0037573
8114805	NM_000800	FGF1	0.0037981
8141664	NM_003378	VGF	0.003808
7959085	ENST00000362939		0.0038105
8088397	NM_003500	ACOX2	0.0038358
7942342	NM_001567	INPPL1	0.0038789
7895814			0.003892
8063174	ENST00000442886		0.0038931
8156761	NM_018946	NANS	0.0038961
8102321	NM_030821	PLA2G12A	0.0039134
7992905	NM_001083601	NAA60	0.0039149
8070567	NM_003226	TFF3	0.0039224
8083494	NM_007288	MME	0.003936
7895674			0.0039366
7895010			0.003938
8149281			0.003979
7917728	NM_001006605	FAM69A	0.0039862
8056100	ENST00000384389		0.0040083
7896542			0.0040097
7966690	NM_016569	TBX3	0.0040198
8130993	NM_020223	FAM20C	0.004034
8127193	NM_021073	BMP5	0.00405
7963670	NM_006301	MAP3K12	0.004061
7963774	NM_015481	ZNF385A	0.0040629
8005695	NR_002211	MEIS3P1	0.0040697
7896485			0.0040975
8075322	NM_001037666	GATSL3	0.0041221
8036936	AK096566	LOC100130713	0.0041408
7995040	NM_001122957	BCKDK	0.0041434
7975626	NM_194278	C14orf43	0.0041446
8138361	NM_021029	RPL36A	0.0041451
8125123	ENST00000364685		0.0041493
8013384	NM_000691	ALDH3A1	0.0041496
8099967	NM_001098634	RBM47	0.0041662
8018264	NM_030630	C17orf28	0.0042058
8139758	NM_016139	CHCHD2	0.00421
7967463	NM_178314	RILPL1	0.0042213
7894570			0.004223
7970810	NM_003045	SLC7A1	0.004239
8107632	NM_014035	SNX24	0.0042913
7924071	NM_172362	KCNH1	0.0042944
8088996			0.004304
7919055	NM_005518	HMGCS2	0.0043089
8121861	NM_181782	NCOA7	0.0043167
7918857	NM_005725	TSPAN2	0.0043369
7929919	NM_030971	SFXN3	0.0043518
7895261			0.0043532
7975066	NM_004857	AKAP5	0.0043567
8113873	NM_005340	HINT1	0.0043757
8095751	NM_015393	PARM1	0.004379
8101701	NM_152542	PPM1K	0.0043877
8079993	NM_007022	CYB561D2	0.0043956
8123985	NM_016167	NOL7	0.0044326

8037642	NM_177542	SNRPD2	0.0044477
8165817	NM_001079855	GYG2	0.0044659
8172305	ENST00000364580		0.004502
7894057			0.0045036
8153835	NM_032902	PPP1R16A	0.0045211
8008454	NM_003786	ABCC3	0.0045344
8154135	NM_004170	SLC1A1	0.0045496
8138224	NM_002489	NDUFA4	0.0045504
8136557	NM_001130966	TBXAS1	0.0045519
7979611	NM_006977	ZBTB25	0.0045961
7941382	NM_182710	KAT5	0.004606
8012274	NM_032356	LSMD1	0.0046084
8123606	BC025340	MGC39372	0.0046137
8045229	NM_032995	ARHGEF4	0.0046153
7893001			0.0046278
7978272	NM_014430	CIDEB	0.0046326
7950731	NM_199418	PRCP	0.0046444
8133662	NM_001040457	RHBDD2	0.004732
7908351	NM_024420	PLA2G4A	0.0047439
8007828	NM_016835	MAPT	0.004795
7950701	NM_001098816	ODZ4	0.0047998
7897236	NM_003636	KCNAB2	0.0048166
8077652	NM_005718	ARPC4	0.0048713
8146896	ENST00000384309		0.0048796
7893057			0.0048969
8012000	NM_001004333	RNASEK	0.0049052
7984259	NR_002757	RNU5B-1	0.0049107
7971690	ENST00000410988		0.0049157
8152119	NM_001040624	NCALD	0.0049243
7896453			0.0049392
7963206			0.0049681
7968236	NM_206827	RASL11A	0.0049966
8013341	NM_002404	MFAP4	0.0050082
7894017			0.0050359
8104930	NM_004172	SLC1A3	0.0050892
8172573	NM_003179	SYP	0.0052055
8174710	NM_001000	RPL39	0.0052143
7908459	NM_000186	CFH	0.0052408
7905329	NM_006818	MLLT11	0.0052687
8026971	NM_006332	IFI30	0.0053024
8005289	NM_001130090	LRRC48	0.0053057
7948987	NM_007069	PLA2G16	0.0053094
8034448	NM_016145	C19orf56	0.0053192
7973867			0.0053217
8089038	NC_001807		0.0053257
8112914	NC_001807		0.0053257
8131496	NM_138426	GLCCI1	0.0053459
7896688			0.0053503
8127526	NM_001000	RPL39	0.0053567
8134036	NM_152999	STEAP2	0.0053724
7895859			0.0053752
7927964	NM_002727	SRGN	0.0053871
7955502	NM_014191	SCN8A	0.0054001
8092230	NM_022470	ZMAT3	0.0054272
8034783	NM_001008701	LPHN1	0.0054407

7997542	NM_019065	NECAB2	0.0054452
8180299	NM_021144	PSIP1	0.0054517
8086148	NM_000992	RPL29	0.0054523
8144503	ENST00000310542		0.0055113
8014115	NM_015194	MYO1D	0.005519
8167165	NM_001654	ARAF	0.0055452
7896378			0.005548
7972577	NM_001029	RPS26	0.0056414
7924307	ENST00000365628		0.0056535
8148553	NM_017527	LY6K	0.0056544
8042310	NM_003038	SLC1A4	0.0056797
8161192	NM_194328	RNF38	0.0056957
8061428	NM_001247	ENTPD6	0.0057289
7901046	NR_000015	SNORD55	0.0057488
7941587	NM_182553	CNIH2	0.0057669
8128109			0.0057703
7893259			0.0057879
7946436	NM_020646	ASCL3	0.0058056
7892720			0.0058107
7905817	NM_000748	CHRNB2	0.0058391
7894705			0.0058993
8052598	NM_015910	WDPCP	0.0059079
8001656	ENST00000384444		0.005941
8166289	NM_001037343	CDKL5	0.0059474
8133688	NR_002955	SNORA14A	0.005986
8136591	NM_052853	ADCK2	0.0059885
8111136	NM_001034850	FAM134B	0.0060017
7899955	ENST00000364998		0.0060121
8034108	NM_024029	YIPF2	0.0060146
7965231	NM_013244	MGAT4C	0.0060151
7939839	NM_002843	PTPRJ	0.006024
7899375	ENST00000411130		0.0060689
7952126	NR_003040	RPL23AP64	0.0060744
7892671			0.0060766
7946149	NM_001164	APBB1	0.0060977
7908694	NM_020443	NAV1	0.0061089
7955076	ENST00000419998		0.006115
7940070	NM_170746	C11orf31	0.0061363
7896339			0.0061573
7893764			0.0061604
8048112	NM_001080500	VWC2L	0.0061659
7989037	NM_004748	CCPG1	0.006171
8084945	ENST00000439929		0.0061782
7941148	NM_003273	TM7SF2	0.006191
8161648	NM_001206	KLF9	0.0061993
7893295			0.0062019
7908758	NM_198149	SHISA4	0.0062045
7985240	NM_007364	TMED3	0.0062196
7893239			0.0062203
7967727	NM_021808	GALNT9	0.0062387
7997827	NM_001142864	PIEZO1	0.0062735
7895176			0.0062787
8039805	NM_003969	UBE2M	0.0062796
8147000	NM_024721	ZFHX4	0.0063667
8114787	NM_005471	GNPDA1	0.0063747

8047852	ENST00000363894		0.0063784
8121613	NM_016104	RWDD1	0.0063884
8001007	NM_002773	PRSS8	0.0063956
7994576	NR_002939	RUNDC2C	0.0064154
7917944	NR_029679	MIR137	0.0064208
8137874	NM_032415	CARD11	0.0064234
8089835	NM_007085	FSTL1	0.006438
8164129			0.0064383
8087119	NM_022911	SLC26A6	0.0064579
7906185	NM_144772	APOA1BP	0.0064695
8089285			0.0064722
8041940	NM_172311	STON1-GTF2A1L	0.006484
7976766	NM_024515	WDR25	0.006489
7892683			0.0064894
7894741			0.0064934
8081055	NM_014043	CHMP2B	0.0065089
7968928	NM_002901	RCN1	0.0065375
7906417	NM_021189	CADM3	0.0065397
8089647	NM_001009899	KIAA2018	0.0065398
8172158	NM_003688	CASK	0.0065542
7894525			0.0065959
8027002	NM_004864	GDF15	0.0066035
8114709	NM_033449	FCHSD1	0.0066465
8150276	NM_001102559	PPAPDC1B	0.0066775
7902382	NM_004582	RABGGTB	0.006693
8037835	NM_005628	SLC1A5	0.0066941
8135587	NM_001233	CAV2	0.0067095
7968254	NM_152705	POLR1D	0.0067511
8096176	NM_080683	PTPN13	0.0067579
8036557			0.0067614
8035859			0.0067703
8014454	NM_001163735	MYO19	0.0067762
7998033	NM_014972	TCF25	0.0067862
7989491	AB209041	TPM1	0.0067962
7981714	ENST00000333301		0.0068126
7896730			0.0068442
7995803	AF348994	MT1JP	0.0068556
7994609	NM_014298	QPRT	0.0068646
8074958			0.0068764
8081288	NM_018004	TMEM45A	0.0069001
8085300	NR_024272	SEC13	0.0069075
8041467	NM_053276	VIT	0.0069468
7924230	NM_001130111	FAM108A1	0.0070222
7894698			0.0070275
8133818	NM_001127358	PHTF2	0.0070358
7896348			0.0070784
8122426	NM_014721	PHACTR2	0.0070785
8075785	NM_024955	FOXRED2	0.0070889
7895096			0.0071277
7976834	NR_030174	MIR494	0.0071369
7899502	NR_004407	RNU11	0.007144
8123137	NM_005891	ACAT2	0.0071488
7921344	NM_012081	ELL2	0.0071667
7893166			0.0071749
8008825	NM_024612	DHX40	0.0071768

8009561	NM_000999	RPL38	0.0072054
8110055	NM_030627	CPEB4	0.0072147
8064868	NM_019593	GPCPD1	0.0072173
8071559	NM_022044	SDF2L1	0.0072309
7998367	NM_058192	RPUSD1	0.0072436
7906602	NM_020335	VANGL2	0.0072921
8063337	NR_003605	ZNFX1-AS1	0.0073173
7978335	NM_020195	SDR39U1	0.0073254
8121214	ENST00000384290		0.0073516
7949650	NM_003793	CTSF	0.0073702
7895365			0.007399
8173513	NM_001007	RPS4X	0.0074312
8130598	NR_028093	LPAL2	0.0075129
8148715	NM_003801	GPAA1	0.0075371
7895787			0.0075606
8094789	NM_014988	LIMCH1	0.0075775
8138547	NM_019059	TOMM7	0.0075893
8074606	NM_017414	USP18	0.007601
7911862	NM_020710	LRRC47	0.0076018
8113220	NM_012081	ELL2	0.0076094
7951703	NM_000795	DRD2	0.0076224
8073104	NM_004711	SYNGR1	0.0076868
8180310	NM_005494	DNAJB6	0.0076961
8089402	NM_005459	GUCA1C	0.0076969
8048628	NM_001005209	TMEM198	0.0077155
7991138	NM_021077	NMB	0.0077396
7987225	NM_018648	NOP10	0.0077452
7945958	NM_001005161	OR52B4	0.0077701
7895064			0.0077751
8107920	NM_003060	SLC22A5	0.0077827
8167763	NM_022117	TSPYL2	0.007813
8070953	NM_001142854	C21orf56	0.0078315
7893294			0.007904
8144719	ENST00000363760		0.0079295
8085287	NM_018462	BRK1	0.0079389
8059648	ENST00000362530		0.007941
7979416	NM_012460	TIMM9	0.0079563
7999217	NM_024589	ROGDI	0.0079827
7914042	NM_004672	MAP3K6	0.0079903
8105084	NM_000587	C7	0.0080028
8025051	NM_006087	TUBB4	0.0080397
8058458	ENST00000327138		0.0080595
7997680	NM_014615	KIAA0182	0.0080636
8110427	ENST00000407744		0.0081245
8076365	NM_015704	PPPDE2	0.0081622
8000702	NM_175900	C16orf54	0.0081745
8003601	NM_175900	C16orf54	0.0081745
8171435	NM_003662	PIR	0.0082024
8122816	ENST00000411107		0.0082494
7980718	GENSCAN00000063635		0.0082619
8019357	NM_016286	DCXR	0.0082714
7923438	NM_001030	RPS27	0.0082988
7970716	NM_153371	LNX2	0.0083064
7952599	ENST00000410842		0.0083198
8166230	NM_018360	TXLNG	0.0083222

8007058	NM_133264	WIPF2	0.0083228
7949948	NM_022338	C11orf24	0.0083275
7893340			0.0083385
8162490	NM_032558	HIATL1	0.0083429
7965573	NM_021229	NTN4	0.0083946
7932864	NM_183058	LYZL2	0.0084159
7983704	NM_181789	GLDN	0.0084169
7949377	NM_001997	FAU	0.0084609
8094359			0.0084698
7893765			0.0085144
7956271	NM_003725	HSD17B6	0.0085248
7893573			0.0085459
7896067			0.0085503
7981960	NR_003321	SNORD116-6	0.0085801
8052798	NM_014911	AAK1	0.0086029
8070083	NM_006134	TMEM50B	0.008638
7900540	NM_173642	RIMKLA	0.0086415
8080810	NM_002841	PTPRG	0.008657
7909283	NM_018566	YOD1	0.0087128
7945803	NM_001014438		0.0087152
8100541	NM_001553	IGFBP7	0.0087415
8043909	NM_002518	NPAS2	0.0087618
8126750	NM_021572	ENPP5	0.0087677
8005547	NR_006880	SNORD3A	0.0087925
8005553	NR_006880	SNORD3A	0.0087925
8013323	NR_006880	SNORD3A	0.0087925
8013325	NR_006880	SNORD3A	0.0087925
8013329	NR_006880	SNORD3A	0.0087925
8074030	NM_014678	PPP6R2	0.0088015
7896535			0.0088063
7922908	ENST00000384380		0.0088439
7905733	NM_006118	HAX1	0.0089351
8092473	NM_004366	CLCN2	0.0089557
8161056	NM_006289	TLN1	0.0089967
8047078	NM_017694	MFSD6	0.0090105
7898677	NM_004807	HS6ST1	0.0090346
7956159	NM_001035267	RPL41	0.0090373
8180283	NM_033251	RPL13	0.00904
7963333	NM_182507	KRT80	0.0090581
8139500	NM_022748	TNS3	0.0090773
8086419	NR_027753	HHATL	0.0091118
8137526	NM_005542	INSIG1	0.0091246
8059111	NM_005689	ABCB6	0.0091554
8089314	NM_018010	IFT57	0.0091588
7904480	ENST00000383967		0.009167
7904963	ENST00000383967		0.009167
7896109			0.0091766
8150704	NM_144651	PXDNL	0.009179
7965152	ENST00000427438		0.0091818
7945539	NM_024698	SLC25A22	0.0091839
7894160			0.0091907
7913694	NM_000147	FUCA1	0.0092075
8079058	ENST00000410940		0.0092195
8037816	NM_013403	STRN4	0.0092443
8127484	NM_080742	B3GAT2	0.0093194

7981982	NR_003332	SNORD116-17	0.0093376
7981986	NR_003332	SNORD116-17	0.0093376
7941260	NM_031904	FRMD8	0.0093884
8007154	NM_021939	FKBP10	0.0094161
8083901	NM_022763	FNDC3B	0.0094399
8154363	NM_001029	RPS26	0.0094729
8047487	NM_003507	FZD7	0.0094739
7897803	NM_000302	PLOD1	0.0095051
8156404	NM_032310	C9orf89	0.0095616
7921862	NM_001013625	C1orf192	0.0095754
7920971	NM_144580	C1orf85	0.0095847
8061184	ENST00000398663		0.0095913
7909236	NM_004759	MAPKAPK2	0.009603
7974341	NM_053064	GNG2	0.0096032
8073949	NM_001135101	CRELD2	0.0096883
8009145	NM_030779	KCNH6	0.0096922
8176230	NM_171998	RAB39B	0.0097433
8180346	NM_000581	GPX1	0.0098058
8078012	ENST00000362739		0.0098315
7898679	NM_032264	NBPF3	0.0098447
8124397	NM_005319	HIST1H1C	0.0098462
8038202	NM_001190	BCAT2	0.0098559
8028286	NM_015073	SIPA1L3	0.0098639
8073578	BC024237	C22orf32	0.0098675
8055872	NM_000726	CACNB4	0.009868
8077160	NM_000487	ARSA	0.0099043
8060030	NM_006037	HDAC4	0.0099054
7972021	NM_014832	TBC1D4	0.0099158
8110022	NM_003945	ATP6V0E1	0.0099415
7969596	NM_004466	GPC5	0.0099448
7967789	NM_018663	PXMP2	0.0099634
7994675	NM_181718	ASPHD1	0.0099758
7896533			0.009997
